# Single-Component Adsorption Equilibria of CO_2_, CH_4_, Water, and Acetone on Tapered Porous Carbon Molecular Sieves

**DOI:** 10.1021/acs.jced.3c00368

**Published:** 2024-02-22

**Authors:** Ojuolape O. Oghenetega, Pasquale Fulvio, N. Scott Bobbitt, Krista S. Walton

**Affiliations:** † School of Chemical & Biomolecular Engineering Georgia Institute of Technology, Atlanta, Georgia 30332, United States; ‡ Sandia National Laboratories, Albuquerque, New Mexico 87185, United States

## Abstract

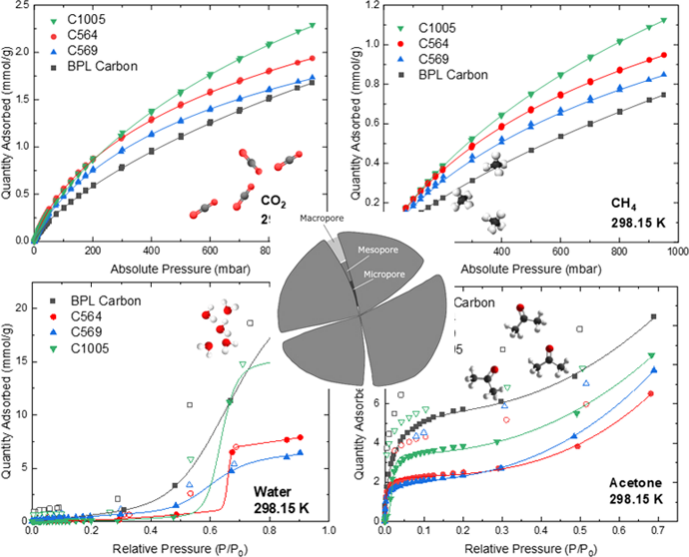

Engineered carbon molecular sieves (CMSs) with tapered pores, high surface area, and high total pore volume were investigated for their CO_2_, CH_4_, water, and acetone adsorption properties at 288.15, 298.15, 308.15 K, and pressures of <1 bar. The results were compared with BPL carbon. The samples exhibited higher adsorption capacity for CO_2_ compared to BPL carbon, with Carboxen 1005 being the highest due to the presence of ultramicropores (pores smaller than 0.8 nm). Similar observations were made for CH_4_ except at 288.15 K. Although the CMSs exhibited higher hydrophobicity than BPL carbon, the latter had the highest acetone uptake for all investigated temperatures due to its higher oxygen content, which facilitates stronger interactions with polar VOC molecules. Heats of adsorption were calculated using the Clausius–Clapeyron equation after fitting the isotherms with the dual-site Langmuir–Freundlich model, and results largely corroborated the order of adsorption capacities of CO_2_, CH_4_, and water on the carbon materials.

## Introduction

The adsorption properties of porous carbons have been widely investigated for CO_2_, CH_4_, and some volatile organic compounds (VOCs) for mitigating carbon emissions and for commercial and domestic air purification.^1,2^ Rigorous studies have been carried out to analyze the adsorption of VOCs on these materials and, hence, the regular commercial use of porous carbons for gas separation and purification. Some of the most commonly captured solvents include acetone, heptane, toluene, and hexane; carbon tetrachloride, methylene chloride, ethyl acetate, naphthalene, and methyl ethyl ketone (MEK), among others.^3^

The VOC adsorption capacity of activated carbon is dependent on its physicochemical properties such as surface area, pore size, pore volume, surface functional groups, and so on, VOC properties such as molecular size and polarity, and adsorption conditions such as concentration, temperature, humidity, and pressure. However, there are certain limitations to the adsorption of VOCs on activated carbon. The hydrophobic nature of its surface restricts the adsorption of hydrophilic or polar VOCs. Additionally, the microporosity of these carbons would prevent VOCs with larger molecule sizes from entering the pores, and the irregular pore structure can increase diffusion resistance of VOC molecules within the material.^1^ Furthermore, high-temperature VOC adsorption has been found to cause the porous structure of activated carbons to collapse or spontaneous ignition of the material.^4^

This increase in adsorption loading with a decrease in polarity of VOCs was observed in BAX950, a wood-based activated carbon. The uptake of the molecules at low pressures increased in the order of methanol, ethanol, propanol, butanol, *n*-octane, and *n*-nonane, respectively.^5^ Molecular simulation studies of acetone adsorption on functionalized activated carbon showed that adsorption was influenced by the concentration of acetone, pore width, the oxygen content of the material, and the type of functional group(s) present. It was deduced that with a low concentration of acetone (∼10 ppmv) and 5 wt % of oxygen present on the surface, acetone prefers to adsorb in pores less than 1 nm, with oxygen functional groups present, especially hydroxyl and carboxyl. However, as the weight percent of oxygen increases, the pores are filled with functional groups and loss of uptake is observed.^6^

In many industrial or environmental applications, the presence of atmospheric gases such as CO_2_, CH_4_, and water vapor can have a significant impact on the VOC adsorption behavior of carbon materials. For example, water can compete with VOCs for adsorption sites on the surface of activated carbon, and it can also block the pores and reduce the surface area available for adsorption.^7^ CO_2_ can also interact with the surface functional groups of activated carbon and fill the micropores, which can affect its adsorption properties.^8^ Therefore, it is important to analyze how these molecules adsorb on carbon materials and how they impact the adsorption of VOCs.

Moreover, understanding the interactions between these molecules and activated carbon can also facilitate the development of more effective methods for the selective removal of VOCs from gas mixtures. For instance, the use of modified or functionalized activated carbon materials can enhance their selectivity and adsorption capacity for specific VOCs, thereby enabling more efficient and cost-effective VOC removal from gas mixtures that contain other molecules.^7^ Therefore, this knowledge can help in the design of more efficient carbon-based adsorbents for breath analysis, VOC abatement, separations, and so on.

The IUPAC classifies pores based on size with micropores having pore diameters smaller than 2 nm, mesopores between 2 and 50 nm, and macropores having pore diameters larger than 50 nm. Research efforts over the years have focused on the development of carbons from both natural and synthetic precursors, and their resulting adsorption and surface properties for the adsorption of specific gas molecules found in post-combustion feeds and VOCs. A more detailed understanding of the adsorption properties of commercially available carbons, however, is still required for selecting materials for the adsorption and separation of gases with very distinct polarities in multicomponent feeds. An example is the presence of water, which can impact the adsorption of CO_2_, CH_4_, and VOCs by carbon materials.^9^

Although multicomponent adsorption studies are difficult to perform, single-component adsorption equilibrium data can be used to predict multicomponent adsorption selectivity with mixture models such as the ideal adsorbed solution theory (IAST).^10^ Moreover, the heat of adsorption is a valuable parameter for describing the temperature-dependent interaction of gases with surfaces.^11^ The Langmuir isotherm model is widely used for heat of adsorption and IAST calculations for gases exhibiting a Type I isotherm. This model assumes that all gas molecules adsorb on energetically homogeneous surfaces and therefore assumes constant heats of adsorption at all adsorption pressures.^12^ The latter assumptions are often valid only for low pressures or simply for monolayer adsorption.

A more accurate model for modeling the adsorption behavior of gases that strongly interact with the sorbent surface is the Freundlich isotherm, which is valid for describing multilayer adsorption. In multilayer adsorption, the isotherm has a positive slope, indicative of multilayer formation without capillary condensation. The Freundlich isotherm was originally derived empirically, and it was later described by attributing changes in the equilibrium constant to surface heterogeneity of the sorbent and to variations in the heats of adsorption.^13^ Most materials, however, are heterogeneous and preferred adsorption sites exist, given the existence of surface defects or of surface functional groups. Thus, a dual-site Langmuir (DSL) model that considers two types of sites with different adsorption energies often provides a better fit of the experimental adsorption data for gases exhibiting Type I isotherms.^14^ Similarly, the dual-site Langmuir–Freundlich (DSLF) model further extends the latter to include the multilayer range of adsorption. Both DSL and DSLF isotherms can be used for predicting multicomponent adsorption according to IAST.

The molecules of interest in this study are commonly found in exhaled breath. Typically, a person’s exhaled breath contains 78.04% nitrogen, 16% oxygen, and 4–5% carbon dioxide and is fully saturated with water vapor. Both methane and acetone are listed as some of the most important disease biomarkers in the human body.^15^ For instance, methane has been identified as a biomarker for small intestine bacterial overgrowth and for the intestinal methanogen overgrowth.^16,17^ Acetone, on the other hand, is generally present in exhaled breath;^15^ however, unusual levels could indicate diabetes,^18^ lung cancer,^19^ and nutritional-related disorders^19^ within a patient. Exploring these materials for a prominent VOC biomarker such as acetone can give insights into the possible use of adsorbents for creating diagnostic breath tests.

In this work, DSL and DSLF isotherms and the heats of adsorption for CO_2_, CH_4_, water, and acetone were investigated for a series of commercially available porous carbons known as carbon molecular sieves (CMSs) due to their small pore widths and almost unimodal pore size distribution of micropores. The selected carbons were Carboxen 564, Carboxen 569, and Carboxen 1005. These carbons possess surface areas ranging from 400 to 1000 m^2^ g^–1^ and are available as powders or as pre-formed spheres, making them suitable for packing columns. Moreover, these Carboxens are uniquely designed beads with a mesh size of 20/45 having hierarchical pore structures tapered from macropore to mesopore to micropore. Tapered pores can best be described as pores that decrease in width along their lengths. This implies that these pores are in the macropore range near the particle surfaces and then narrow down to a mesopore range until they reach the micropore range near the more central regions of the particles. The well-known benchmark activated carbon, BPL carbon, was also investigated for comparison with the CMSs. The adsorption results are discussed with respect to the calculated adsorption parameters from N_2_ at 77K isotherms and the surface chemical composition of these materials obtained from X-ray photoelectron spectroscopy (XPS).

## Experimental Section

### Materials and Gases

Three commercial carbon molecular sieves Carboxen 564, Carboxen 569, and Carboxen 1005 (denoted as C564, C569, and C1005, respectively) were purchased from Supelco. BPL carbon 4 × 6 was purchased from Calgon Carbon Corporation. The specifications of all materials used are summarized in Table 1. The three gases used for the adsorption experiments include ultrahigh purity N_2_ and CH_4_ as well as bone dry CO_2_. The gases and vapors used are also listed in Table 1 along with their suppliers and purities.

**Table 1:** Specifications of the Chemicals/Materials Used: Supplier, Percent Purity, and CAS Number

chemical name	supplier	percent purity	CAS number
BPL carbon	Calgon	not reported	7440-44-0
C564	Supelco	not reported	7440-44-0
C569	Supelco	not reported	7440-44-0
C1005	Supelco	not reported	7440-44-0
CH_4_	Airgas	99	74-82-8
N_2_	Airgas	99	7727-37-9
CO_2_	Airgas	99	124-38-9
Acetone	Sigma-Aldrich	reagent grade, ≥98% (HPLC)	67-64-1

### Characterization

N_2_ and CO_2_ sorption isotherms were measured at 77 and 273.15 K, respectively, using a Micromeritics 3Flex volumetric analyzer (Micromeritics, Norcross, GA). Samples were outgassed under vacuum and at 200 °C for at least 24 h. The specific surface areas (BET) were calculated within the relative pressure range 0.01–0.1.^20^ The total or single-point pore volumes were obtained directly from the N_2_ adsorption isotherms at a relative pressure of 0.995. The *t*-plot analysis was performed using the carbon black statistical thickness surface area (STSA) equation. The micropore volumes and surface areas were obtained by the linear fitting of the *t*-curves within the statistical film thickness range of 0.35 and 5.0 nm.^21^ The external surface areas were obtained by difference from the total surface areas and where the mesopores in these materials were treated as textural (external) pores. The pore size distributions (PSDs) and cumulative pore volumes were calculated using nonlocal density functional theory (NLDFT) assuming a mixed model of slit and cylindrical pores (HS-2D-NLDFT) from the N_2_ adsorption data. The ultramicropore volumes were calculated by using Grand Canonical Monte Carlo (GCMC) simulations. The PSD curves were treated to the same 10^–1^ level of regularization using the MicroActive software. These data are tabulated in the SI in Tables S1–S4.

X-ray photoelectron spectroscopy (XPS) analysis was performed using a Thermo K-Alpha instrument (Thermo Fisher Scientific). Prior to measurements, the samples were placed onto a Cu grid substrate and outgassed under a vacuum and room temperature for 5 days. The pass energy for the C 1s line was of 0.05 and 0.1 eV for the Si 2p, S 2p, and O 1s lines. For each sample, 10 spectra were collected for the narrow regions. Samples were etched with an X-ray gun for 60 s to eliminate contribution from adventitious carbon; the spectra of samples without etching and etched for 30 s were also collected for verification of the consistency of the quantitative analysis. The neutralizing Ar-ion gun was used for the entirety of each measurement to eliminate charging effects. Spectral fittings for the latter were performed by using the Avantage software package. Peak fitting of the narrow region spectra was performed using a Shirley-type background, and the synthetic peaks were calculated by the Powell method for a Gaussian–Lorentzian mixed sum. XPS results are shown in Figures S5–S8.

The surface morphology was analyzed using a field emission scanning electron microscope (SEM), Hitachi SU-8230, which employs a novel cold field emission gun to produce high-resolution images. Elemental analysis of the surfaces captured by the SEM was performed by energy-dispersive X-ray spectrometry (EDS) (Figures S1–S4). All four materials were manually ground and degassed under ultrahigh vacuum for the XPS, SEM, and EDS characterizations.

### Adsorption Experiments

Single-component adsorption isotherms of CH_4_, CO_2_, water, and acetone were measured at temperatures of 288.15, 298.15, and 308.15 K on the four adsorbents using the Micromeritics 3flex volumetric adsorption analyzer. Water and acetone isotherms were collected over a relative pressure range (*P*/*P*_0_) of 0–0.95, while CO_2_ and CH_4_ isotherms were collected from 0 to 950 mbar absolute pressure. Approximately 50–80 mg of sample was used for each measurement on the instrument. Prior to adsorption measurements, the adsorbents were activated at 200 °C for 18 h under a vacuum. The instrument operates by dosing in gas or vapor to bring the pressure to the target pressure. The pressure drops as the sample adsorbs gas. The instrument measures the pressure at 10 equilibrium intervals. The weighted average pressure drop is calculated from the measurements. The rate of change of pressure (first derivative) at the central point (i.e., sixth measurement) is also calculated. If the rate of change of pressure is greater than 0.01% of the calculated weighted average, the instrument repeats the entire measurements again discarding the oldest or initial measurement. This is repeated until the measured pressure falls outside the tolerance band for the target pressure and then the instrument doses more gas or vapor to meet the target pressure. Or, the rate of pressure change is less than 0.01% of the calculated weighted average, which satisfies equilibrium conditions and the instrument advances to the next target pressure.

### Isotherm Model and Heat of Adsorption

The isotherms were fitted by using the DSL and DSLF models. The mathematical forms of these models are as follows
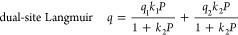
1
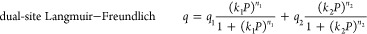
2where *q* (mmol g^–1^) is the amount adsorbed at equilibrium, *P* (mbar) is the equilibrium pressure, and *q*_1_, *q*_2_, *k*_1_, *k*_2_, *n*_1_, *n*_2_ are isotherm fitting parameters. CO_2_ and CH_4_ have been previously modeled successfully using DSLF as well as water vapor and benzene, another volatile organic compound.^22,23^ The Freundlich isotherm model is used for multilayer adsorption on heterogeneous sites, while the Langmuir isotherm is used for monolayer adsorption on homogeneous sites.^24^

The isosteric heat of adsorption was estimated from adsorption equilibrium data using the Clausius–Clapeyron equation, which assumes ideal gas behavior, the adsorbed and gas phases are at the same temperature, and the adsorbed phase volume is negligible.^25^
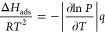
3
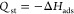
4where *T* (K) is temperature, *R* is the ideal gas constant with a value of 0.0083145 kJ mol^–1^ K^–1^, *P* is the equilibrium pressure, Δ*H*_ads_ is the enthalpy of adsorption, and *Q*_st_ is the isosteric heat. The interaction between adsorbate molecules and adsorbent surface atoms or groups is measured by isosteric enthalpy. This can be used to determine the energetic heterogeneity of a solid surface.

## Results and Discussion

### Material Characterization

**Figure 1:**
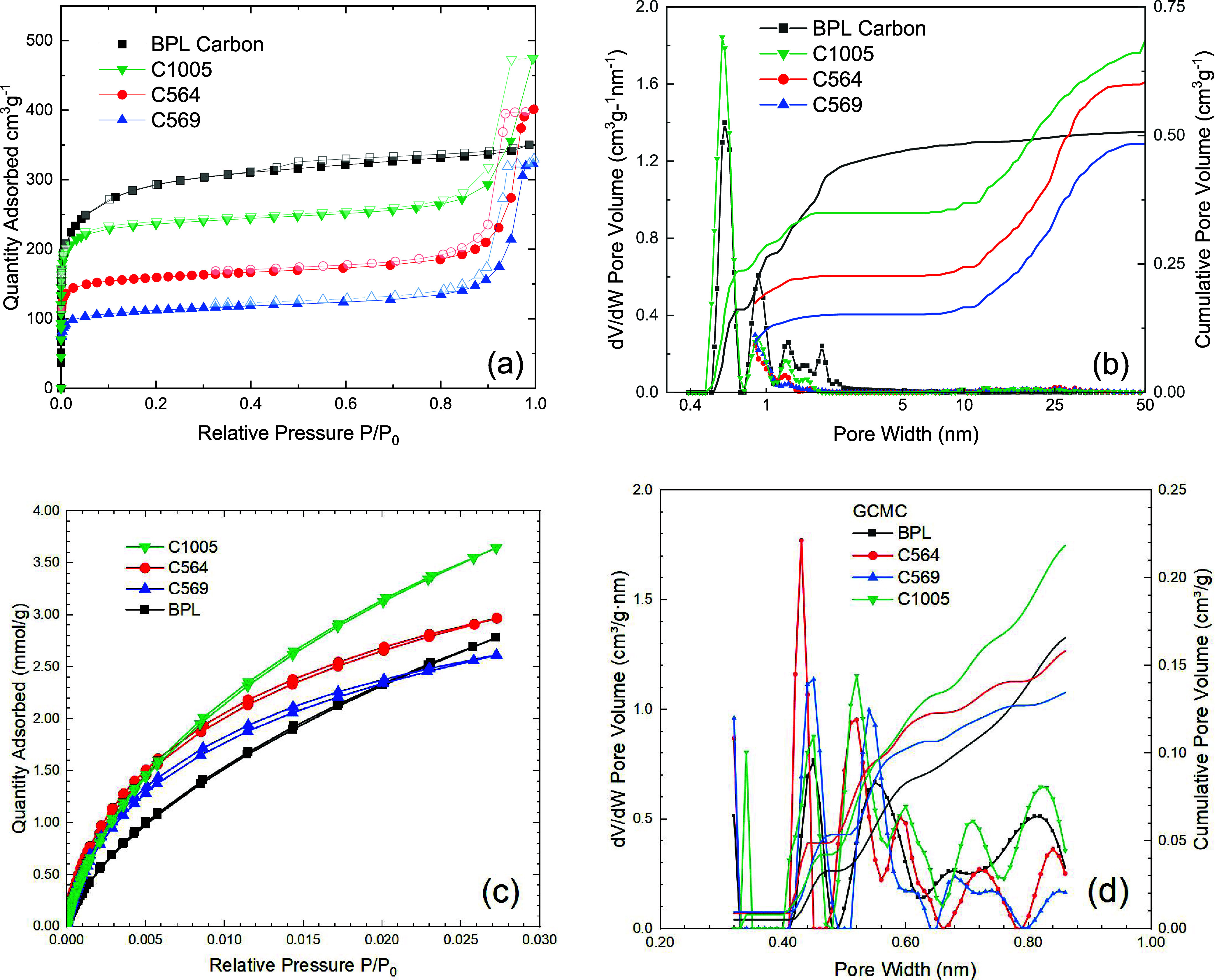
(a) N_2_ adsorption and desorption isotherms of BPL carbon, C564, C569, and C1005. Closed symbols represent adsorption data, and open symbols represent desorption data. (b) Corresponding pore size distributions with cumulative pore volumes from N_2_ data. (c) CO_2_ adsorption and desorption isotherms of BPL carbon, C564, C569, and C1005. (d) Corresponding pore size distributions with cumulative pore volumes for CO_2_ data.

The N_2_ adsorption data for BPL carbon (tabulated in Table S1) in Figure 1a exhibit a Type I adsorption isotherm with small H2 hysteresis, indicating a small volume of narrow mesopores with widths in the limit of capillary condensation. All Carboxen samples have a mixture of Type I and Type IV isotherms. These exhibit high gas uptake at low relative pressures, followed by H1 hysteresis occurring at relative pressures between 0.9 and 1.0. While the latter indicates large and well-developed mesopores potentially formed between uniform particle agglomerates, the former indicates the presence of micropores.^26^Figure 1c shows the CO_2_ adsorption isotherms with overlapping desorption data points. The calculated pore size distributions (PSDs) (Table S2) confirm the presence of micropores and mesopores as shown in Figure 1b and the PSD from the CO_2_ sorption data (Table S4) confirms the presence of ultramicropores as shown in Figure 1d.

The results indicate that all materials have small micropores as these contribute to the bulk of the surface areas of these materials. By substituting the volume and surface areas with those obtained from the *t*-plot, and that correspond only to micropores, smaller pore sizes of 0.8 nm were obtained. The PSD curves estimated using a mixture of slit and cylindrical pore geometries further corroborate that most micropores are ∼0.6 nm, which is in the range of ultramicropores (micropores smaller than 0.7 nm). The BPL sample has a larger fraction of micropores with a distribution centered at ∼1 nm, which is in the supermicropore range (0.7–2.0 nm).^27^ The CO_2_ pore volume analysis shows the distribution of pores <1 nm in the 4 carbon samples with the cumulative pore volume decreasing in order of C1005 > BPL carbon > C564 > C569. However, the PSD plots show that the Carboxens have a higher cumulative pore volume of smaller micropores <0.7 nm compared to BPL carbon. The analyzed pore volumes, BET surface areas, and average pore sizes for these materials are summarized in Table 2.

**Table 2:** Textural Properties of the Adsorbents^a^

	*S*_BET_ m^2^ g^–1^	*V*_SP_	*V*_mic_	*V*_umic_	*S*_mic_	*S*_ext_	*w*_d_ nm	*w*_t_ nm	*S*_BET_ m^2^ g^–1^	
adsorbent	(0.01 < *P*/*P*_0_ < 0.10)	cm^3^ g^–1^ (*P*/*P*_0_ ∼ 0.995)	cm^3^ g^–1^ (*t*-plot)	cm^3^ g^–1^ (<0.7 nm)	m^2^ g^–1^ (*t*-plot)	m^2^ g^–1^ (*t*-plot)	(average slit)	(average slit *t*-plot)	(reported)	ref
BPL	1078	0.54	0.38	0.10	900	178	1.00	0.84	1000	(28)
C564	605	0.62	0.20	0.13	503	102	2.04	0.80	400	(29)
C569	423	0.50	0.13	0.12	333	90	2.36	0.78	485	(29)
C1005	906	0.74	0.31	0.15	779	127	1.63	0.80	1150	(29)

a *S*_BET_ is the BET surface area; *V*_SP_ is the single-point pore volume; *V*_mic_ is the microporous volume calculated from the *t*-plot; *V*_umic_ is the ultramicroporous volume calculated from CO_2_ sorption data; *S*_mic_ is the microporous surface area; *S*_ext_ is the external surface area; *w*_d_ is the average pore size; *w*_t_ is the average pore size calculated from the *t*-plot.

**Figure 2:**
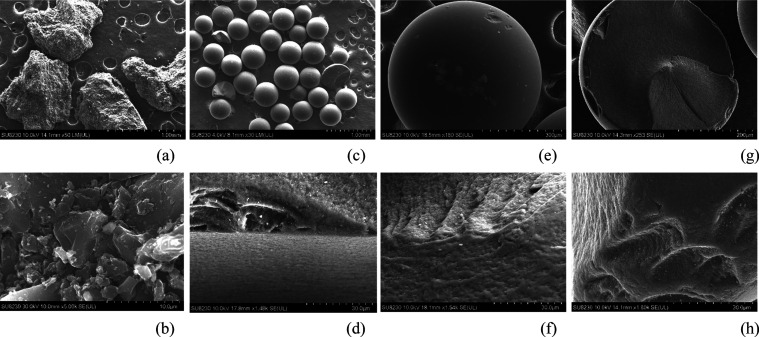
SEM images of (a) particles of BPL carbon, (b) magnified BPL carbon, (c) particles of C564, (d) magnified C564, (e) C569 particle, (f) magnified C569, (g) C1005 inner particle surface, and (h) magnified C1005.

The SEM micrographs of the carbons shown in Figure 2 were taken at different magnifications. At low magnification, BPL, Figure 2a,b, is composed of particles with random morphology and very large sizes. Some particles from noncarbonaceous inorganic impurities are also present in BPL.^30^ The surface roughness is indicative of the internal particle porosity. The CMS carbons are formed micron-sized spheres. The internal surfaces of these spheres have radial cracks from grinding these particles. While the outer surfaces are smooth, the interior of the particles has texture, also resulting from these having porous walls. All CMS materials have similar structures.

The compositions of the materials derived from EDS and XPS instruments are similar and are compared in Table 3. XPS analysis was carried out after etching for 60s and spectra obtained for all materials are shown in Figures S5–S8. The energy-dispersive X-ray (EDX) map analysis (shown in Figures S1–S4) was carried out at 30,000 kV and 15 mm working distance for all samples. The results showed that the samples had the same elements present except for Carboxen 569, which contained Mo, and BPL carbon, for which it detected Si as well as trace amounts of Al, Fe, Ca, Ti, and K. The detection of these elements (which make up 1.3%) is probably a result of impurities introduced during the manufacturing process of the BPL carbon. The high voltage resulted in a higher penetration of the sample for analysis compared to that of XPS. The sampled areas further differ between both techniques. Hence, small differences in the total wt % of elements between EDS and XPS result from the heterogeneity in the distribution of impurities.

**Table 3:** Elemental Composition of Carbons^a^

	Elemental Composition (wt %)							
sample	C_X_	C_E_	O_X_	O_E_	S_X_	S_E_	Si_X_	Si_E_
BPL carbon	87.11	91.40	7.67	5.70	1.69	0.90	2.68	0.80
C564	87.02	92.60	5.54	3.00	7.44	4.30		
C569	85.26	91.50	6.48	2.80	7.82	4.20		
C1005	91.39	94.70	5.08	2.30	3.53	3.00		

a The subscript “X” denotes results from XPS and the subscript “E” denotes results from EDX.

The C*1s* lines for these carbons have peaks at ∼284.5 eV attributed to the C sp^*2*^ and C sp^*3*^ carbons. Additional peaks at higher binding energies corroborate with the presence of oxygen functionalities in these materials. Agreement with the O 1s spectral analysis is found, where C=O and C–O bound to both aliphatic and aromatic groups were identified in addition to C–O–C and C–OH bonds. Some materials further exhibit peaks between 534.5 and 535.3 eV, attributed to O_2_/H_2_O.^31,32^ Moreover, a deconvoluted peak at 286.7 eV indicates the presence of C–S–C bonds.^32^ The S *2p* line for these carbons further corroborates with neutral S–S, S–C, and S–H bonds, with additional oxidized species of S–O–O–SO_3_ and −SO_4_.^33^ Finally, in agreement with EDS, the BPL sample had an additional Si*2p* line that was deconvoluted using a single peak centered at 103.1 eV and attributed to SiO_2_. However, for both EDS and XPS, the C1005 sample has the highest C wt % and the least detected amounts of O and S among the investigated materials. BPL carbon had the highest O content, which reached 7.67 wt % based on the XPS. This indicates a higher degree of surface functionalization with oxygen-bearing groups. The C569 sample had the second highest amount of O, closely followed by C564.

### Adsorption Isotherms

**Figure 3:**
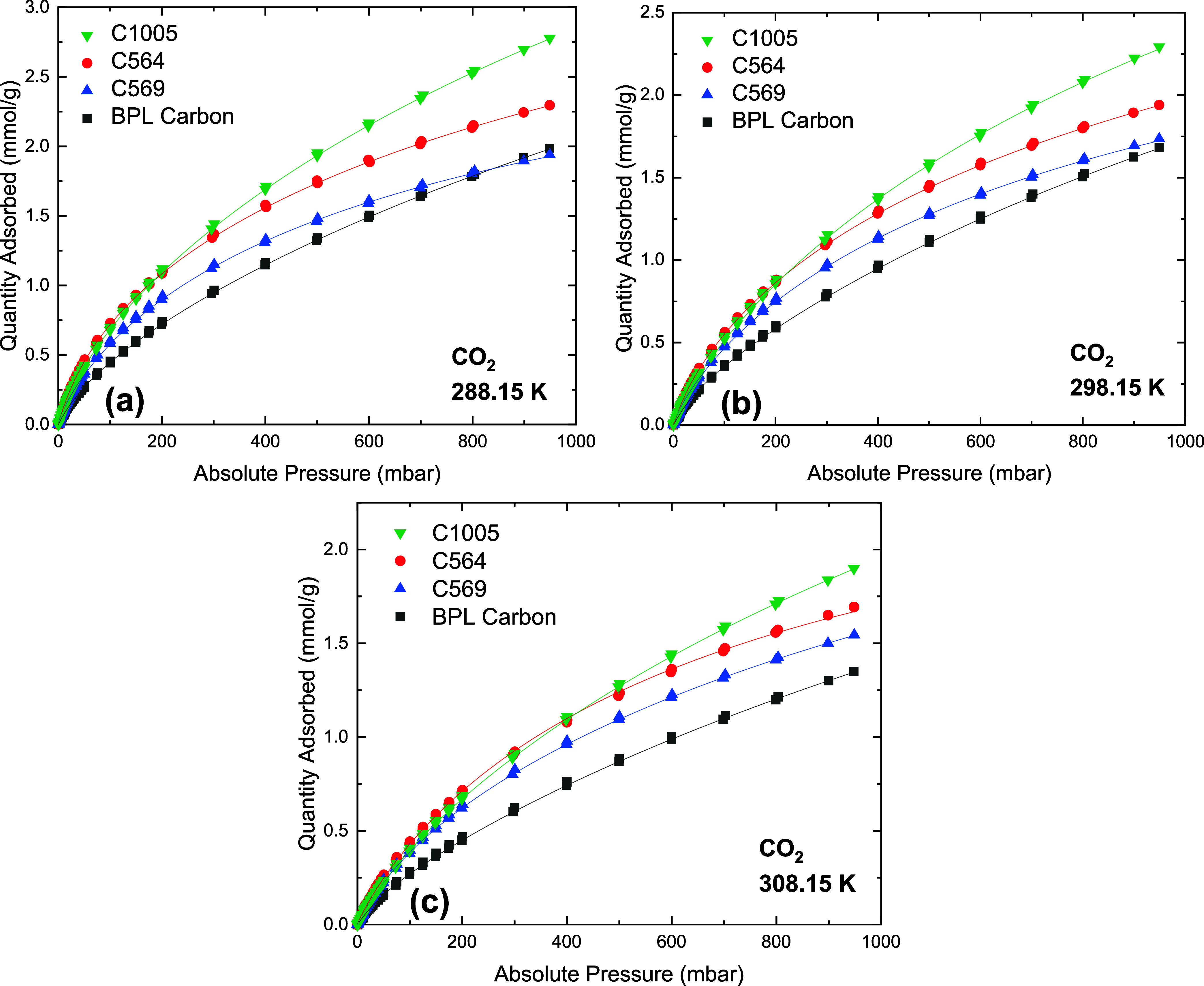
CO_2_ isotherms on carbon materials at (a) 288.15 K, (b) 298.15 K, and (c) 308.15 K. Closed symbols represent adsorption data, and lines represent DSLF model fitting.

**Figure 4:**
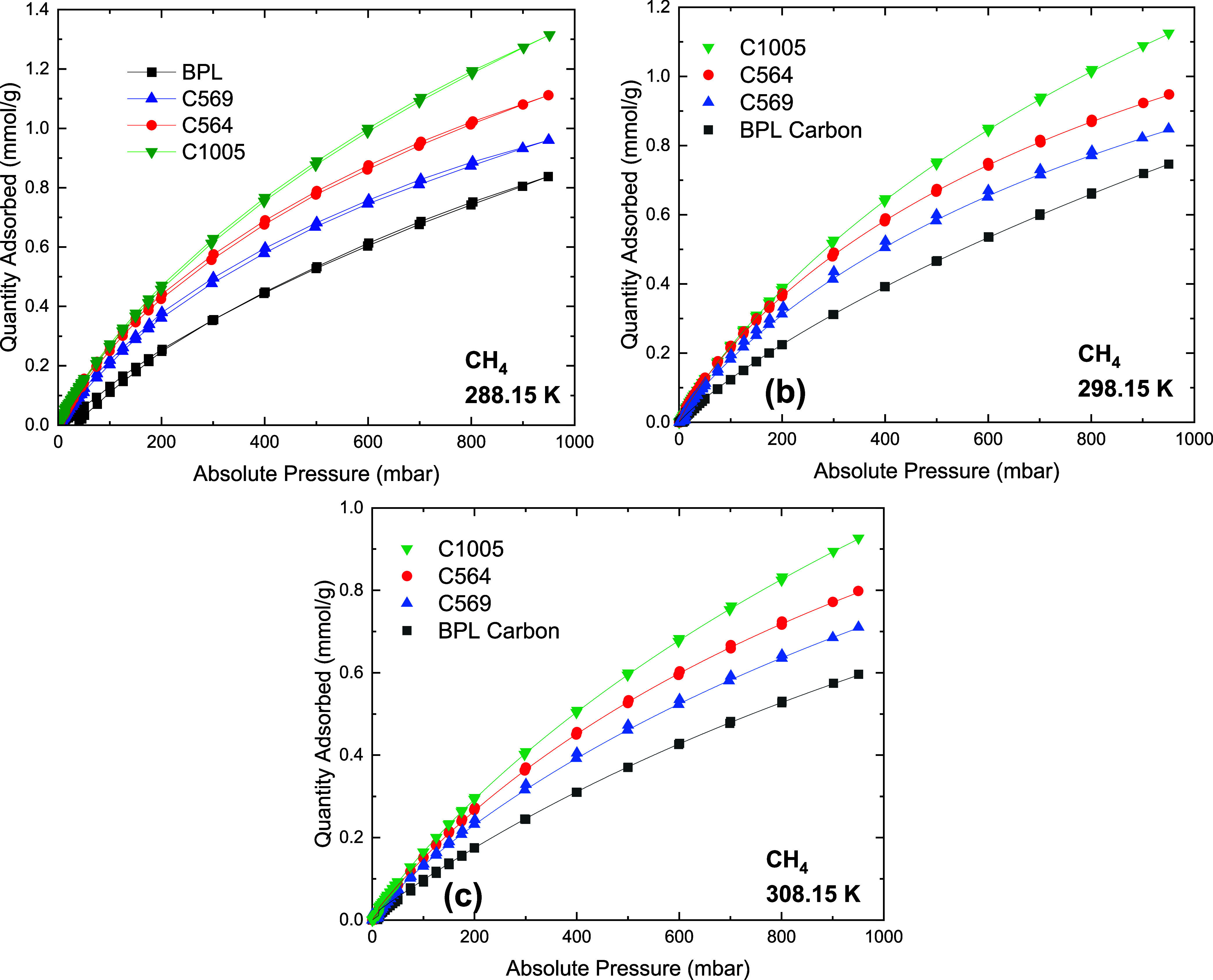
CH_4_ isotherms on carbon materials at (a) 288.15 K, (b) 298.15 K, and (c) 308.15 K. Closed symbols represent adsorption data, and lines represent DSLF model fitting.

**Figure 5:**
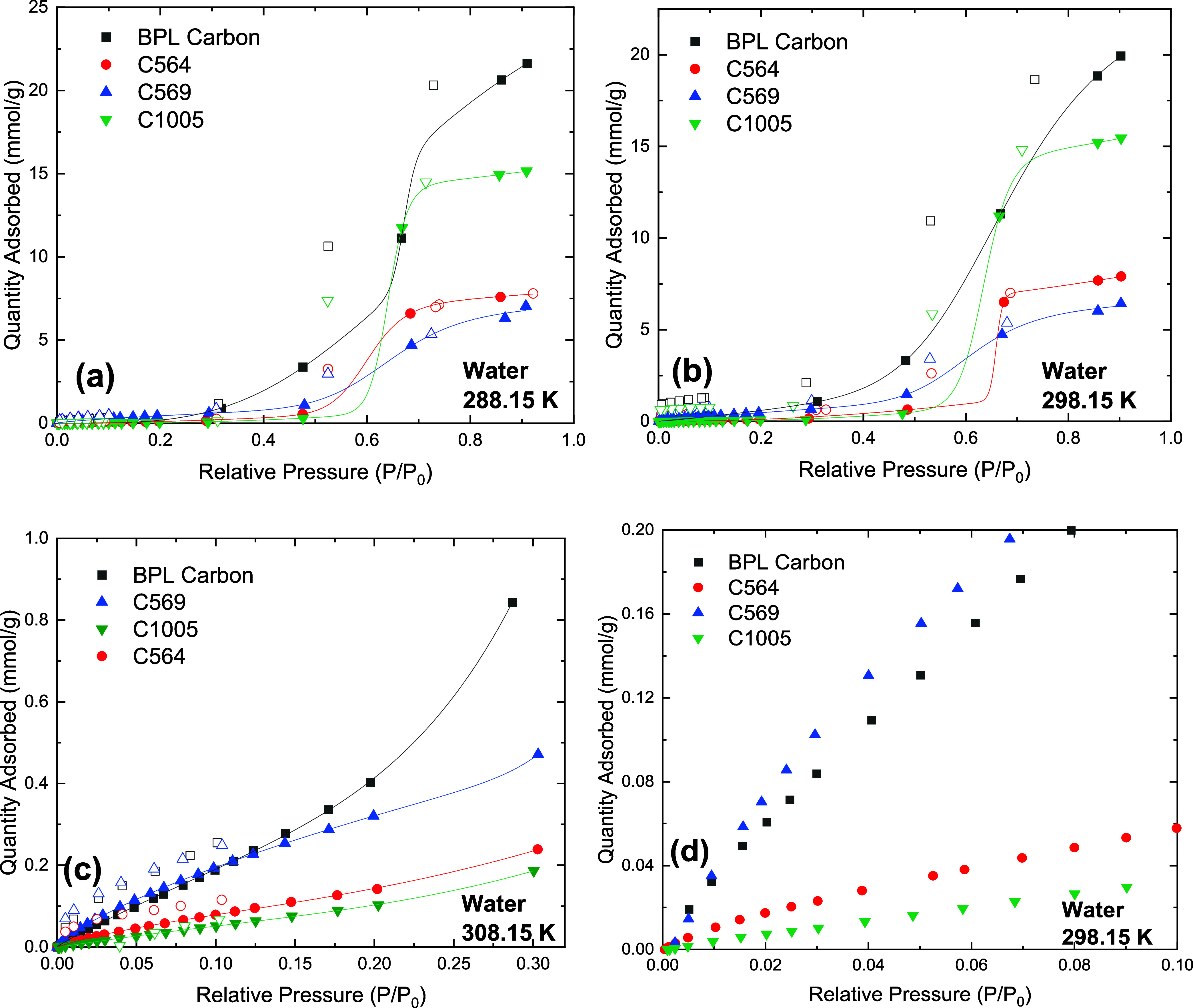
Water isotherms on carbon materials at (a) 288.15 K, (b) 298.15 K, (c) 308.15 K, and (d) *P*/*P*_0_ < 0.1, 298.15 K. Closed symbols represent adsorption data, open symbols represent desorption data, and lines represent DSLF model fitting.

**Figure 6:**
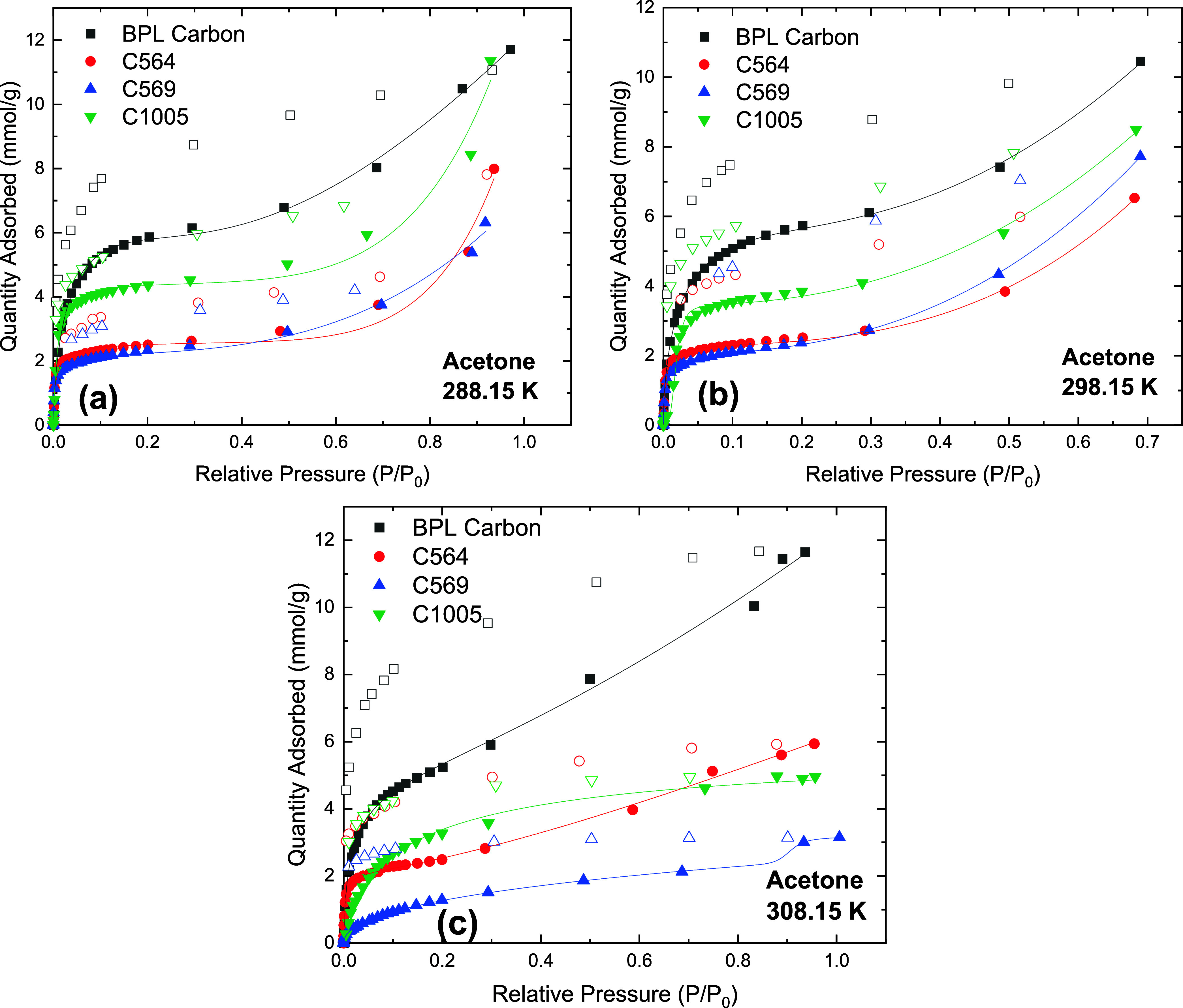
Acetone isotherms on carbon materials at (a) 288.15 K, (b) 298.15 K, (c) 308.15 K. Closed symbols represent adsorption data, open symbols represent desorption data, and lines represent DSLF model fitting.

The adsorption and desorption data of CO_2_, CH_4_, water, and acetone on the four carbon materials at 288.15, 298.15, and 308.15 K are shown in Figures 3–6 and tabulated in Tables S5–S8, respectively. The desorption branches coincide with the adsorption step, and no hysteresis is observed. Isotherms were also collected for CO_2_, CH_4_, and water vapor at 323.15 K as shown in Figures S9–S11 and Table S9–S11, respectively. The CO_2_ and CH_4_ isotherms in Figures 3 and 4, respectively, show that at pressures approaching 1000 mbar, generally, the adsorption loadings decrease in the order of C1005 > C564 > C569 > BPL carbon. The loadings of CO_2_ and CH_4_ on BPL carbon further agree with existing data in the literature as shown in Figures S12 and S13 using data from Delgado et al. and Álvarez-Gutiérrez et al., respectively.^34,35^

The higher loading of CO_2_ in the Carboxen materials may be attributed to the higher density of smaller-sized pores in the Carboxens compared to BPL carbon which favors CO_2_ adsorption. At low pressures <200 mbar, C1005 and C564 adsorb similar amounts of CO_2_ and CH_4_.

The water isotherms measured at 288 and 298 K are Type V according to the IUPAC classification (Figure 5). This shape is in agreement with the existing literature as shown in Figure S15 with data from Do et al.^36^ The Type V isotherm shape results from weak interactions between water vapor and carbon surfaces. The uptake takes place at higher relative humidity, and capillary condensation takes place at the relative pressures matching those of small mesopores. Among all samples, BPL had the highest adsorption capacity at all measured humidity values and the most pronounced hysteresis loops for the water condensation step. The latter agrees with this material having the highest amount of oxygen functional groups and the highest micropore volumes, in addition to small mesopores, respectively.^37^ The occurrence of these hysteresis loops (as shown clearly in Figures S14–S16) could be due to different water adsorption and desorption mechanisms within the material’s pores. It was observed that the hysteresis loops for the Carboxens are narrower than that of BPL carbon, with C564 and C569 being narrower than that of C1005. This trend agrees with the oxygen content in these materials, indicating that the higher the oxygen content, the broader the hysteresis loop. Hence, these loops may be attributed to the differences in surface chemistry of the Carboxens and BPL carbon as well as the smaller pore size distributions in these materials, especially those of C564 and C569 compared to C1005.^38,39^ It was observed that at 298 K and at 10% relative humidity, BPL carbon adsorbs >80% more water than C1005. On the other hand, C1005 exhibits the most hydrophobic behavior of all four materials at different temperatures, a consequence of its lowest oxygen content. At high relative humidity, C1005 has high water uptake given its high pore volume compared to those of C564 and C569. Water adsorption capacity at all temperatures decreases in the order of BPL carbon > C1005 > C564 > C569, which correlates to the total pore volumes of the adsorbents.

Acetone isotherms in Figure 6 show high adsorption loadings at low relative pressures *P*/*P*_0_ < 0.1 compared to water isotherms. These high loadings result from the stronger interaction forces between the adsorbate and the adsorbent vs the interaction between adsorbate molecules in the bulk phase, which is further verified by the broad desorption hysteresis for all materials. The adsorption loading is highest in BPL carbon, and the order correlates with the pore volumes of the materials and higher content of surface oxygen functional groups. The isotherm and loadings for BPL carbon are in good agreement with results reported by Barton et al. (Figure S18).^40^ The pore-filling mechanism dominates as the relative pressure increases with packing of molecules into the micropores. The adsorbed quantity at the same pressure points reduces slightly as the temperature increases, except for C569 at 308 K where a significant reduction in uptake is observed at lower partial pressures. Here, the hysteresis loops shown in Figures S17–S19 are different compared to those of the water isotherms. The width of the hysteresis loop does not follow the same trend with pore size distribution and is also present at very low pressures. This may be explained as a result of the strong interactions between the surface of the materials and acetone rather than the effect of pore size distribution and pore networks on the adsorption and desorption of acetone.^41^

### Isosteric Heat of Adsorption

The DSLF model provided a good correlation with the isotherms, particularly CO_2_ and CH_4_, and was used for fitting all isotherms for the heat of adsorption calculations. The shapes of the isotherms are indicative of the surface heterogeneity of these carbon materials, and the DSLF model correlation for the four molecules confirms the heterogeneity of the adsorbents’ surfaces.^42,43^ The fitting parameters derived from adsorption experiments on the carbon materials are listed in Table 4. However, for heat of adsorption calculations, only a few adsorption data sets at low pressures of *P*/*P*_0_ ≤ 0.5 were used to model the isotherms for more accurate results.

**Table 4:** DSLF Fitting Parameters for CO_2_, CH_4_, Water, and Acetone on Carbon Adsorbents at 288.15, 298.15, and 308.15 K

		288.15 K				298.15 K				308.15 K			
adsorbent		CH_4_	CO_2_	water	acetone	CH_4_	CO_2_	water	acetone	CH_4_	CO_2_	water	acetone
BPL carbon	*q*_1_	0.017	6.757	6.431	3.648	17.397	0.090	2.753	5.501	0.012	0.088	71.734	6.010
	*q*_2_	3.822	0.202	3.982	2.935	2.689	6.006	7.218	1.345	1.889	5.502	223.676	0.659
	*k*_1_	0.077	3.34 × 10^–04^	0.004	0.641	4.84 × 10^–05^	0.031	0.017	0.081	0.077	0.021	2.38 × 10^–04^	0.049
	*k*_2_	4.38 × 10^–04^	1.83 × 10^–02^	0.109	0.071	3.37 × 10^–04^	3.32 × 10^–04^	0.040	1.554	4.67 × 10^–04^	2.75 × 10^–04^	0.016	1.828
	*n*_1_	164.139	0.890	0.809	1.224	2.063	1.443	0.837	0.848	163.988	1.218	0.898	0.739
	*n*_2_	0.930	1.099	3.302	1.204	0.890	0.885	2.559	1.217	0.996	0.903	4.793	1.262
C564	*q*_1_	1.474	0.633	–1588.987	1.183	1.053	4.738	61.876	1.811	2.020	–2.98 × 10^–05^	–60.099	1.538
	*q*_2_	1.474	3.854	6343.236	1.768	1.053	–0.068	14.826	1.494	0.007	3.002	263.990	1.561
	*k*_1_	8.29 × 10^–04^	0.013	0.013	0.045	8.34 × 10^–04^	6.79 × 10^–04^	6.12 × 10^–05^	1.742	6.56 × 10^–04^	3.11 × 10^–12^	0.007	0.018
	*k*_2_	8.29 × 10^–04^	8.19 × 10^–04^	0.005	2.661	8.34 × 10^–04^	0.374	0.037	0.014	0.078	0.001	0.002	1.718
	*n*_1_	0.854	0.993	1.554	0.816	0.874	0.707	0.813	1.441	0.946	0.741	1.215	0.762
	*n*_2_	0.854	0.902	1.541	1.770	0.874	0.911	6.235	0.759	163.513	0.896	1.170	1.250
C569	*q*_1_	1.016	1.795	5.039	1.861	0.010	1.733	3.382	2.158	2.018	3.224	2.928	0.152
	*q*_2_	1.016	1.795	10.184	1.443	1.912	1.733	0.056	1.430	0.041	0.403	110.831	4.195
	*k*_1_	8.57 × 10^–04^	0.001	0.003	0.023	0.077	0.001	0.025	0.011	5.12 × 10^–04^	5.99 × 10^–04^	0.006	3.856
	*k*_2_	8.57 × 10^–04^	0.001	0.003	3.933	7.96 × 10^–04^	0.001	1.867	2.129	0.016	0.008	0.022	0.003
	*n*_1_	0.873	0.800	0.765	0.674	38.407	0.823	1.066	0.708	0.974	0.961	0.781	1.830
	*n*_2_	0.873	0.800	0.765	1.495	0.916	0.823	1.758	1.412	1.194	1.001	7.886	0.706
C1005	*q*_1_	3.468	0.481	–775.602	1.572	1.640	6.299	0.003	1.974	1.645	5.281	1835.337	0.214
	*q*_2_	0.131	7.067	3098.156	3.269	1.640	1.181	1.157	1.839	1.645	0.305	–456.285	4.112
	*k*_1_	5.34 × 10^–04^	0.012	0.015	0.064	5.01 × 10^–04^	5.30 × 10^–04^	0.036	0.096	3.66 × 10^–04^	4.54 × 10^–04^	0.001	0.226
	*k*_2_	0.010	4.85 × 10^–04^	0.006	1.027	5.01 × 10^–04^	111.240	0.052	0.227	3.66 × 10^–04^	0.009	0.004	0.031
	*n*_1_	0.952	1.008	1.644	0.911	0.879	0.826	–48.110	1.932	0.889	0.963	1.406	52.540
	*n*_2_	1.008	0.914	1.629	1.638	0.879	–204.130	2.364	195.097	0.889	1.003	1.423	1.021

The isosteric heats of adsorption of CO_2_, CH_4_, water, and acetone in BPL carbon, obtained from the Clausius–Clapeyron equation, agree well with the results reported in the literature as shown in Table 5. The heat of adsorption for water corresponds to the hydrophobicity of the materials, increasing in the following order: C569 > BPL carbon > C564 > C1005. The adsorption of water onto the material is typically driven by interactions between the polar water molecules and the polar sites on the surface. BPL carbon and C569 have higher surface energy due to their higher composition of polar functional groups as observed in the elemental analysis, which leads to stronger interactions with water molecules. The heats of adsorption calculated for CO_2_ and CH_4_ on these materials increase in the same order as the adsorption loadings at low pressures. With the exception of C564, *Q*_st_ values calculated for each molecule are slightly higher for CO_2_ than CH_4_. The higher *Q*_st_ is indicative of stronger adsorbate–adsorbent interactions. Moreover, the obtained *Q*_st_ values lie primarily in the physisorption range for all four materials.^39^

**Table 5:** Heats of Adsorption on Carbon Adsorbents for CO_2_, CH_4_, Water, and Acetone

	*Q*_st_ (kj/mol)					
adsorbate	BPL carbon	C564	C569	C1005	BPL carbon Lit.	ref
CO_2_	17.84	26.71	24.30	29.00	20.9	(35)
CH_4_	16.20	31.72	23.12	27.09	18.4	(35)
water	32.44	25.86	57.35	15.44	50.0	(44)
acetone	10.3	10.00	58.99	96.4	15.2	(41)

From the results gathered, we can make some inferences as to what VOC removal applications some of these materials may be best suited for. C1005 exhibited the most hydrophobicity with moderate VOC adsorption and could therefore be applied to VOC adsorption under humid conditions, specifically at a relative humidity below 50%. BPL carbon, on the other hand, adsorbed significant quantities of acetone at very low partial pressures compared to the Carboxens and also had the lowest uptake of CO_2_ and CH_4_ under the same conditions as the Carboxens. This implies that BPL carbon may be efficient in removing low concentration VOCs, particularly polar VOCs, from dry streams as well as streams with CO_2_ and CH_4_ present.

## Conclusions

Adsorption isotherms for CO_2_, CH_4_, water, and acetone were reported for specialty carbon molecular sieves with tapered pores, Carboxen 564, 569, and 1005, and then compared with that of BPL carbon. Similar adsorption behavior was observed for the molecules on the four materials. The CO_2_ adsorption loading on the Carboxens was slightly higher than that on BPL carbon due to the ultramicroporosity of these materials, and C1005 exhibited the highest uptake of CO_2_ and CH_4_ across different temperatures. The Carboxen materials were observed to be more hydrophobic than BPL carbon with C1005 being the most hydrophobic while BPL carbon depicted the strongest affinity for acetone at low pressures as a result of its higher oxygen content. The isosteric heats of adsorption were calculated after fitting the isotherms using the DSLF model. The heats of adsorption were in the same order as uptakes for CO_2_, CH_4_, and water.

For real-world applications, C1005 could be a viable material for removing polar and nonpolar VOCs from humid streams such as the analysis of VOC biomarkers in exhaled breath. On the other hand, BPL carbon can be applied for removing VOCs from streams with low relative humidity conditions and CO_2_ and CH_4_ such as dehydrated flue gas. C569 showed a higher affinity for CH_4_ at 288.15 K and can be studied further for increased methane uptake at low temperatures. It is important to note that the selection of the right type of carbon materials depends on several factors such as the specific VOCs that need to be removed, the operating conditions, and the desired level of removal efficiency. Therefore, more research is required to understand the behavior of nonpolar VOCs as well as multicomponent VOC adsorption on these carbon materials.

## Supporting Information Available

N_2_ physisorption data points and corresponding pore size and cumulative pore volume data for four samples. Isotherm data points for CO_2_ at 273 K and corresponding pore size and cumulative pore volume data. EDS maps showing the density of atoms present and XPS spectra for carbon samples. All CO_2_, CH_4_, water, and acetone adsorption data points measured for the four carbon samples at 288.15, 298.15, and 308.15 K. Graphs and isotherm data for all molecules on BPL carbon and Carboxens at 323.15 K. Hysteresis loops for CO_2_ and CH_4_ isotherms at 298 K. Hysteresis loops for water and acetone at all three temperatures (PDF)
Isotherms AIF file (ZIP)
