# Supplementary material for: Single-Component Adsorption Equilibria of CO2, CH4, Water, and Acetone on Tapered Porous Carbon Molecular Sieves
Source: J Chem Eng Data. 2024 Feb 22;69(3):1411–22. doi: 10.1021/acs.jced.3c00368 (PMC10945479; doi:10.1021/acs.jced.3c00368)
Supplement: Supplementary file 1 — je3c00368_si_001.pdf [file je3c00368_si_001.pdf]

Supplementary Information

Single Component Adsorption Equilibria of CO_2, CH_4, Water and Acetone on Tapered-Porous Carbon Molecular Sieves

Ojuolape O. Oghenetega,[1] Pasquale Fulvio,[1] N. Scott Bobbitt,[2] and Krista S. Walton[1*]

[1]School of Chemical & Biomolecular Engineering

Georgia Institute of Technology, Atlanta, GA 30332, United States

[2]Sandia National Laboratory, Albuquerque, NM 87185, United States

*Corresponding author. E-mail address: krista.walton@gatech.edu

Table S1. N_2 Physisorption data at 77 K on BPL Carbon, C564, C569, and C1005

BPL Carbon			C564			C569			C1005		
Relative Pressure (P/P_0)	n (cm^3/g STP)	Elapsed Time (h:min)	Relative Pressure (P/P_0)	n (cm^3/g STP)	Elapsed Time (h:min)	Relative Pressure (P/P_0)	n (cm^3/g STP)	Elapsed Time (h:min)	Relative Pressure (P/P_0)	n (cm^3/g STP)	Elapsed Time (h:min)
5.18E-07	0 ± 0	04:37	9.66E-04	115.4 ± 0.2	08:12	0.001	73.8 ± 0.1	06:28	4.87E-07	0 ± 0	04:57
2.00E-06	36.9 ± 0.1	09:41	0.001	117.8 ± 0.2	08:26	0.001	75.7 ± 0.1	06:48	1.01E-06	45.1 ± 0.1	10:38
5.45E-06	50.9 ± 0.1	10:08	0.002	123.8 ± 0.2	08:46	0.002	81.7 ± 0.1	07:53	4.96E-06	70.1 ± 0.1	11:44
1.26E-05	66.6 ± 0.1	11:05	0.005	130.6 ± 0.2	09:23	0.005	87.5 ± 0.1	09:16	1.14E-05	86.3 ± 0.2	13:08
1.65E-05	72.1 ± 0.1	11:58	0.007	133.9 ± 0.2	09:35	0.007	90.1 ± 0.1	09:51	1.62E-05	93.4 ± 0.2	14:17
3.16E-05	85.6 ± 0.2	12:36	0.010	136.5 ± 0.2	09:57	0.010	92.0 ± 0.1	10:09	3.12E-05	106.6 ± 0.2	15:06
6.24E-05	101.4 ± 0.2	12:59	0.026	144.3 ± 0.2	10:25	0.024	98.3 ± 0.2	11:01	6.21E-05	120.4 ± 0.2	16:00
1.24E-04	118.2 ± 0.2	13:36	0.053	149.5 ± 0.2	10:43	0.051	102.9 ± 0.2	11:30	1.25E-04	133.7 ± 0.3	17:03
2.47E-04	134.7 ± 0.3	14:01	0.076	152.1 ± 0.2	10:53	0.075	105.3 ± 0.2	11:41	2.44E-04	145.0 ± 0.3	18:20
5.05E-04	150.9 ± 0.3	14:20	0.102	154.2 ± 0.2	10:59	0.101	107.3 ± 0.2	11:53	4.89E-04	155.6 ± 0.3	19:40
0.001	169.2 ± 0.3	14:43	0.128	155.8 ± 0.3	11:02	0.128	108.8 ± 0.2	11:58	0.001	169.0 ± 0.3	21:13
0.004	188.7 ± 0.4	14:56	0.153	157.2 ± 0.3	11:05	0.154	110.0 ± 0.2	12:02	0.003	180.1 ± 0.3	22:18
0.005	194.0 ± 0.4	15:07	0.177	158.3 ± 0.3	11:07	0.177	111.0 ± 0.2	12:05	0.005	186.9 ± 0.4	23:17
0.008	201.5 ± 0.4	15:18	0.202	159.4 ± 0.3	11:09	0.202	112.0 ± 0.2	12:08	0.007	190.8 ± 0.4	23:53
0.009	205.8 ± 0.4	15:29	0.226	160.5 ± 0.3	11:11	0.226	112.9 ± 0.2	12:10	0.010	195.1 ± 0.4	24:19
0.010	206.8 ± 0.4	15:32	0.251	161.5 ± 0.3	11:13	0.251	113.7 ± 0.2	12:12	0.010	195.4 ± 0.4	24:28
0.021	224.3 ± 0.5	15:37	0.276	162.4 ± 0.3	11:15	0.275	114.5 ± 0.2	12:13	0.020	206.6 ± 0.4	24:39
0.030	233.6 ± 0.5	15:40	0.301	163.3 ± 0.3	11:17	0.300	115.4 ± 0.2	12:16	0.032	213.8 ± 0.4	24:53
0.043	243.2 ± 0.5	15:44	0.325	164.2 ± 0.3	11:18	0.325	116.1 ± 0.2	12:18	0.042	217.9 ± 0.4	25:06
0.051	248.8 ± 0.5	15:48	0.350	165.0 ± 0.3	11:20	0.350	116.9 ± 0.2	12:19	0.052	221.2 ± 0.4	25:19
0.115	275.1 ± 0.6	15:52	0.374	165.8 ± 0.3	11:22	0.374	117.6 ± 0.2	12:21	0.102	229.3 ± 0.4	25:32
0.152	284.2 ± 0.6	15:54	0.399	166.7 ± 0.3	11:24	0.399	118.3 ± 0.2	12:22	0.152	233.3 ± 0.4	25:43
0.205	293.3 ± 0.6	15:57	0.448	168.2 ± 0.3	11:25	0.448	119.6 ± 0.2	12:24	0.200	236.1 ± 0.5	25:54
0.251	298.8 ± 0.6	15:59	0.498	169.7 ± 0.3	11:27	0.498	12.01 ± 0.2	12:26	0.250	238.4 ± 0.5	26:05
0.302	303.4 ± 0.6	16:01	0.594	172.9 ± 0.3	11:29	0.595	123.7 ± 0.2	12:27	0.300	240.5 ± 0.5	26:16
0.351	307.1 ± 0.6	16:04	0.694	177.1 ± 0.3	11:31	0.694	127.3 ± 0.2	12:29	0.350	242.5 ± 0.5	26:27
0.398	310.3 ± 0.6	16:06	0.799	185.2 ± 0.3	11:34	0.801	134.3 ± 0.2	12:32	0.398	244.2 ± 0.5	26:38
0.449	313.3 ± 0.6	16:09	0.845	192.5 ± 0.3	11:37	0.846	140.5 ± 0.2	12:34	0.448	245.9 ± 0.5	26:49
0.499	316.1 ± 0.6	16:12	0.873	200.0 ± 0.3	11:40	0.873	146.9 ± 0.2	12:37	0.498	247.6 ± 0.5	27:00
0.548	318.7 ± 0.6	16:14	0.895	209.8 ± 0.3	11:44	0.896	155.6 ± 0.2	12:40	0.549	249.4 ± 0.5	27:11

0.599	321.4 ± 0.6	16:17	0.922	230.9 ± 0.4	11:50	0.923	174.9 ± 0.3	12:45	0.598	251.2 ± 0.5	27:22
0.648	323.9 ± 0.7	16:20	0.948	274.0 ± 0.4	11:58	0.948	214.1 ± 0.3	12:50	0.648	253.4 ± 0.5	27:32
0.698	326.5 ± 0.7	16:23	0.969	374.2 ± 0.6	12:13	0.973	305.3 ± 0.5	13:02	0.699	256.1 ± 0.5	27:43
0.749	329.0 ± 0.7	16:25	0.976	390.6 ± 0.6	12:20	0.979	319.4 ± 0.5	13:06	0.749	259.4 ± 0.5	27:53
0.800	331.4 ± 0.7	16:27	0.996	401.1 ± 0.6	12:35	0.995	322.6 ± 0.5	13:17	0.797	264.1 ± 0.5	28:10
0.850	333.7 ± 0.7	16:29	0.979	397.4 ± 0.6	12:37	0.997	329.6 ± 0.5	13:28	0.845	272.2 ± 0.5	28:29
0.900	336.5 ± 0.7	16:31	0.957	397.1 ± 0.6	12:38	0.987	322.8 ± 0.5	13:29	0.899	292.9 ± 0.6	29:01
0.949	341.3 ± 0.7	16:33	0.936	395.0 ± 0.6	12:41	0.962	322.4 ± 0.5	13:30	0.947	355.9 ± 0.7	29:26
0.987	350.0 ± 0.7	16:36	0.931	368.5 ± 0.6	12:49	0.941	319.1 ± 0.5	13:33	0.995	474.4 ± 0.9	29:49
0.953	345.5 ± 0.7	16:38	0.900	235.5 ± 0.4	13:12	0.931	273.1 ± 0.4	13:41	0.949	473.1 ± 0.9	30:01
0.903	341.2 ± 0.7	16:40	0.878	216.4 ± 0.3	13:18	0.897	173.7 ± 0.3	13:51	0.900	317.8 ± 0.6	30:27
0.850	338.6 ± 0.7	16:42	0.844	201.7 ± 0.3	13:23	0.881	162.9 ± 0.3	13:54	0.848	281.2 ± 0.5	30:35
0.801	336.7 ± 0.7	16:44	0.822	195.7 ± 0.3	13:26	0.845	148.9 ± 0.2	13:57	0.806	270.8 ± 0.5	30:46
0.750	335.0 ± 0.7	16:46	0.805	192.6 ± 0.3	13:28	0.821	143.6 ± 0.2	13:59	0.754	263.8 ± 0.5	30:57
0.700	333.4 ± 0.7	16:48	0.754	186.2 ± 0.3	13:31	0.806	141.3 ± 0.2	14:01	0.702	259.7 ± 0.5	31:06
0.651	331.7 ± 0.7	16:50	0.704	182.3 ± 0.3	13:33	0.753	135.8 ± 0.2	14:02	0.651	256.7 ± 0.5	31:17
0.601	329.9 ± 0.7	16:52	0.655	179.7 ± 0.3	13:34	0.704	132.8 ± 0.2	14:04	0.602	254.5 ± 0.5	31:28
0.551	327.9 ± 0.7	16:54	0.603	177.5 ± 0.3	13:36	0.654	130.6 ± 0.2	14:05	0.551	252.5 ± 0.5	31:40
0.500	325.8 ± 0.7	16:56	0.552	175.7 ± 0.3	13:38	0.603	128.8 ± 0.2	14:06	0.501	250.8 ± 0.5	31:52
0.455	317.6 ± 0.6	17:00	0.502	174.2 ± 0.3	13:39	0.552	127.4 ± 0.2	14:08	0.451	248.7 ± 0.5	32:03
0.400	311.3 ± 0.6	17:03	0.453	172.3 ± 0.3	13:41	0.502	126.1 ± 0.2	14:09	0.400	247.1 ± 0.5	32:13
0.351	307.7 ± 0.6	17:05	0.400	170.7 ± 0.3	13:42	0.452	124.5 ± 0.2	14:10	0.351	245.4 ± 0.5	32:23
0.302	304.0 ± 0.6	17:15	0.352	169.3 ± 0.3	13:44	0.400	123.2 ± 0.2	14:12	0.301	243.7 ± 0.5	32:33
0.251	299.3 ± 0.6	17:18	0.326	168.5 ± 0.3	13:45	0.352	122.0 ± 0.2	14:13	0.250	241.8 ± 0.5	32:44
0.201	293.4 ± 0.6	17:21				0.326	121.4 ± 0.2	14:14	0.201	239.8 ± 0.5	32:54
0.150	284.6 ± 0.6	17:26							0.151	237.3 ± 0.5	33:04
0.102	272.0 ± 0.5	17:29							0.101	233.5 ± 0.4	33:17
0.051	249.2 ± 0.5	17:35							0.051	225.4 ± 0.4	33:25
0.010	208.3 ± 0.4	17:51							0.011	201.4 ± 0.4	33:45
0.005	194.7 ± 0.4	18:02							0.010	201.7 ± 0.4	33:48
0.001	164.9 ± 0.3	19:12							0.005	192.0 ± 0.4	33:58
5.59E-04	152.7 ± 0.3	20:22							0.001	171.9 ± 0.3	35:25
1.02E-04	113.5 ± 0.2	33:50							5.59E-04	163.9 ± 0.3	37:10

Table S2. Pore size distribution with cumulative pore volumes for BPL Carbon, C564, C569, and C1005 from N_2 Isotherm data

BPL Carbon			C564			Carboxen 569			Carboxen 1005		
Pore Width	Cumulative Pore Volume	dV/dW Pore Volume	Pore Width	Cumulative Pore Volume	dV/dW Pore Volume	Pore Width	Cumulative Pore Volume	dV/dW Pore Volume	Pore Width	Cumulative Pore Volume	dV/dW Pore Volume
0.391	0.000	0.000	0.873	0.173	0.244	0.873	0.097	0.297	0.391	0.000	0.000
0.409	0.000	0.000	0.908	0.181	0.174	0.908	0.107	0.221	0.409	0.000	0.000
0.427	0.000	0.000	0.962	0.188	0.152	0.962	0.116	0.197	0.427	0.000	0.000
0.445	0.000	0.000	0.998	0.193	0.123	0.998	0.123	0.156	0.445	0.000	0.000
0.463	0.000	0.000	1.051	0.198	0.097	1.051	0.128	0.112	0.463	0.000	0.000
0.480	0.000	0.000	1.087	0.201	0.078	1.087	0.131	0.070	0.480	0.000	0.000
0.516	0.000	0.000	1.140	0.205	0.064	1.140	0.133	0.038	0.516	0.012	0.461
0.534	0.004	0.236	1.194	0.209	0.078	1.194	0.135	0.034	0.534	0.027	0.839
0.552	0.019	0.539	1.247	0.214	0.089	1.247	0.137	0.041	0.552	0.060	1.212
0.587	0.053	1.298	1.301	0.218	0.076	1.301	0.140	0.045	0.587	0.109	1.843
0.605	0.091	1.400	1.354	0.220	0.038	1.354	0.142	0.034	0.605	0.157	1.786
0.641	0.136	1.259	1.426	0.221	0.014	1.426	0.143	0.027	0.641	0.205	1.348
0.677	0.152	0.623	1.479	0.221	0.005	1.479	0.145	0.023	0.677	0.223	0.688
0.694	0.162	0.343	1.543	0.222	0.008	1.543	0.146	0.017	0.694	0.234	0.413
0.730	0.162	0.000	1.629	0.223	0.017	1.629	0.147	0.016	0.730	0.237	0.075
0.766	0.162	0.000	1.686	0.224	0.018	1.686	0.148	0.011	0.766	0.237	0.009
0.801	0.167	0.145	1.772	0.225	0.012	1.772	0.149	0.008	0.801	0.240	0.084
0.837	0.180	0.359	1.857	0.226	0.008	1.857	0.149	0.009	0.837	0.247	0.189
0.873	0.199	0.536	1.943	0.226	0.006	1.943	0.150	0.011	0.873	0.256	0.262
0.908	0.226	0.608	2.028	0.227	0.005	2.028	0.151	0.007	0.908	0.269	0.283
0.962	0.248	0.490	2.114	0.227	0.003	2.114	0.151	0.003	0.962	0.279	0.224
0.998	0.262	0.334	2.200	0.227	0.002	2.200	0.151	0.002	0.998	0.286	0.159
1.051	0.268	0.123	2.314	0.227	0.001	2.314	0.152	0.003	1.051	0.290	0.078
1.087	0.270	0.047	2.399	0.227	0.000	2.399	0.152	0.001	1.087	0.292	0.049
1.140	0.272	0.041	2.514	0.227	0.000	2.514	0.152	0.000	1.140	0.295	0.054
1.194	0.280	0.138	2.628	0.227	0.000	2.628	0.152	0.000	1.194	0.301	0.121
1.247	0.292	0.239	2.742	0.227	0.000	2.742	0.152	0.000	1.247	0.310	0.166
1.301	0.306	0.259	2.885	0.227	0.000	2.885	0.152	0.000	1.301	0.318	0.157
1.354	0.319	0.206	2.999	0.227	0.000	2.999	0.152	0.000	1.354	0.325	0.111
1.426	0.328	0.134	3.141	0.227	0.000	3.141	0.152	0.000	1.426	0.329	0.065
1.479	0.335	0.122	3.284	0.227	0.000	3.284	0.152	0.000	1.479	0.333	0.057
1.543	0.345	0.140	3.427	0.227	0.000	3.427	0.152	0.000	1.543	0.338	0.065
1.629	0.355	0.139	3.598	0.227	0.000	3.598	0.152	0.000	1.629	0.343	0.069
1.686	0.363	0.111	3.769	0.227	0.000	3.769	0.152	0.000	1.686	0.346	0.053
1.772	0.371	0.096	3.912	0.227	0.000	3.912	0.152	0.000	1.772	0.348	0.024
1.857	0.385	0.162	4.112	0.227	0.000	4.112	0.152	0.000	1.857	0.349	0.007
1.943	0.406	0.241	4.283	0.227	0.000	4.283	0.152	0.000	1.943	0.349	0.000
2.028	0.419	0.155	4.483	0.227	0.000	4.483	0.152	0.000	2.028	0.349	0.000
2.114	0.424	0.064	4.682	0.227	0.000	4.682	0.152	0.000	2.114	0.349	0.000
2.200	0.429	0.044	4.882	0.227	0.000	4.882	0.152	0.000	2.200	0.349	0.000
2.314	0.434	0.056	5.110	0.227	0.000	5.110	0.152	0.000	2.314	0.349	0.000
2.399	0.439	0.043	5.339	0.227	0.000	5.339	0.152	0.000	2.399	0.349	0.000
2.514	0.441	0.024	5.595	0.227	0.000	5.595	0.152	0.000	2.514	0.349	0.000
2.628	0.444	0.020	5.852	0.227	0.000	5.852	0.152	0.000	2.628	0.349	0.000
2.742	0.446	0.019	6.109	0.227	0.000	6.109	0.152	0.000	2.742	0.349	0.000
2.885	0.448	0.016	6.366	0.227	0.000	6.366	0.152	0.000	2.885	0.349	0.000
2.999	0.450	0.015	6.680	0.228	0.002	6.680	0.152	0.002	2.999	0.349	0.000
3.141	0.452	0.014	6.965	0.229	0.004	6.965	0.153	0.003	3.141	0.349	0.000
3.284	0.454	0.014	7.279	0.229	0.000	7.279	0.153	0.000	3.284	0.349	0.000
3.427	0.456	0.013	7.622	0.229	0.000	7.622	0.153	0.000	3.427	0.349	0.000
3.598	0.458	0.010	7.964	0.230	0.004	7.964	0.154	0.003	3.598	0.349	0.000
3.769	0.459	0.010	8.306	0.233	0.006	8.306	0.156	0.005	3.769	0.349	0.000

3.912	0.461	0.010	8.677	0.234	0.003	8.677	0.157	0.003	3.912	0.349	0.000
4.112	0.463	0.009	9.077	0.236	0.006	9.077	0.159	0.006	4.112	0.349	0.000
4.283	0.465	0.010	9.476	0.240	0.010	9.476	0.163	0.008	4.283	0.349	0.000
4.483	0.466	0.009	9.904	0.244	0.009	9.904	0.166	0.007	4.483	0.349	0.000
4.682	0.467	0.005	10.361	0.244	0.000	10.361	0.166	0.000	4.682	0.349	0.000
4.882	0.468	0.005	10.818	0.244	0.000	10.818	0.166	0.000	4.882	0.349	0.000
5.110	0.470	0.009	11.303	0.245	0.003	11.303	0.167	0.003	5.110	0.349	0.000
5.339	0.472	0.007	11.816	0.251	0.011	11.816	0.173	0.010	5.339	0.349	0.000
5.595	0.473	0.004	12.359	0.261	0.018	12.359	0.181	0.015	5.595	0.349	0.000
5.852	0.474	0.004	12.929	0.271	0.017	12.929	0.189	0.014	5.852	0.349	0.000
6.109	0.475	0.003	13.500	0.276	0.009	13.500	0.194	0.007	6.109	0.349	0.000
6.366	0.476	0.002	14.099	0.285	0.014	14.099	0.201	0.012	6.366	0.349	0.000
6.680	0.477	0.004	14.727	0.293	0.012	14.727	0.208	0.010	6.680	0.349	0.001
6.965	0.478	0.004	15.412	0.299	0.009	15.412	0.214	0.009	6.965	0.351	0.005
7.279	0.480	0.005	16.097	0.306	0.010	16.097	0.220	0.009	7.279	0.351	0.000
7.622	0.481	0.003	16.810	0.317	0.014	16.810	0.230	0.013	7.622	0.351	0.000
7.964	0.481	0.000	17.581	0.321	0.006	17.581	0.234	0.005	7.964	0.353	0.006
8.306	0.481	0.000	18.380	0.334	0.015	18.380	0.246	0.014	8.306	0.356	0.010
8.677	0.481	0.002	19.207	0.348	0.017	19.207	0.260	0.016	8.677	0.356	0.000
9.077	0.482	0.002	20.063	0.355	0.008	20.063	0.266	0.008	9.077	0.356	0.000
9.476	0.483	0.001	20.976	0.371	0.017	20.976	0.282	0.016	9.476	0.361	0.011
9.904	0.483	0.001	21.918	0.385	0.014	21.918	0.294	0.013	9.904	0.368	0.016
10.361	0.485	0.003	22.888	0.397	0.012	22.888	0.304	0.010	10.361	0.368	0.000
10.818	0.486	0.003	23.944	0.412	0.015	23.944	0.316	0.011	10.818	0.368	0.000
11.303	0.487	0.001	25.000	0.440	0.025	25.000	0.337	0.019	11.303	0.368	0.000
11.816	0.487	0.000	26.141	0.471	0.027	26.141	0.360	0.020	11.816	0.371	0.006
12.359	0.487	0.000	27.311	0.492	0.018	27.311	0.376	0.013	12.359	0.384	0.022
12.929	0.487	0.000	28.538	0.502	0.008	28.538	0.383	0.006	12.929	0.398	0.025
13.500	0.487	0.000	29.823	0.531	0.022	29.823	0.407	0.018	13.500	0.402	0.007
14.099	0.488	0.001	31.164	0.549	0.013	31.164	0.422	0.011	14.099	0.410	0.012
14.727	0.488	0.000	32.562	0.556	0.005	32.562	0.429	0.005	14.727	0.417	0.011
15.412	0.488	0.000	34.046	0.570	0.009	34.046	0.442	0.009	15.412	0.424	0.010
16.097	0.488	0.001	35.558	0.578	0.006	35.558	0.451	0.006	16.097	0.433	0.013
16.810	0.489	0.001	37.185	0.587	0.005	37.185	0.459	0.005	16.810	0.447	0.018
17.581	0.489	0.000	38.840	0.594	0.004	38.840	0.469	0.005	17.581	0.452	0.007
18.380	0.490	0.001	40.609	0.596	0.001	40.609	0.472	0.002	18.380	0.466	0.017
19.207	0.491	0.001	42.436	0.597	0.001	42.436	0.478	0.003	19.207	0.482	0.019
20.063	0.492	0.001	44.319	0.598	0.001	44.319	0.482	0.002	20.063	0.490	0.009
20.976	0.493	0.001	46.316	0.599	0.000	46.316	0.483	0.001	20.976	0.507	0.018
21.918	0.494	0.001	48.400	0.599	0.000	48.400	0.483	0.000	21.918	0.521	0.014
22.888	0.495	0.001	50.597	0.606	0.003	50.597	0.483	0.000	22.888	0.530	0.009
23.944	0.496	0.001							23.944	0.539	0.009
25.000	0.497	0.001							25.000	0.553	0.013
26.141	0.498	0.001							26.141	0.567	0.012
27.311	0.499	0.001							27.311	0.576	0.008
28.538	0.499	0.000							28.538	0.581	0.004
29.823	0.500	0.001							29.823	0.594	0.010
31.164	0.501	0.001							31.164	0.603	0.007
32.562	0.502	0.000							32.562	0.607	0.003
34.046	0.502	0.001							34.046	0.617	0.006
35.558	0.503	0.000							35.558	0.623	0.004
37.185	0.504	0.000							37.185	0.630	0.004
38.840	0.504	0.000							38.840	0.639	0.005
40.609	0.505	0.000							40.609	0.643	0.002
42.436	0.505	0.000							42.436	0.650	0.004
44.319	0.506	0.000							44.319	0.656	0.003
46.316	0.506	0.000							46.316	0.661	0.002
48.400	0.506	0.000							48.400	0.661	0.000
50.597	0.508	0.001							50.597	0.694	0.015

Table S3. CO_2 Physisorption data at 273 K on BPL Carbon, C564, C569, and C1005

BPL Carbon			C564			C569			C1005		
Relative Pressure (P/P_0)	n (cm^3/g STP)	Elapsed Time (h:min)	Relative Pressure (P/P_0)	n (cm^3/g STP)	Elapsed Time (h:min)	Relative Pressure (P/P_0)	n (cm^3/g STP)	Elapsed Time (h:min)	Relative Pressure (P/P_0)	n (cm^3/g STP)	Elapsed Time (h:min)
2.97E-05	0 ± 0	02:43	3.18E-05	0 ± 0	03:13	5.13E-06	0 ± 0	02:40	1.67E-05	0 ± 0	02:24
6.06E-05	0.02 ± 0	02:49	5.96E-05	0 ± 0	03:20	8.43E-06	0 ± 0	02:57	3.19E-05	0 ± 0	02:35
1.24E-04	0.04 ± 0	02:55	1.25E-04	0.1 ± 0	03:26	1.52E-05	0 ± 0	03:16	6.00E-05	0 ± 0	02:41
2.46E-04	0.09 ± 0	03:00	2.43E-04	0.1 ± 0	03:32	3.11E-05	0.1 ± 0	03:27	1.24E-04	0.1 ± 0	02:47
4.94E-04	0.19 ± 0	03:06	4.9E-04	0.3 ± 0	03:38	6.17E-05	0.1 ± 0	03:42	2.44E-04	0.1 ± 0	02:52
0.001	0.38 ± 0	03:12	0.001	0.6 ± 0	03:45	1.24E-04	0.2 ± 0	04:00	4.92E-04	0.2 ± 0	02:58
0.002	0.69 ± 0	03:18	0.002	1.2 ± 0	03:53	2.41E-04	0.3 ± 0	04:19	0.001	0.5 ± 0	03:04
0.004	1.34 ± 0	03:24	0.004	2.3 ± 0	04:00	4.8E-04	0.5 ± 0	04:39	0.002	0.9 ± 0	03:10
0.006	1.88 ± 0	03:29	0.006	3.3 ± 0.01	04:06	0.001	0.8 ± 0	04:57	0.004	1.8 ± 0	03:17
0.008	2.40 ± 0.01	03:33	0.008	4.2 ± 0.01	04:11	0.002	1.3 ± 0	05:11	0.006	2.6 ± 0	03:23
0.010	2.73 ± 0.01	03:41	0.010	4.9 ± 0.01	04:15	0.004	2.3 ± 0	05:23	0.008	3.4 ± 0	03:28
0.011	2.91 ± 0.01	03:47	0.011	5.3 ± 0.01	04:20	0.006	3.0 ± 0	05:32	0.010	4.2 ± 0	03:32
0.012	3.12 ± 0.01	03:51	0.012	5.7 ± 0.01	04:25	0.008	3.8 ± 0	05:40	0.011	4.5 ± 0	03:39
0.013	3.39 ± 0.01	03:55	0.013	6.2 ± 0.01	04:30	0.010	4.4 ± 0	05:46	0.012	4.8 ± 0	03:44
0.015	3.74 ± 0.01	03:59	0.015	6.8 ± 0.01	04:33	0.011	4.8 ± 0	05:51	0.013	5.2 ± 0	03:49
0.017	4.25 ± 0.01	04:06	0.017	7.6 ± 0.02	04:38	0.012	5.1 ± 0	05:56	0.015	5.7 ± 0	03:53
0.020	4.77 ± 0.01	04:10	0.020	8.4 ± 0.02	04:41	0.013	5.5 ± 0	06:01	0.017	6.3 ± 0	03:58
0.022	5.26 ± 0.01	04:15	0.022	9.2 ± 0.02	04:46	0.015	6.0 ± 0	06:06	0.019	7.0 ± 0	04:02
0.025	5.61 ± 0.01	04:20	0.025	10.0 ± 0.02	04:49	0.017	6.7 ± 0	06:11	0.022	7.7 ± 0	04:07
0.027	5.92 ± 0.01	04:24	0.027	10.7 ± 0.02	04:53	0.020	7.5 ± 0	06:15	0.025	8.4 ± 0	04:12
0.030	6.46 ± 0.01	04:29	0.030	11.4 ± 0.02	04:57	0.022	8.1 ± 0	06:20	0.027	9.0 ± 0	04:17
0.034	7.16 ± 0.02	04:33	0.034	12.6 ± 0.03	05:02	0.025	8.7 ± 0	06:25	0.030	9.7 ± 0	04:22
0.039	8.00 ± 0.02	04:38	0.039	13.8 ± 0.03	05:06	0.027	9.3 ± 0	06:29	0.034	10.7 ± 0	04:26
0.044	8.89 ± 0.02	04:42	0.044	15.0 ± 0.03	05:10	0.030	10.0 ± 0	06:34	0.039	11.9 ± 0	04:31
0.050	9.53 ± 0.02	04:46	0.049	16.0 ± 0.03	05:13	0.034	11.0 ± 0	06:38	0.044	13.0 ± 0	04:35
0.073	12.42 ± 0.03	04:51	0.072	20.2 ± 0.04	05:18	0.039	12.0 ± 0	06:43	0.049	14.1 ± 0	04:39
0.099	15.33 ± 0.03	04:55	0.099	24.3 ± 0.05	05:21	0.045	13.1 ± 0	06:47	0.072	18.2 ± 0	04:44
0.123	17.73 ± 0.04	04:59	0.123	27.4 ± 0.06	05:25	0.050	14.1 ± 0	06:52	0.098	22.5 ± 0	04:48
0.148	19.91 ± 0.04	05:04	0.148	30.3 ± 0.06	05:29	0.072	17.8 ± 0	06:56	0.123	26.0 ± 0.1	04:52
0.172	22.02 ± 0.05	05:08	0.172	32.7 ± 0.07	05:32	0.099	21.3 ± 0	07:01	0.148	29.3 ± 0.1	04:57

0.198	24.13 ± 0.05	05:12	0.197	35.0 ± 0.07	05:36	0.123	24.1 ± 0	07:05	0.172	32.3 ± 0.1	05:01
0.292	30.84 ± 0.07	05:17	0.292	42.1 ± 0.09	05:39	0.147	26.6 ± 0.1	07:09	0.197	34.9 ± 0.1	05:05
0.394	37.15 ± 0.08	05:21	0.394	47.9 ± 0.10	05:43	0.172	28.8 ± 0.1	07:14	0.290	43.8 ± 0.1	05:10
0.492	42.57 ± 0.09	05:25	0.492	52.4 ± 0.11	05:46	0.197	30.9 ± 0.1	07:18	0.394	52.0 ± 0.1	05:14
0.591	47.51 ± 0.10	05:29	0.591	56.2 ± 0.12	05:51	0.292	37.0 ± 0.1	07:23	0.492	58.7 ± 0.1	05:19
0.690	52.09 ± 0.11	05:32	0.691	59.6 ± 0.13	05:56	0.394	42.2 ± 0.1	07:27	0.591	64.7 ± 0.1	05:23
0.789	56.38 ± 0.12	05:36	0.790	62.6 ± 0.13	06:01	0.492	46.1 ± 0.1	07:32	0.690	70.1 ± 0.1	05:26
0.887	60.34 ± 0.13	05:39	0.889	65.2 ± 0.14	06:06	0.591	49.6 ± 0.1	07:36	0.788	75.0 ± 0.1	05:30
0.936	62.31 ± 0.14	05:43	0.938	66.5 ± 0.14	06:11	0.690	52.5 ± 0.1	07:41	0.887	79.5 ± 0.2	05:34
0.794	56.88 ± 0.13	05:47	0.791	63.1 ± 0.13	06:17	0.786	55.0 ± 0.1	07:46	0.936	81.7 ± 0.2	05:37
0.694	52.66 ± 0.12	05:51	0.692	60.3 ± 0.13	06:22	0.887	57.4 ± 0.1	07:51	0.795	75.7 ± 0.1	05:41
0.592	48.02 ± 0.11	05:55	0.592	57.1 ± 0.12	06:27	0.937	58.6 ± 0.1	07:57	0.696	70.9 ± 0.1	05:45
0.495	43.17 ± 0.10	06:00	0.492	53.3 ± 0.11	06:31	0.791	55.7 ± 0.1	08:02	0.593	65.3 ± 0.1	05:50
0.396	37.66 ± 0.08	06:04	0.395	48.9 ± 0.10	06:36	0.692	53.4 ± 0.1	08:07	0.495	59.5 ± 0.1	05:54
0.297	31.61 ± 0.07	06:09	0.297	43.4 ± 0.09	06:40	0.592	50.7 ± 0.1	08:12	0.396	52.8 ± 0.1	05:58
0.198	24.55 ± 0.05	06:13	0.199	36.2 ± 0.08	06:45	0.493	47.4 ± 0.1	08:17	0.298	45.1 ± 0.1	06:03
0.173	22.49 ± 0.05	06:18	0.173	33.8 ± 0.07	06:49	0.395	43.5 ± 0.1	08:21	0.199	35.9 ± 0.1	06:07
0.149	20.34 ± 0.04	06:22	0.148	31.4 ± 0.07	06:54	0.297	38.6 ± 0.1	08:26	0.173	32.9 ± 0.1	06:12
0.124	18.05 ± 0.04	06:27	0.124	28.7 ± 0.06	06:58	0.199	32.2 ± 0.1	08:30	0.148	29.9 ± 0.1	06:16
0.099	15.53 ± 0.03	06:31	0.099	25.5 ± 0.05	07:03	0.174	30.3 ± 0.1	08:36	0.124	26.8 ± 0.1	06:20
0.074	12.81 ± 0.03	06:36	0.075	21.8 ± 0.05	07:07	0.148	28.0 ± 0.1	08:40	0.099	23.3 ± 0	06:25
0.050	9.76 ± 0.02	06:41	0.050	17.4 ± 0.04	07:12	0.124	25.5 ± 0	08:45	0.075	19.4 ± 0	06:29
0.045	8.92 ± 0.02	06:45	0.045	16.2 ± 0.03	07:16	0.099	22.7 ± 0	08:49	0.050	14.9 ± 0	06:34
0.040	8.12 ± 0.02	06:49	0.040	15.0 ± 0.03	07:21	0.075	19.4 ± 0	08:54	0.045	13.6 ± 0	06:39
0.035	7.43 ± 0.02	06:54	0.035	13.8 ± 0.03	07:25	0.051	15.5 ± 0	08:59	0.040	12.5 ± 0	06:43
0.030	6.56 ± 0.01	06:58	0.030	12.7 ± 0.03	07:30	0.044	14.3 ± 0	09:05	0.035	11.5 ± 0	06:48
0.025	5.66 ± 0.01	07:03	0.025	11.2 ± 0.02	07:34	0.040	13.4 ± 0	09:09	0.030	10.2 ± 0	06:52
0.020	4.77 ± 0.01	07:08	0.020	9.7 ± 0.02	07:40	0.035	12.4 ± 0	09:14	0.025	8.9 ± 0	06:57
0.018	4.32 ± 0.01	07:13	0.018	8.8 ± 0.02	07:45	0.030	11.3 ± 0	09:19	0.020	7.6 ± 0	07:01
0.015	3.78 ± 0.01	07:19	0.015	7.9 ± 0.02	07:51	0.025	10.0 ± 0	09:25	0.018	6.8 ± 0	07:07
0.014	3.59 ± 0.01	07:24	0.014	7.4 ± 0.02	07:56	0.020	8.6 ± 0	09:33	0.015	6.0 ± 0	07:13
0.012	3.21 ± 0.01	07:30	0.012	6.8 ± 0.01	08:01	0.018	7.9 ± 0	09:38	0.014	5.7 ± 0	07:17
0.011	2.93 ± 0.01	07:34	0.011	6.2 ± 0.01	08:05	0.015	7.1 ± 0	09:46	0.012	5.2 ± 0	07:22
0.010	2.69 ± 0.01	07:39	0.010	5.7 ± 0.01	08:11	0.014	6.6 ± 0	09:53	0.011	4.8 ± 0	07:28
0.008	2.23 ± 0	07:43	0.008	4.9 ± 0.01	08:17	0.012	6.0 ± 0	10:00	0.010	4.3 ± 0	07:34
0.006	1.71 ± 0	07:49	0.006	4.0 ± 0.01	08:29	0.011	5.6 ± 0	10:08	0.008	3.7 ± 0	07:41

0.004	1.13 ± 0	08:05	0.004	2.8 ± 0.01	08:56	0.010	5.3 ± 0	10:15	0.006	2.9 ± 0	07:57
0.002	0.43 ± 0	08:56	0.002	1.6 ± 0	10:03	0.008	4.6 ± 0	10:25	0.004	1.9 ± 0	08:26
0.001	0 ± 0	10:33	0.001	0.8 ± 0	12:57	0.006	3.7 ± 0	10:46	0.002	1.0 ± 0	09:37
			0.001	0.3 ± 0	18:34	0.004	2.7 ± 0	11:24	0.001	0.4 ± 0	12:01
						0.002	1.7 ± 0	12:57	0.001	0 ± 0	16:47
						0.001	1.0 ± 0	15:45			
						4.8E-04	0.5 ± 0	20:48			

Table S4. Pore size distribution with cumulative pore volumes for BPL Carbon, C564, C569, and C1005 from CO_2 Isotherm data

BPL Carbon			C564			Carboxen 569			Carboxen 1005		
Pore Width	Cumulative Pore Volume	dV/dW Pore Volume	Pore Width	Cumulative Pore Volume	dV/dW Pore Volume	Pore Width	Cumulative Pore Volume	dV/dW Pore Volume	Pore Width	Cumulative Pore Volume	dV/dW Pore Volume
0.320	0.005	0.513	0.320	0.009	0.869	0.320	0.010	0.960	0.320	0.000	0.000
0.330	0.005	0.000	0.330	0.009	0.000	0.330	0.010	0.000	0.330	0.000	0.000
0.340	0.005	0.000	0.340	0.009	0.000	0.340	0.010	0.000	0.340	0.008	0.805
0.350	0.005	0.000	0.350	0.009	0.000	0.350	0.010	0.000	0.350	0.008	0.000
0.360	0.005	0.000	0.360	0.009	0.000	0.360	0.010	0.000	0.360	0.008	0.000
0.370	0.005	0.000	0.370	0.009	0.000	0.370	0.010	0.000	0.370	0.008	0.000
0.380	0.005	0.000	0.380	0.009	0.000	0.380	0.010	0.000	0.380	0.008	0.000
0.390	0.005	0.000	0.390	0.009	0.000	0.390	0.010	0.000	0.390	0.008	0.000
0.400	0.005	0.000	0.400	0.009	0.000	0.400	0.010	0.000	0.400	0.008	0.000
0.410	0.005	0.000	0.410	0.009	0.000	0.410	0.010	0.000	0.410	0.011	0.313
0.420	0.006	0.075	0.420	0.020	1.161	0.420	0.011	0.148	0.420	0.015	0.418
0.430	0.010	0.421	0.430	0.038	1.771	0.430	0.018	0.692	0.430	0.021	0.562
0.440	0.017	0.691	0.440	0.049	1.067	0.440	0.029	1.116	0.440	0.029	0.804
0.450	0.025	0.768	0.450	0.049	0.000	0.450	0.041	1.138	0.450	0.038	0.878
0.460	0.030	0.571	0.460	0.049	0.000	0.460	0.049	0.811	0.460	0.042	0.424
0.470	0.033	0.242	0.470	0.049	0.000	0.470	0.053	0.396	0.470	0.042	0.020
0.480	0.033	0.000	0.480	0.050	0.081	0.480	0.054	0.119	0.480	0.042	0.000
0.490	0.033	0.000	0.490	0.053	0.387	0.490	0.054	0.000	0.490	0.044	0.215
0.500	0.034	0.090	0.500	0.061	0.721	0.500	0.054	0.000	0.500	0.051	0.624
0.510	0.036	0.226	0.510	0.070	0.940	0.510	0.054	0.000	0.510	0.061	1.020
0.520	0.040	0.386	0.520	0.080	0.953	0.520	0.058	0.398	0.520	0.072	1.154
0.530	0.045	0.535	0.530	0.087	0.757	0.530	0.066	0.804	0.530	0.082	0.957
0.540	0.052	0.632	0.540	0.092	0.510	0.540	0.076	0.997	0.540	0.089	0.715
0.550	0.058	0.666	0.550	0.095	0.313	0.550	0.085	0.929	0.550	0.094	0.540
0.560	0.065	0.649	0.560	0.098	0.222	0.560	0.092	0.686	0.560	0.099	0.409
0.570	0.071	0.596	0.570	0.100	0.272	0.570	0.096	0.434	0.570	0.102	0.379
0.580	0.076	0.518	0.580	0.104	0.414	0.580	0.099	0.260	0.580	0.107	0.451
0.590	0.080	0.416	0.590	0.109	0.510	0.590	0.101	0.187	0.590	0.112	0.516
0.600	0.083	0.304	0.600	0.114	0.481	0.600	0.102	0.171	0.600	0.118	0.558
0.610	0.085	0.196	0.610	0.118	0.322	0.610	0.104	0.171	0.610	0.122	0.477
0.620	0.086	0.141	0.620	0.120	0.208	0.620	0.106	0.157	0.620	0.126	0.392
0.630	0.088	0.140	0.630	0.121	0.172	0.630	0.107	0.091	0.630	0.130	0.347
0.640	0.089	0.169	0.640	0.123	0.131	0.640	0.107	0.000	0.640	0.132	0.269
0.650	0.091	0.204	0.650	0.123	0.045	0.650	0.107	0.000	0.650	0.134	0.141
0.660	0.094	0.237	0.660	0.123	0.000	0.660	0.108	0.115	0.660	0.135	0.103
0.670	0.096	0.259	0.670	0.123	0.017	0.670	0.110	0.209	0.670	0.136	0.155
0.680	0.099	0.263	0.680	0.124	0.073	0.680	0.112	0.238	0.680	0.139	0.269
0.690	0.102	0.255	0.690	0.125	0.125	0.690	0.114	0.219	0.690	0.143	0.378
0.700	0.104	0.250	0.700	0.127	0.201	0.700	0.116	0.189	0.700	0.148	0.474
0.710	0.107	0.250	0.710	0.130	0.244	0.710	0.118	0.165	0.710	0.153	0.491
0.720	0.109	0.259	0.720	0.132	0.271	0.720	0.120	0.160	0.720	0.157	0.455
0.730	0.112	0.273	0.730	0.135	0.262	0.730	0.121	0.168	0.730	0.161	0.374
0.740	0.115	0.294	0.740	0.137	0.231	0.740	0.123	0.173	0.740	0.164	0.291
0.750	0.118	0.323	0.750	0.139	0.181	0.750	0.125	0.162	0.750	0.166	0.236
0.760	0.122	0.360	0.760	0.140	0.120	0.760	0.126	0.133	0.760	0.168	0.228
0.770	0.126	0.402	0.770	0.141	0.062	0.770	0.127	0.091	0.770	0.171	0.281
0.780	0.130	0.439	0.780	0.141	0.000	0.780	0.127	0.037	0.780	0.175	0.365

0.790	0.135	0.472	0.790	0.141	0.000	0.790	0.127	0.000	0.790	0.180	0.466
0.800	0.140	0.497	0.800	0.142	0.058	0.800	0.127	0.006	0.800	0.185	0.553
0.810	0.145	0.512	0.810	0.143	0.151	0.810	0.128	0.038	0.810	0.191	0.619
0.820	0.150	0.511	0.820	0.146	0.249	0.820	0.128	0.082	0.820	0.198	0.646
0.830	0.155	0.491	0.830	0.149	0.333	0.830	0.130	0.126	0.830	0.204	0.643
0.840	0.159	0.445	0.840	0.153	0.363	0.840	0.131	0.158	0.840	0.210	0.593
0.850	0.163	0.372	0.850	0.156	0.331	0.850	0.133	0.171	0.850	0.215	0.490
0.860	0.166	0.277	0.860	0.158	0.251	0.860	0.135	0.164	0.860	0.219	0.356
0.320	0.005	0.513	0.320	0.009	0.869	0.320	0.010	0.960	0.320	0.000	0.000
0.330	0.005	0.000	0.330	0.009	0.000	0.330	0.010	0.000	0.330	0.000	0.000
0.340	0.005	0.000	0.340	0.009	0.000	0.340	0.010	0.000	0.340	0.008	0.805
0.350	0.005	0.000	0.350	0.009	0.000	0.350	0.010	0.000	0.350	0.008	0.000
0.360	0.005	0.000	0.360	0.009	0.000	0.360	0.010	0.000	0.360	0.008	0.000
0.370	0.005	0.000	0.370	0.009	0.000	0.370	0.010	0.000	0.370	0.008	0.000
0.380	0.005	0.000	0.380	0.009	0.000	0.380	0.010	0.000	0.380	0.008	0.000
0.390	0.005	0.000	0.390	0.009	0.000	0.390	0.010	0.000	0.390	0.008	0.000
0.400	0.005	0.000	0.400	0.009	0.000	0.400	0.010	0.000	0.400	0.008	0.000
0.410	0.005	0.000	0.410	0.009	0.000	0.410	0.010	0.000	0.410	0.011	0.313
0.420	0.006	0.075	0.420	0.020	1.161	0.420	0.011	0.148	0.420	0.015	0.418
0.430	0.010	0.421	0.430	0.038	1.771	0.430	0.018	0.692	0.430	0.021	0.562
0.440	0.017	0.691	0.440	0.049	1.067	0.440	0.029	1.116	0.440	0.029	0.804
0.450	0.025	0.768	0.450	0.049	0.000	0.450	0.041	1.138	0.450	0.038	0.878
0.460	0.030	0.571	0.460	0.049	0.000	0.460	0.049	0.811	0.460	0.042	0.424
0.470	0.033	0.242	0.470	0.049	0.000	0.470	0.053	0.396	0.470	0.042	0.020
0.480	0.033	0.000	0.480	0.050	0.081	0.480	0.054	0.119	0.480	0.042	0.000
0.490	0.033	0.000	0.490	0.053	0.387	0.490	0.054	0.000	0.490	0.044	0.215
0.500	0.034	0.090	0.500	0.061	0.721	0.500	0.054	0.000	0.500	0.051	0.624
0.510	0.036	0.226	0.510	0.070	0.940	0.510	0.054	0.000	0.510	0.061	1.020
0.520	0.040	0.386	0.520	0.080	0.953	0.520	0.058	0.398	0.520	0.072	1.154
0.530	0.045	0.535	0.530	0.087	0.757	0.530	0.066	0.804	0.530	0.082	0.957
0.540	0.052	0.632	0.540	0.092	0.510	0.540	0.076	0.997	0.540	0.089	0.715
0.550	0.058	0.666	0.550	0.095	0.313	0.550	0.085	0.929	0.550	0.094	0.540
0.560	0.065	0.649	0.560	0.098	0.222	0.560	0.092	0.686	0.560	0.099	0.409
0.570	0.071	0.596	0.570	0.100	0.272	0.570	0.096	0.434	0.570	0.102	0.379
0.580	0.076	0.518	0.580	0.104	0.414	0.580	0.099	0.260	0.580	0.107	0.451
0.590	0.080	0.416	0.590	0.109	0.510	0.590	0.101	0.187	0.590	0.112	0.516
0.600	0.083	0.304	0.600	0.114	0.481	0.600	0.102	0.171	0.600	0.118	0.558
0.610	0.085	0.196	0.610	0.118	0.322	0.610	0.104	0.171	0.610	0.122	0.477
0.620	0.086	0.141	0.620	0.120	0.208	0.620	0.106	0.157	0.620	0.126	0.392
0.630	0.088	0.140	0.630	0.121	0.172	0.630	0.107	0.091	0.630	0.130	0.347
0.640	0.089	0.169	0.640	0.123	0.131	0.640	0.107	0.000	0.640	0.132	0.269
0.650	0.091	0.204	0.650	0.123	0.045	0.650	0.107	0.000	0.650	0.134	0.141
0.660	0.094	0.237	0.660	0.123	0.000	0.660	0.108	0.115	0.660	0.135	0.103
0.670	0.096	0.259	0.670	0.123	0.017	0.670	0.110	0.209	0.670	0.136	0.155
0.680	0.099	0.263	0.680	0.124	0.073	0.680	0.112	0.238	0.680	0.139	0.269
0.690	0.102	0.255	0.690	0.125	0.125	0.690	0.114	0.219	0.690	0.143	0.378
0.700	0.104	0.250	0.700	0.127	0.201	0.700	0.116	0.189	0.700	0.148	0.474
0.710	0.107	0.250	0.710	0.130	0.244	0.710	0.118	0.165	0.710	0.153	0.491
0.720	0.109	0.259	0.720	0.132	0.271	0.720	0.120	0.160	0.720	0.157	0.455
0.730	0.112	0.273	0.730	0.135	0.262	0.730	0.121	0.168	0.730	0.161	0.374
0.740	0.115	0.294	0.740	0.137	0.231	0.740	0.123	0.173	0.740	0.164	0.291
0.750	0.118	0.323	0.750	0.139	0.181	0.750	0.125	0.162	0.750	0.166	0.236
0.760	0.122	0.360	0.760	0.140	0.120	0.760	0.126	0.133	0.760	0.168	0.228
0.770	0.126	0.402	0.770	0.141	0.062	0.770	0.127	0.091	0.770	0.171	0.281
0.780	0.130	0.439	0.780	0.141	0.000	0.780	0.127	0.037	0.780	0.175	0.365
0.790	0.135	0.472	0.790	0.141	0.000	0.790	0.127	0.000	0.790	0.180	0.466

0.800	0.140	0.497	0.800	0.142	0.058	0.800	0.127	0.006	0.800	0.185	0.553
0.810	0.145	0.512	0.810	0.143	0.151	0.810	0.128	0.038	0.810	0.191	0.619
0.820	0.150	0.511	0.820	0.146	0.249	0.820	0.128	0.082	0.820	0.198	0.646
0.830	0.155	0.491	0.830	0.149	0.333	0.830	0.130	0.126	0.830	0.204	0.643
0.840	0.159	0.445	0.840	0.153	0.363	0.840	0.131	0.158	0.840	0.210	0.593
0.850	0.163	0.372	0.850	0.156	0.331	0.850	0.133	0.171	0.850	0.215	0.490
0.860	0.166	0.277	0.860	0.158	0.251	0.860	0.135	0.164	0.860	0.219	0.356

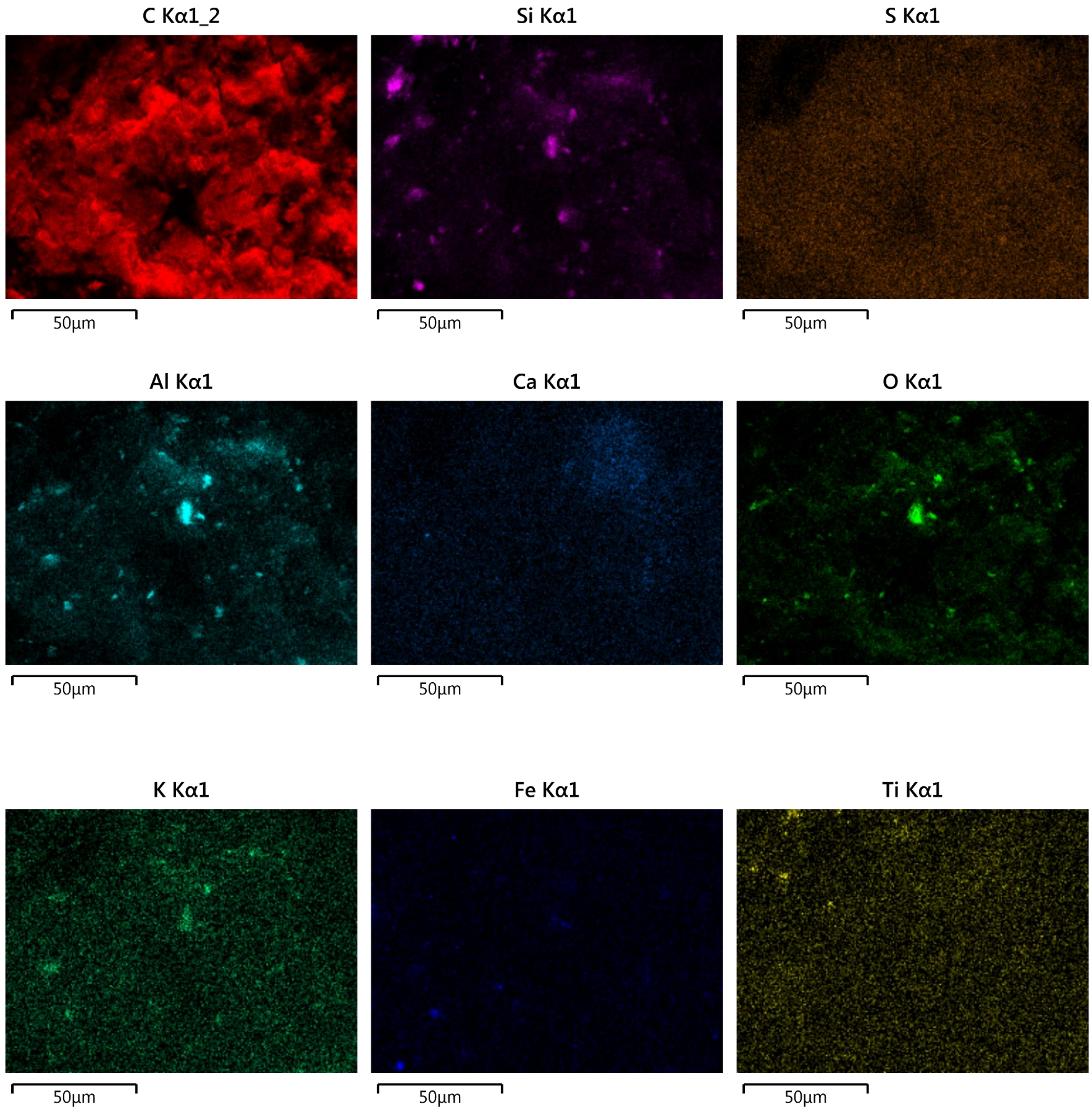

Figure S1. BPL Carbon EDS Map

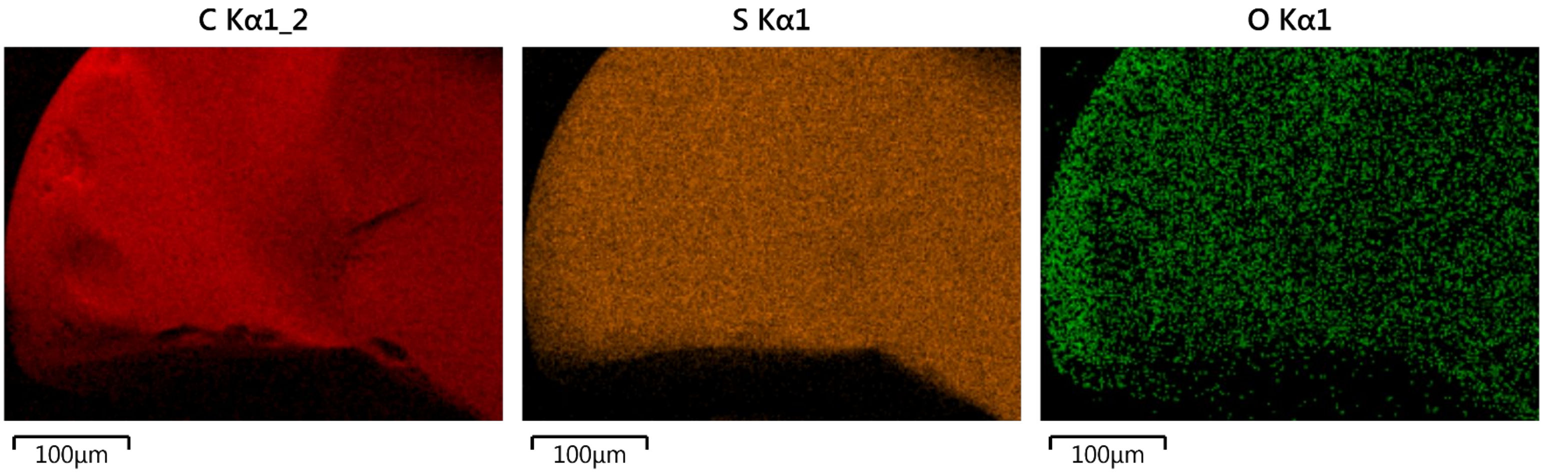

Figure S2. Carboxen 564 EDS Map

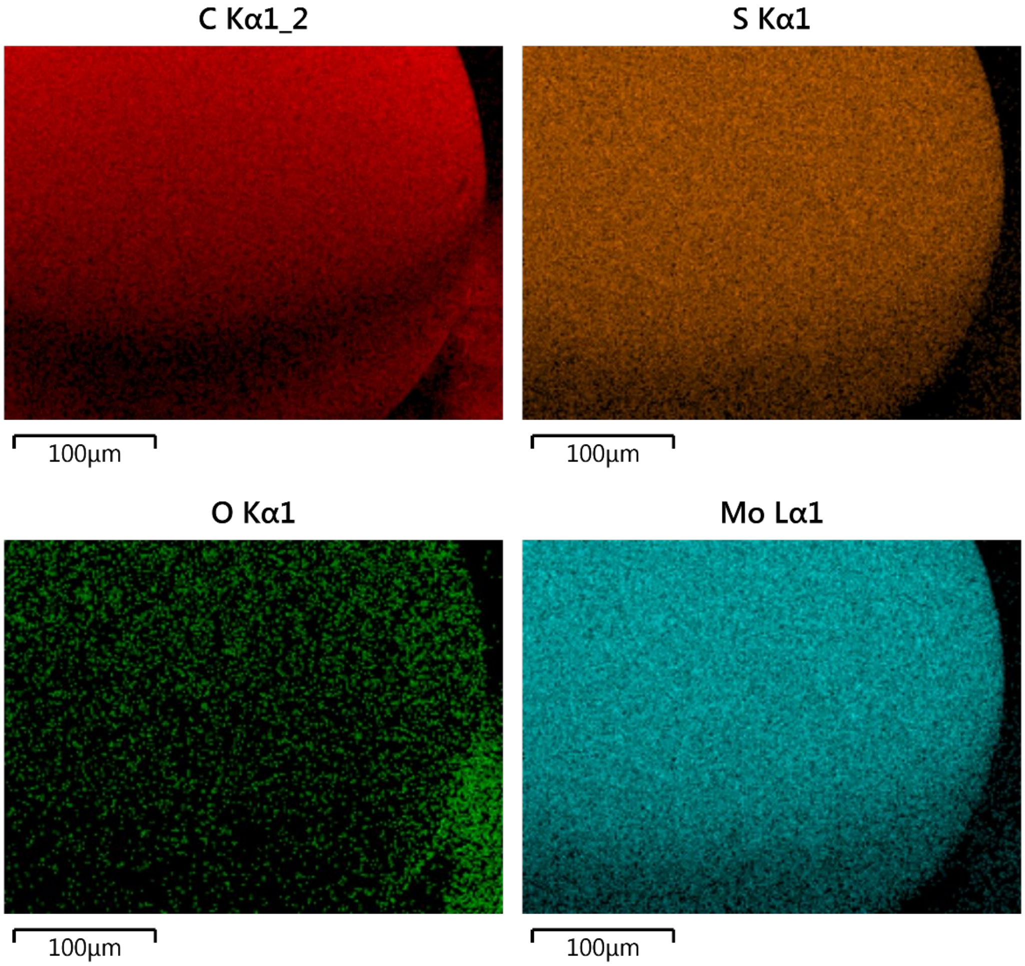

Figure S3. Carboxen 569 EDS Map

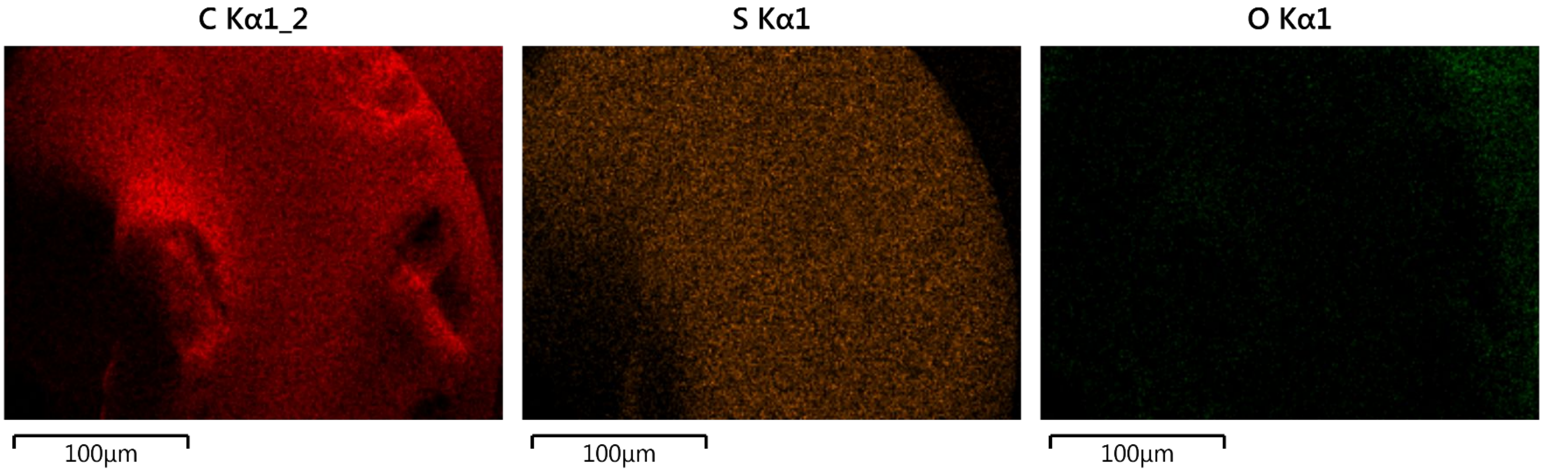

Figure S4. Carboxen 1005 EDS Map

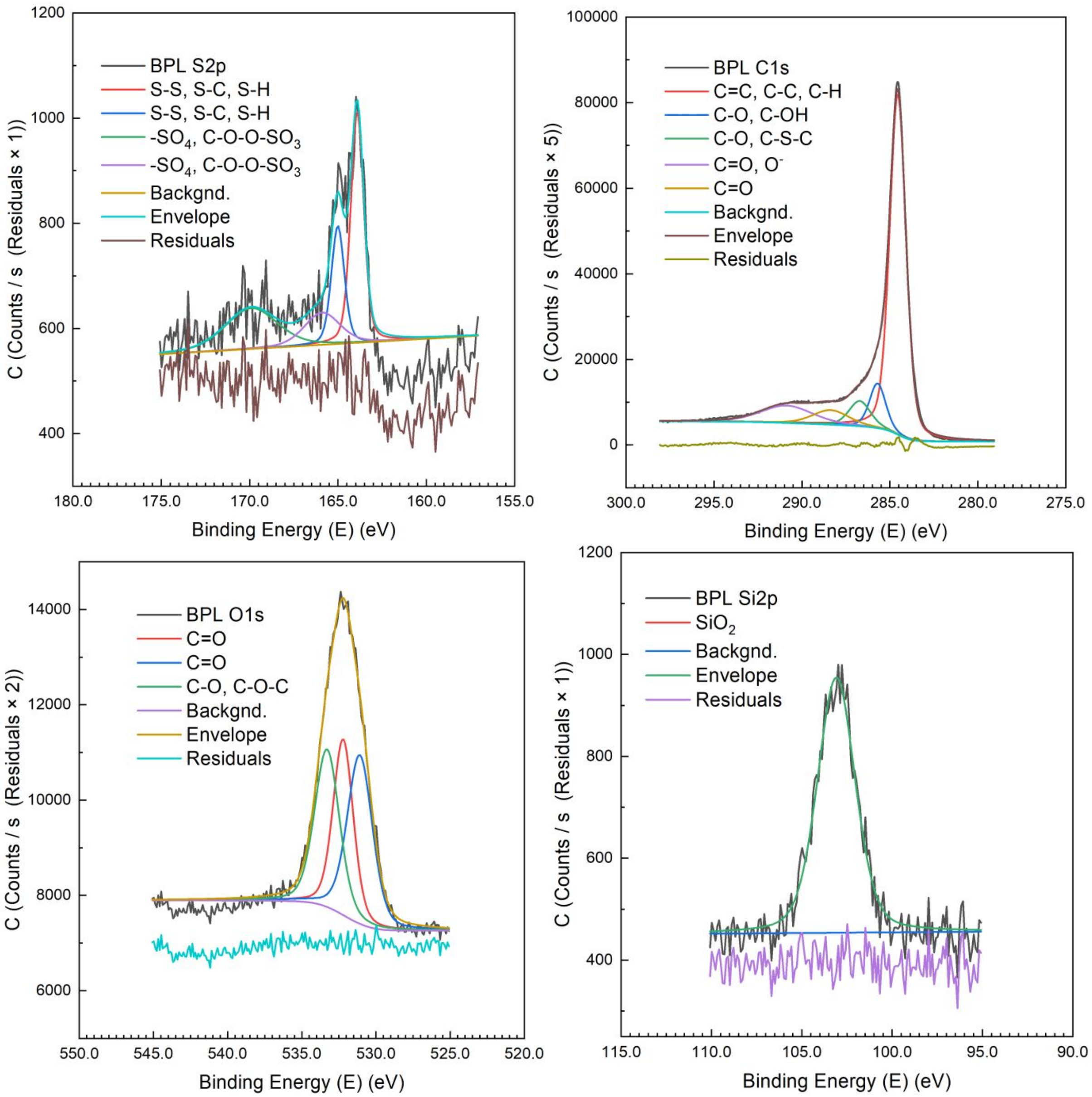

Figure S5. BPL Carbon XPS spectra

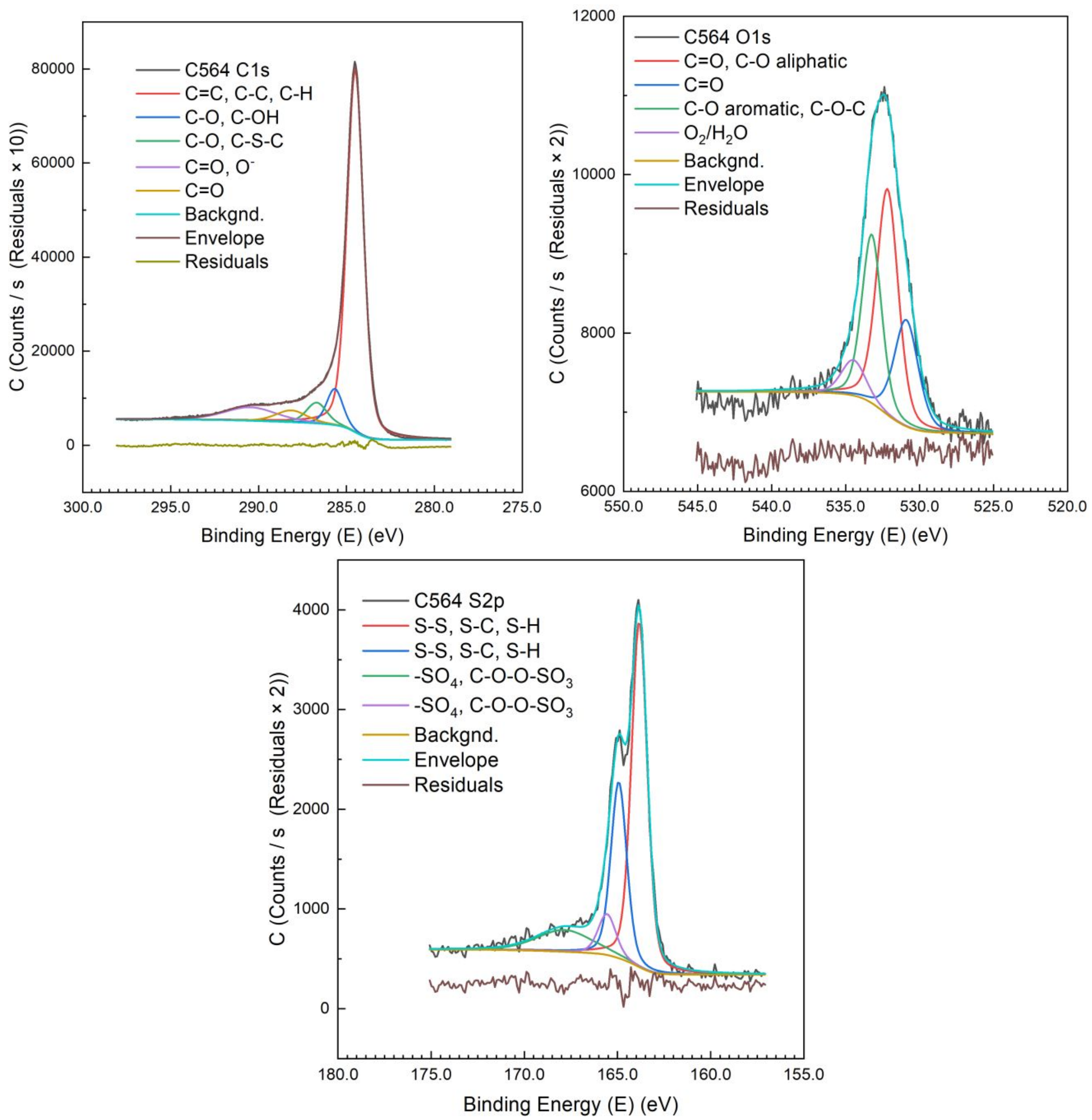

Figure S6. C564 Carbon XPS spectra

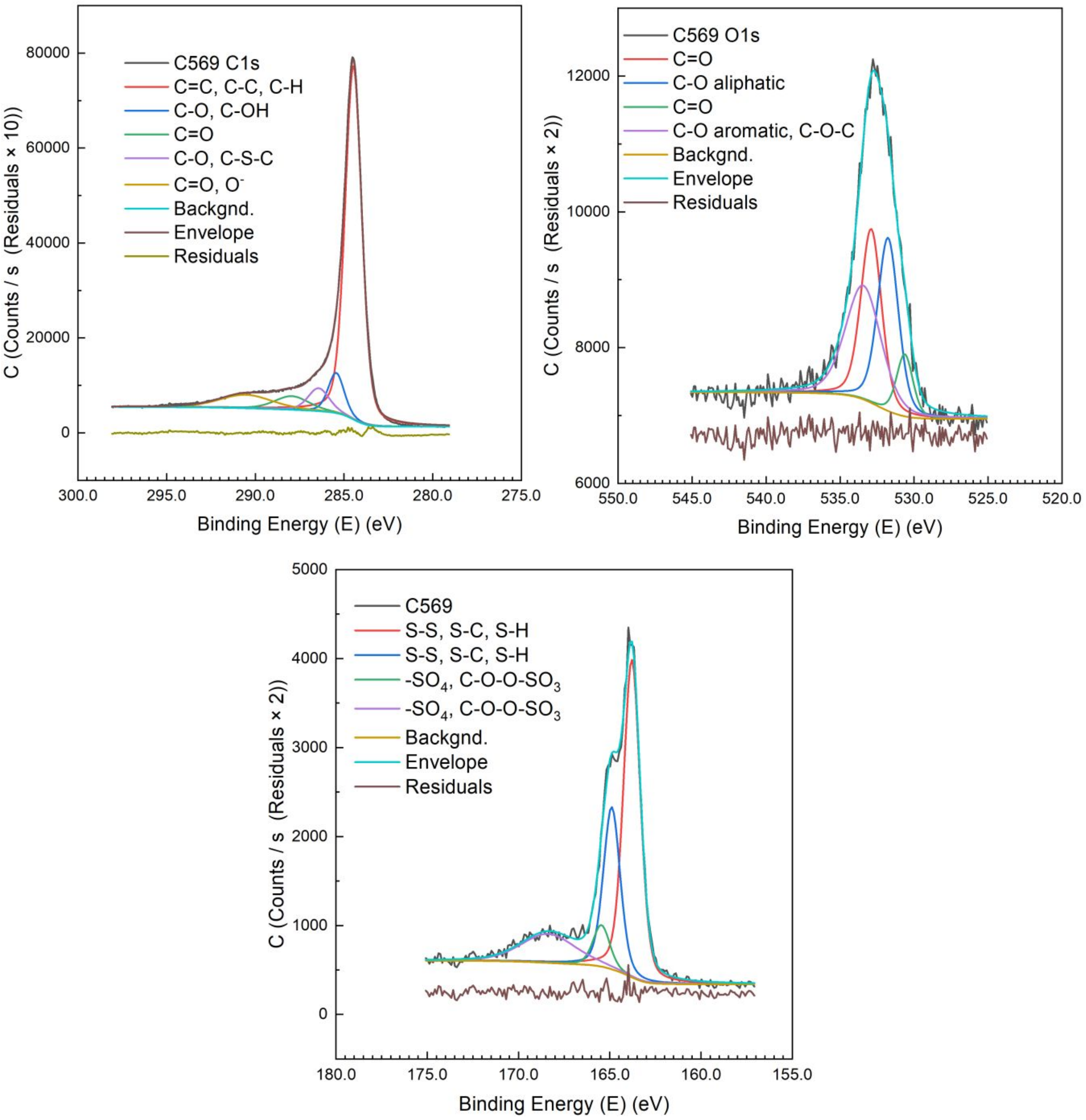

Figure S7. C569 Carbon XPS spectra

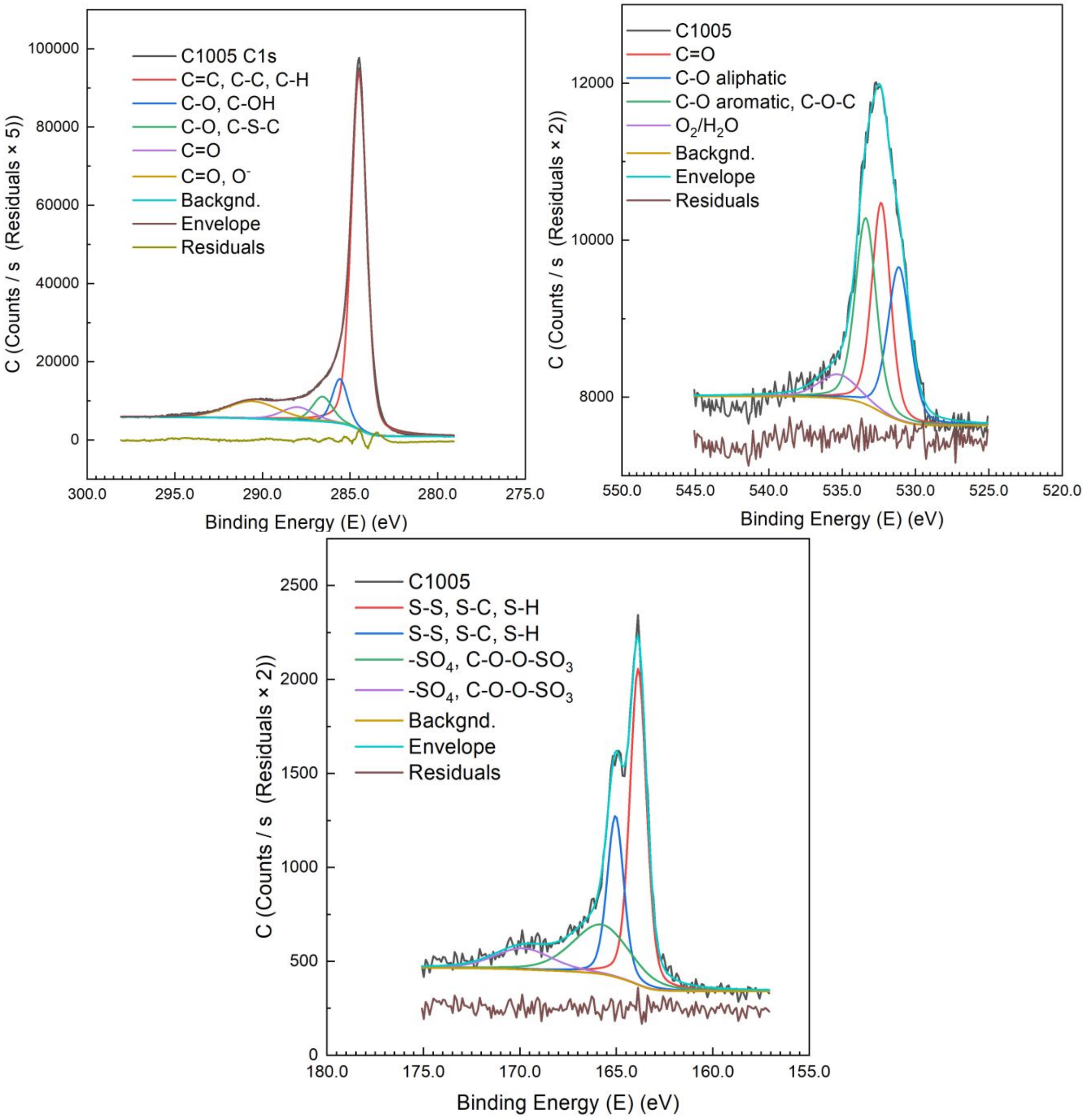

Figure S8. C1005 Carbon XPS spectra

Table S5. CH_4 adsorption data on carbon materials at (a) 288.15 K, (b) 298.15 K and (c) 308.15 K

	BPL Carbon			C564			C569			C1005		
	Absolute Pressure (mbar)	n (mmol/g)	Elapsed Time (h:min)	Absolute Pressure (mbar)	n (mmol/g)	Elapsed Time (h:min)	Absolute Pressure (mbar)	n (mmol/g)	Elapsed Time (h:min)	Absolute Pressure (mbar)	n (mmol/g)	Elapsed Time (h:min)
288.15 K	0.248	1.22 ± 0E-04	02:56	0.122	6.8 ± 0E-0.5	02:31	0.489	0.001 ± 0	02:37	0.122	5.6± 0E-05	02:25
	0.494	0.001 ± 0	03:00	0.244	4.4 ± 0E-0.4	02:36	0.976	0.002 ± 0	02:42	0.247	4.3± 0E-04	02:30
	0.995	0.001 ± 0	03:04	0.490	0.001 ± 0	02:40	1.952	0.005 ± 0	02:47	0.488	0.001 ± 0	02:34
	1.994	0.003 ± 0	03:08	0.975	0.003 ± 0	02:44	3.919	0.010 ± 0	02:52	0.980	0.003 ± 0	02:38
	3.950	0.007 ± 0	03:12	1.954	0.006 ± 0	02:49	5.959	0.016 ± 0	02:58	1.964	0.006 ± 0	02:42
	5.967	0.011 ± 0	03:17	3.919	0.013 ± 0	02:54	7.970	0.021 ± 0	03:03	3.911	0.013 ± 0	02:47
	7.973	0.014 ± 0	03:21	5.979	0.020 ± 0	02:59	10.139	0.024 ± 0	03:07	5.966	0.019 ± 0	02:51
	10.194	0.015 ± 0	03:24	7.985	0.026 ± 0	03:04	10.919	0.026 ± 0	03:11	7.962	0.026 ± 0	02:55
	11.013	0.016 ± 0	03:29	10.153	0.031 ± 0	03:08	11.986	0.027 ± 0	03:16	10.152	0.030 ± 0	02:59
	12.238	0.016 ± 0	03:33	10.929	0.033 ± 0	03:12	13.517	0.031 ± 0	03:21	10.920	0.033 ± 0	03:04
	13.595	0.019 ± 0	03:37	12.129	0.036 ± 0	03:18	15.113	0.035 ± 0	03:25	12.178	0.035 ± 0	03:09
	15.271	0.022 ± 0	03:41	13.580	0.041 ± 0	03:22	17.583	0.041 ± 0	03:28	13.154	0.054 ± 0	03:14
	17.558	0.024 ± 0	03:46	15.139	0.045 ± 0	03:26	20.064	0.048 ± 0	03:33	15.230	0.059 ± 0	03:19
	19.827	0.025 ± 0	03:50	17.559	0.052 ± 0	03:29	22.587	0.054 ± 0	03:38	17.429	0.065 ± 0	03:25
	22.349	0.030 ± 0	03:55	20.144	0.061 ± 0	03:35	25.020	0.062 ± 0	03:44	20.116	0.071 ± 0	03:30
	24.985	0.034 ± 0	03:59	22.476	0.067 ± 0	03:40	27.318	0.065 ± 0	03:49	22.399	0.076 ± 0	03:35
	27.396	0.033 ± 0	04:04	25.053	0.075 ± 0	03:45	29.834	0.071 ± 0	03:55	24.964	0.084 ± 0	03:40
	30.295	0.039 ± 0	04:08	27.552	0.087 ± 0	03:51	34.798	0.081 ± 0	04:01	27.482	0.091 ± 0	03:45
	34.862	0.046 ± 0	04:12	30.000	0.089 ± 0	03:57	39.919	0.092 ± 0	04:06	29.844	0.098 ± 0	03:50
	39.848	0.053 ± 0	04:17	34.769	0.108 ± 0	04:03	45.041	0.103 ± 0	04:11	34.622	0.110 ± 0	03:55
	44.760	0.060 ± 0	04:21	39.960	0.122 ± 0	04:08	49.807	0.111 ± 0	35:31	39.916	0.124 ± 0	04:00
	50.337	0.064 ± 0	04:26	45.084	0.128 ± 0	35:28	74.456	0.160 ± 0	35:37	44.991	0.137 ± 0	04:05
	74.201	0.094 ± 0	04:30	49.772	0.140 ± 0	35:33	99.871	0.204 ± 0	35:42	49.929	0.149 ± 0	04:09
	99.796	0.131 ± 0	04:34	74.140	0.197 ± 0	35:39	124.534	0.249 ± 0	35:47	73.846	0.207 ± 0	04:14
	124.863	0.166 ± 0	04:39	99.748	0.250 ± 0	35:44	149.806	0.289 ± 0.001	35:52	99.618	0.265 ± 0.001	04:19
	149.919	0.195 ± 0	04:43	124.872	0.301 ± 0.001	35:49	174.552	0.325 ± 0.001	35:57	124.484	0.317 ± 0.001	04:24
	174.561	0.224 ± 0	04:47	149.964	0.346 ± 0.001	35:54	199.708	0.361 ± 0.001	36:03	149.794	0.367 ± 0.001	04:29
	201.322	0.256 ± 0.001	04:52	174.799	0.387 ± 0.001	35:59	297.541	0.477 ± 0.001	36:08	174.377	0.412 ± 0.001	04:34
	298.312	0.353 ± 0.001	04:56	199.345	0.425 ± 0.001	36:04	398.808	0.579 ± 0.001	36:13	199.353	0.457 ± 0.001	04:38
	398.333	0.444 ± 0.001	05:00	296.904	0.557 ± 0.001	36:10	497.405	0.668 ± 0.001	36:18	296.790	0.612 ± 0.001	04:41
	499.044	0.528 ± 0.001	05:05	399.178	0.676 ± 0.001	36:15	599.202	0.746 ± 0.001	36:23	398.844	0.754 ± 0.001	04:45
	599.227	0.604 ± 0.001	05:09	499.147	0.777 ± 0.001	36:20	698.870	0.810 ± 0.002	36:28	498.666	0.877 ± 0.002	04:48
	698.790	0.676 ± 0.001	05:13	598.698	0.861 ± 0.002	36:25	798.940	0.873 ± 0.002	36:32	599.221	0.988 ± 0.002	04:52
	799.195	0.742 ± 0.002	05:16	698.700	0.941 ± 0.002	36:30	899.623	0.932 ± 0.002	36:36	699.297	1.090 ± 0.002	04:55

	898.941	0.805 ± 0.002	05:20	798.735	1.013 ± 0.002	36:33	949.772	0.960 ± 0.002	36:40	801.698	1.186 ± 0.002	04:59
	948.846	0.838 ± 0.002	05:24	900.141	1.080 ± 0.002	36:38	802.475	0.887 ± 0.002	36:44	901.313	1.273 ± 0.003	05:03
	802.706	0.752 ± 0.002	05:28	949.421	1.112 ± 0.002	36:41	702.242	0.828 ± 0.002	36:49	951.460	1.315 ± 0.003	05:07
	702.365	0.687 ± 0.002	05:32	803.056	1.023 ± 0.002	36:45	601.519	0.759 ± 0.001	36:54	802.020	1.194 ± 0.002	05:10
	601.211	0.614 ± 0.001	05:36	702.661	0.955 ± 0.002	36:50	500.776	0.682 ± 0.001	36:58	702.745	1.104 ± 0.002	05:14
	500.815	0.534 ± 0.001	05:40	600.956	0.876 ± 0.002	36:55	400.890	0.597 ± 0.001	37:03	599.813	1.00 ± 0.002	05:18
	400.631	0.449 ± 0.001	05:45	500.855	0.789 ± 0.001	37:00	300.589	0.496 ± 0.001	37:08	501.043	0.890 ± 0.002	05:22
	301.027	0.356 ± 0.001	05:49	400.838	0.690 ± 0.001	37:05	200.761	0.379 ± 0.001	37:13	400.134	0.767 ± 0.002	05:26
	200.501	0.249 ± 0.001	05:53	300.865	0.575 ± 0.001	37:10	176.397	0.339 ± 0.001	37:18	300.343	0.628 ± 0.001	05:30
	175.572	0.214 ± 0	05:58	200.814	0.441 ± 0.001	37:15	150.084	0.300 ± 0.001	37:24	200.713	0.471 ± 0.001	05:35
	151.012	0.180 ± 0	06:02	175.419	0.400 ± 0.001	37:20	125.579	0.261 ± 0.001	37:29	175.663	0.425 ± 0.001	05:39
	125.933	0.147 ± 0	06:07	150.341	0.358 ± 0.001	37:26	100.334	0.219 ± 0	37:35	150.450	0.377 ± 0.001	05:43
	100.642	0.111 ± 0	06:11	125.561	0.315 ± 0.001	37:31	75.381	0.173 ± 0	37:41	125.451	0.327 ± 0.001	05:47
	75.806	0.070 ± 0	06:16	100.584	0.266 ± 0.001	37:37	50.409	0.125 ± 0	37:47	100.469	0.273 ± 0.001	05:53
	50.835	0.033 ± 0	06:20	75.429	0.213 ± 0	37:42	45.358	0.113 ± 0	37:52	75.392	0.217 ± 0	05:57
	45.522	0.021 ± 0	06:25	50.393	0.156 ± 0	37:48	40.317	0.102 ± 0	37:57	50.384	0.157 ± 0	06:02
	40.436	0.013 ± 0	06:29	45.448	0.140 ± 0	37:54	35.345	0.089 ± 0	38:03	45.397	0.143 ± 0	06:07
				40.219	0.126 ± 0	37:59	30.301	0.077 ± 0	38:08	40.358	0.129 ± 0	06:12
				35.363	0.112 ± 0	38:05	25.257	0.065 ± 0	38:15	35.423	0.116 ± 0	06:18
				30.305	0.098 ± 0	38:10	20.388	0.055 ± 0	38:22	30.409	0.101 ± 0	06:23
				25.283	0.084 ± 0	38:16	17.864	0.047 ± 0	38:29	25.410	0.086 ± 0	06:28
				20.288	0.069 ± 0	38:22	15.265	0.038 ± 0	38:36	20.373	0.072 ± 0	06:34
				17.699	0.061 ± 0	38:26	13.785	0.034 ± 0	38:41	17.900	0.064 ± 0	06:39
				15.254	0.054 ± 0	38:31	12.338	0.027 ± 0	38:47	15.422	0.056 ± 0	06:44
				13.806	0.048 ± 0	38:36	11.040	0.024 ± 0	38:55	13.900	0.051 ± 0	06:47
				12.276	0.041 ± 0	38:42	10.029	0.021 ± 0	39:01	12.859	0.042 ± 0	06:51
				10.969	0.037 ± 0	38:47	8.155	0.014 ± 0	39:11	11.556	0.030 ± 0	06:55
				10.059	0.034 ± 0	38:52	6.181	0.007 ± 0	39:26	11.020	0.034 ± 0	06:59
				8.092	0.027 ± 0	38:59				10.081	0.030 ± 0	07:03
				6.037	0.019 ± 0	39:10				8.079	0.024 ± 0	07:08
				4.065	0.011 ± 0	39:30				6.047	0.017 ± 0	07:14
				2.052	0.001 ± 0	40:48				4.058	0.010 ± 0	07:23
										2.078	0.002 ± 0	08:04
298.15 K	0.495	8.3±0E-05	02:48	0.492	0.001 ± 0	02:51	0.994	0.001 ± 0	02:42	0.493	3.2±0E-04	02:50
	0.994	0.001 ± 0	02:53	0.985	0.002 ± 0	02:56	1.994	0.002 ± 0	02:47	0.987	0.002 ± 0	02:54
	1.994	0.002 ± 0	02:57	1.976	0.004 ± 0	03:00	3.996	0.005 ± 0	02:52	1.982	0.004 ± 0	02:59
	3.996	0.005 ± 0	03:02	3.955	0.010 ± 0	03:05	6.005	0.008 ± 0	02:56	3.961	0.009 ± 0	03:03
	6.005	0.008 ± 0	03:06	5.997	0.015 ± 0	03:09	8.006	0.011 ± 0	03:01	6.030	0.015 ± 0	03:07
	8.006	0.011 ± 0	03:10	8.031	0.020 ± 0	03:14	10.216	0.011 ± 0	03:05	8.038	0.020 ± 0	03:12

10.216	0.011 ± 0	03:15	10.228	0.023 ± 0	03:17	10.988	0.012 ± 0	03:09	10.218	0.023 ± 0	03:16
10.988	0.012 ± 0	03:19	10.985	0.025 ± 0	03:21	12.239	0.012 ± 0	03:14	10.991	0.024 ± 0	03:20
12.239	0.012 ± 0	03:23	12.302	0.027 ± 0	03:28	13.276	0.028 ± 0	03:19	12.329	0.025 ± 0	03:26
13.276	0.028 ± 0	03:29	13.246	0.043 ± 0	03:33	15.194	0.030 ± 0	03:23	13.200	0.041 ± 0	03:32
15.194	0.030 ± 0	03:34	15.176	0.048 ± 0	03:38	17.337	0.031 ± 0	03:27	15.185	0.046 ± 0	03:36
17.337	0.031 ± 0	03:40	17.295	0.053 ± 0	03:44	20.019	0.033 ± 0	03:31	17.488	0.051 ± 0	03:42
20.019	0.033 ± 0	03:47	20.201	0.060 ± 0	03:51	22.552	0.037 ± 0	03:36	19.965	0.059 ± 0	03:49
22.552	0.037 ± 0	03:53	22.464	0.066 ± 0	03:58	24.960	0.040 ± 0	03:42	22.585	0.064 ± 0	03:55
24.960	0.040 ± 0	04:00	24.975	0.071 ± 0	04:04	27.423	0.042 ± 0	03:48	24.886	0.070 ± 0	04:02
27.423	0.042 ± 0	04:07	27.409	0.077 ± 0	04:10	29.918	0.046 ± 0	03:54	27.473	0.076 ± 0	04:09
29.918	0.046 ± 0	04:12	29.934	0.083 ± 0	04:16	34.929	0.052 ± 0	04:01	29.964	0.082 ± 0	04:14
34.929	0.052 ± 0	04:19	34.809	0.093 ± 0	04:23	39.899	0.058 ± 0	04:07	34.816	0.093 ± 0	04:21
39.899	0.058 ± 0	04:25	39.890	0.104 ± 0	04:29	44.862	0.064 ± 0	04:14	39.910	0.103 ± 0	04:27
44.862	0.064 ± 0	04:31	44.874	0.115 ± 0	04:35	49.872	0.069 ± 0	04:20	44.862	0.114 ± 0	04:34
49.872	0.069 ± 0	04:36	49.851	0.125 ± 0	04:40	74.457	0.097 ± 0	04:27	49.955	0.125 ± 0	04:38
74.457	0.097 ± 0	04:42	73.984	0.171 ± 0	04:46	99.805	0.124 ± 0	04:34	73.979	0.172 ± 0	04:45
99.805	0.124 ± 0	04:47	99.540	0.215 ± 0	04:50	124.684	0.150 ± 0	04:40	99.570	0.220 ± 0	04:48
124.684	0.150 ± 0	04:52	124.567	0.256 ± 0	04:56	149.567	0.176 ± 0	04:46	124.527	0.264 ± 0.001	04:54
149.567	0.176 ± 0	04:57	149.407	0.294 ± 0.001	05:00	174.513	0.200 ± 0	04:52	149.616	0.306 ± 0.001	04:59
174.513	0.200 ± 0	05:02	174.900	0.331 ± 0.001	05:05	199.547	0.224 ± 0	04:58	174.418	0.345 ± 0.001	05:04
199.547	0.224 ± 0	05:06	199.652	0.365 ± 0.001	05:09	298.609	0.311 ± 0.001	05:05	199.265	0.383 ± 0.001	05:08
298.609	0.311 ± 0.001	05:10	297.473	0.480 ± 0.001	05:13	399.096	0.392 ± 0.001	05:11	297.280	0.517 ± 0.001	05:12
399.096	0.392 ± 0.001	05:14	399.049	0.581 ± 0.001	05:17	498.995	0.465 ± 0.001	05:17	398.928	0.640 ± 0.001	05:15
498.995	0.465 ± 0.001	05:17	499.050	0.667 ± 0.001	05:20	601.564	0.535 ± 0.001	05:23	499.733	0.747 ± 0.001	05:19
601.564	0.535 ± 0.001	05:21	600.512	0.742 ± 0.001	05:24	700.198	0.598 ± 0.001	05:27	600.398	0.844 ± 0.002	05:23
700.198	0.598 ± 0.001	05:25	701.510	0.810 ± 0.002	05:28	800.605	0.660 ± 0.001	05:31	700.383	0.932 ± 0.002	05:27
800.605	0.660 ± 0.001	05:29	800.831	0.869 ± 0.002	05:31	901.808	0.719 ± 0.001	05:37	800.347	1.013 ± 0.002	05:30
901.808	0.719 ± 0.001	05:32	901.128	0.923 ± 0.002	05:35	950.423	0.746 ± 0.001	05:42	900.555	1.089 ± 0.002	05:34
950.423	0.746 ± 0.002	05:36	950.945	0.948 ± 0.002	05:39	801.010	0.663 ± 0.001	05:47	950.386	1.125 ± 0.002	05:38
801.010	0.663 ± 0.001	05:40	801.168	0.874 ± 0.002	05:42	700.750	0.603 ± 0.001	05:51	801.934	1.020 ± 0.002	05:41
700.750	0.603 ± 0.001	05:43	700.863	0.816 ± 0.002	05:46	600.210	0.536 ± 0.001	05:56	702.607	0.940 ± 0.002	05:45
600.210	0.536 ± 0.001	05:47	599.907	0.749 ± 0.001	05:50	501.240	0.467 ± 0.001	06:01	599.986	0.850 ± 0.002	05:49
501.240	0.467 ± 0.001	05:51	500.129	0.674 ± 0.001	05:54	400.199	0.392 ± 0.001	06:06	500.159	0.753 ± 0.001	05:52
400.199	0.392 ± 0.001	05:55	400.668	0.589 ± 0.001	05:57	300.316	0.311 ± 0.001	06:11	400.513	0.646 ± 0.001	05:56
300.316	0.311 ± 0.001	05:59	300.487	0.490 ± 0.001	06:01	200.377	0.224 ± 0	06:18	300.427	0.527 ± 0.001	06:00
200.377	0.224 ± 0	06:04	200.564	0.373 ± 0.001	06:07	175.613	0.200 ± 0	06:25	200.570	0.391 ± 0.001	06:06
175.613	0.200 ± 0	06:08	175.639	0.337 ± 0.001	06:13	150.578	0.175 ± 0	06:32	175.569	0.351 ± 0.001	06:11
150.578	0.175 ± 0	06:15	150.429	0.301 ± 0.001	06:19	125.545	0.149 ± 0	06:39	150.427	0.310 ± 0.001	06:17
125.545	0.149 ± 0	06:21	125.527	0.263 ± 0	06:25	100.402	0.123 ± 0	06:46	125.503	0.268 ± 0.001	06:23
100.402	0.123 ± 0	06:27	100.440	0.221 ± 0	06:32	75.321	0.095 ± 0	06:53	100.408	0.223 ± 0	06:30

	75.321	0.095 ± 0	06:34	75.380	0.177 ± 0	06:38	50.287	0.067 ± 0	07:00	75.343	0.176 ± 0	06:36
	50.287	0.067 ± 0	06:41	50.368	0.129 ± 0	06:46	45.406	0.060 ± 0	07:07	50.336	0.127 ± 0	06:43
	45.406	0.060 ± 0	06:47	45.417	0.117 ± 0	06:52	40.387	0.054 ± 0	07:14	45.398	0.115 ± 0	06:50
	40.387	0.054 ± 0	06:54	40.274	0.106 ± 0	06:59	35.409	0.047 ± 0	07:21	40.338	0.104 ± 0	06:57
	35.409	0.047 ± 0	07:01	35.442	0.094 ± 0	07:06	30.415	0.039 ± 0	07:28	35.366	0.092 ± 0	07:04
	30.415	0.039 ± 0	07:08	30.424	0.082 ± 0	07:12	25.443	0.033 ± 0	07:35	30.373	0.080 ± 0	07:10
	25.443	0.033 ± 0	07:15	25.429	0.070 ± 0	07:20	20.413	0.026 ± 0	07:42	25.405	0.068 ± 0	07:17
	20.413	0.026 ± 0	07:22	20.431	0.058 ± 0	07:26	17.977	0.023 ± 0	07:49	20.412	0.056 ± 0	07:24
	17.977	0.023 ± 0	07:29	17.938	0.052 ± 0	07:33	15.458	0.020 ± 0	07:56	17.938	0.050 ± 0	07:31
	15.458	0.020 ± 0	07:36	15.419	0.046 ± 0	07:40	13.956	0.018 ± 0	08:03	15.441	0.043 ± 0	07:38
	13.956	0.017 ± 0	07:43	13.955	0.042 ± 0	07:45	12.879	0.009 ± 0	08:10	13.949	0.039 ± 0	07:44
	12.879	0.009 ± 0	07:46	12.949	0.033 ± 0	07:50	11.013	0.005 ± 0	08:22	12.926	0.030 ± 0	07:48
	11.013	0.005 ± 0	07:55	11.630	0.022 ± 0	07:53	10.032	0.003 ± 0	08:26	11.633	0.020 ± 0	07:52
	10.032	0.003 ± 0	07:59	11.032	0.026 ± 0	07:58	8.071	0 ± 0	08:32	11.033	0.024 ± 0	07:56
	8.071	0 ± 0	08:03	10.071	0.023 ± 0	08:02				10.062	0.021 ± 0	08:00
				8.059	0.018 ± 0	08:06				8.072	0.016 ± 0	08:05
				6.060	0.013 ± 0	08:12				6.021	0.010 ± 0	08:10
				4.066	0.007 ± 0	08:22				4.107	0.004 ± 0	08:21
				2.076	0 ± 0	09:01						
308.15 K	1.996	0.001 ± 0	02:40	0.987	3.65±0E-04	06:32	3.991	0.005 ± 0	02:45	1.985	0.003 ± 0	02:42
	3.986	0.003 ± 0	02:46	1.983	0.002 ± 0	06:36	5.995	0.008 ± 0	02:49	3.978	0.006 ± 0	02:47
	6.006	0.005 ± 0	02:50	3.967	0.006 ± 0	06:40	7.987	0.011 ± 0	02:53	6.014	0.010 ± 0	02:52
	8.019	0.007 ± 0	02:54	5.969	0.009 ± 0	06:45	10.216	0.012 ± 0	02:57	8.029	0.013 ± 0	02:56
	10.235	0.006 ± 0	02:59	8.010	0.013 ± 0	06:49	10.976	0.014 ± 0	03:02	10.194	0.014 ± 0	03:00
	11.025	0.007 ± 0	03:03	10.146	0.014 ± 0	06:54	12.204	0.013 ± 0	03:07	11.004	0.016 ± 0	03:04
	12.111	0.008 ± 0	03:08	11.009	0.016 ± 0	06:58	13.203	0.025 ± 0	03:11	12.191	0.017 ± 0	03:10
	13.219	0.023 ± 0	03:13	12.219	0.016 ± 0	07:03	15.193	0.027 ± 0	03:16	13.155	0.031 ± 0	03:15
	15.227	0.024 ± 0	03:17	13.268	0.030 ± 0	07:08	17.387	0.031 ± 0	03:21	15.217	0.034 ± 0	03:18
	17.506	0.028 ± 0	03:23	15.264	0.032 ± 0	07:13	19.977	0.034 ± 0	03:27	17.450	0.038 ± 0	03:25
	19.958	0.030 ± 0	03:30	17.453	0.035 ± 0	07:18	22.506	0.037 ± 0	03:34	19.980	0.042 ± 0	03:32
	22.419	0.032 ± 0	03:35	20.008	0.040 ± 0	07:23	24.858	0.040 ± 0	03:39	22.437	0.045 ± 0	03:37
	24.673	0.033 ± 0	03:42	22.503	0.044 ± 0	07:27	27.441	0.043 ± 0	03:46	24.921	0.051 ± 0	03:44
	27.494	0.036 ± 0	03:48	24.954	0.047 ± 0	07:32	30.020	0.046 ± 0	03:52	27.551	0.056 ± 0	03:50
	29.937	0.038 ± 0	03:55	27.483	0.051 ± 0	07:37	34.839	0.053 ± 0	03:59	29.958	0.060 ± 0	03:57
	34.885	0.042 ± 0	04:01	29.970	0.053 ± 0	07:42	39.955	0.060 ± 0	04:05	34.872	0.068 ± 0	04:03
	39.911	0.047 ± 0	04:08	34.599	0.060 ± 0	07:47	44.970	0.067 ± 0	04:12	39.948	0.077 ± 0	04:10
	44.969	0.051 ± 0	04:14	39.935	0.069 ± 0	07:52	49.912	0.073 ± 0	04:19	44.860	0.085 ± 0	04:16
	49.822	0.056 ± 0	04:21	44.953	0.077 ± 0	07:57	74.375	0.102 ± 0	04:25	49.923	0.093 ± 0	04:23
	74.622	0.078 ± 0	04:27	49.868	0.085 ± 0	08:02	99.729	0.130 ± 0	04:31	74.341	0.129 ± 0	04:29

99.587	0.099 ± 0	04:33	74.231	0.118 ± 0	08:07	124.605	0.157 ± 0	04:38	99.610	0.165 ± 0	04:36
124.660	0.119 ± 0	04:40	99.542	0.151 ± 0	08:11	149.421	0.183 ± 0	04:43	125.233	0.200 ± 0	04:42
149.594	0.138 ± 0	04:45	124.594	0.182 ± 0	08:15	174.510	0.208 ± 0	04:49	149.346	0.232 ± 0	04:47
174.852	0.157 ± 0	04:51	149.571	0.211 ± 0	08:19	199.729	0.232 ± 0	04:54	174.550	0.263 ± 0.001	04:53
199.493	0.176 ± 0	04:56	174.478	0.240 ± 0	08:23	298.202	0.316 ± 0.001	04:59	199.562	0.293 ± 0.001	04:58
298.285	0.245 ± 0.001	05:00	199.351	0.267 ± 0.001	08:27	398.863	0.392 ± 0.001	05:03	297.656	0.402 ± 0.001	05:02
399.008	0.310 ± 0.001	05:04	298.079	0.363 ± 0.001	08:31	499.009	0.461 ± 0.001	05:06	399.214	0.504 ± 0.001	05:05
499.681	0.370 ± 0.001	05:07	398.800	0.450 ± 0.001	08:34	598.950	0.523 ± 0.001	05:10	499.345	0.594 ± 0.001	05:09
599.216	0.426 ± 0.001	05:11	499.450	0.527 ± 0.001	08:38	697.513	0.580 ± 0.001	05:14	598.692	0.676 ± 0.001	05:13
699.074	0.477 ± 0.001	05:15	598.789	0.595 ± 0.001	08:41	800.863	0.635 ± 0.001	05:18	698.792	0.753 ± 0.001	05:17
800.650	0.527 ± 0.001	05:18	700.958	0.659 ± 0.001	08:45	900.322	0.685 ± 0.001	05:22	800.314	0.825 ± 0.002	05:21
901.692	0.575 ± 0.001	05:23	800.427	0.717 ± 0.001	08:49	950.435	0.711 ± 0.001	05:25	901.399	0.895 ± 0.002	05:24
950.977	0.596 ± 0.001	05:27	900.315	0.772 ± 0.002	08:52	801.068	0.643 ± 0.001	05:29	950.486	0.927 ± 0.002	05:28
801.146	0.531 ± 0.001	05:30	950.176	0.798 ± 0.002	08:56	701.067	0.592 ± 0.001	05:33	801.713	0.833 ± 0.002	05:31
700.674	0.482 ± 0.001	05:34	800.943	0.724 ± 0.001	09:00	600.693	0.535 ± 0.001	05:36	702.277	0.762 ± 0.001	05:35
601.104	0.429 ± 0.001	05:38	700.922	0.667 ± 0.001	09:04	500.211	0.473 ± 0.001	05:40	599.767	0.683 ± 0.001	05:39
500.130	0.371 ± 0.001	05:42	601.088	0.604 ± 0.001	09:08	400.181	0.405 ± 0.001	05:44	500.187	0.600 ± 0.001	05:43
400.602	0.310 ± 0.001	05:45	501.058	0.533 ± 0.001	09:11	300.563	0.329 ± 0.001	05:48	400.838	0.509 ± 0.001	05:46
300.197	0.244 ± 0.001	05:50	400.216	0.456 ± 0.001	09:15	200.669	0.244 ± 0	05:53	300.446	0.409 ± 0.001	05:51
200.282	0.174 ± 0	05:55	300.332	0.370 ± 0.001	09:19	176.324	0.217 ± 0	06:00	200.684	0.298 ± 0.001	05:58
175.585	0.154 ± 0	06:02	200.540	0.273 ± 0.001	09:23	150.525	0.190 ± 0	06:07	175.532	0.266 ± 0.001	06:05
150.557	0.134 ± 0	06:10	175.736	0.245 ± 0	09:27	125.675	0.163 ± 0	06:14	150.515	0.234 ± 0	06:12
125.450	0.113 ± 0	06:17	150.366	0.215 ± 0	09:32	100.741	0.136 ± 0	06:21	125.598	0.201 ± 0	06:18
100.410	0.092 ± 0	06:23	125.259	0.185 ± 0	09:37	75.611	0.106 ± 0	06:27	100.402	0.165 ± 0	06:25
75.406	0.070 ± 0	06:29	100.366	0.153 ± 0	09:42	50.608	0.075 ± 0	06:33	75.345	0.129 ± 0	06:30
50.341	0.049 ± 0	06:35	75.299	0.119 ± 0	09:46	45.674	0.067 ± 0	06:39	50.268	0.091 ± 0	06:37
45.375	0.045 ± 0	06:41	50.316	0.085 ± 0	09:51	40.625	0.060 ± 0	06:46	45.363	0.083 ± 0	06:44
40.450	0.040 ± 0	06:48	45.393	0.077 ± 0	09:56	35.789	0.052 ± 0	06:53	40.282	0.074 ± 0	06:51
35.390	0.035 ± 0	06:55	40.367	0.070 ± 0	10:01	30.780	0.044 ± 0	07:00	35.335	0.066 ± 0	06:58
30.397	0.030 ± 0	07:02	35.359	0.061 ± 0	10:06	25.811	0.037 ± 0	07:07	30.428	0.058 ± 0	07:05
25.453	0.024 ± 0	07:09	30.351	0.053 ± 0	10:11	20.876	0.029 ± 0	07:14	25.372	0.050 ± 0	07:12
20.447	0.019 ± 0	07:16	25.356	0.046 ± 0	10:15	18.420	0.026 ± 0	07:21	20.403	0.043 ± 0	07:19
17.957	0.017 ± 0	07:23	20.386	0.038 ± 0	10:20	15.958	0.022 ± 0	07:28	17.897	0.037 ± 0	07:26
15.436	0.014 ± 0	07:30	17.879	0.034 ± 0	10:25	14.473	0.019 ± 0	07:33	15.357	0.032 ± 0	07:32
13.944	0.012 ± 0	07:34	15.413	0.030 ± 0	10:30	13.375	0.013 ± 0	07:37	13.922	0.029 ± 0	07:36
12.903	0.005 ± 0	07:39	13.941	0.027 ± 0	10:34	12.137	0.006 ± 0	07:42	12.908	0.022 ± 0	07:40
11.031	0.001 ± 0	07:48	12.799	0.019 ± 0	10:39	10.717	0.007 ± 0	07:46	11.540	0.013 ± 0	07:45
			11.566	0.010 ± 0	10:43	8.772	0.004 ± 0	07:51	10.049	0.015 ± 0	07:49
			11.037	0.014 ± 0	10:47	1.099	0.015 ± 0	08:18	8.073	0.011 ± 0	07:54
			10.036	0.012 ± 0	10:51	1.033	0.002 ± 0	08:29	6.009	0.007 ± 0	07:58

8.034	0.008 ± 0	10:55	1.052	0.001 ± 0	08:33	4.065	0.003 ± 0	08:06
6.080	0.005 ± 0	11:00	1.071	0.001 ± 0	08:37			
4.078	0.001 ± 0	11:06	1.090	4.20±0E-04	08:41			
			1.109	5.18±0E-05	08:45			

Table S6. CO_2 adsorption data on carbon materials at (a) 288.15 K, (b) 298.15 K and (c) 308.15 K

	BPL Carbon			C564			C569			C1005		
	Absolute Pressure (mbar)	n (mmol/g)	Elapsed Time (h:min)	Absolute Pressure (mbar)	n (mmol/g)	Elapsed Time (h:min)	Absolute Pressure (mbar)	n (mmol/g)	Elapsed Time (h:min)	Absolute Pressure (mbar)	n (mmol/g)	Elapsed Time (h:min)
288.15 K	0.119	0.001 ± 0	04:15	0.065	0.001 ± 0	04:32	0.127	0.001 ± 0	04:08	0.126	0.001 ± 0	02:17
	0.241	0.002 ± 0	04:24	0.127	0.002 ± 0	04:39	0.248	0.002 ± 0	04:14	0.250	0.003 ± 0	02:23
	0.481	0.003 ± 0	04:30	0.249	0.003 ± 0	04:46	0.502	0.005 ± 0	04:20	0.499	0.005 ± 0	02:29
	0.964	0.007 ± 0	04:37	0.500	0.007 ± 0	04:52	0.999	0.010 ± 0	04:29	1.001	0.011 ± 0	02:34
	1.941	0.014 ± 0	04:42	1.002	0.013 ± 0	04:58	2.003	0.020 ± 0	04:35	2.005	0.022 ± 0	02:40
	3.852	0.027 ± 0	04:48	2.000	0.026 ± 0	05:05	3.990	0.039 ± 0	04:43	3.805	0.041 ± 0	02:45
	5.953	0.041 ± 0	04:53	4.018	0.052 ± 0	05:11	5.924	0.057 ± 0	04:50	5.905	0.063 ± 0	02:50
	7.965	0.054 ± 0	04:59	5.974	0.075 ± 0	05:17	7.920	0.075 ± 0	04:55	7.953	0.083 ± 0	02:54
	10.147	0.064 ± 0	05:04	7.987	0.098 ± 0	05:21	10.119	0.091 ± 0	05:01	10.136	0.100 ± 0	02:59
	11.099	0.068 ± 0	05:08	10.175	0.118 ± 0	05:26	11.057	0.098 ± 0	05:07	11.031	0.107 ± 0	03:04
	12.134	0.073 ± 0	05:14	11.115	0.127 ± 0	05:32	12.115	0.106 ± 0	05:13	12.081	0.115 ± 0	03:09
	13.119	0.095 ± 0	05:19	12.163	0.136 ± 0	05:36	13.086	0.129 ± 0	05:17	13.109	0.141 ± 0	03:14
	15.147	0.106 ± 0	05:24	13.154	0.162 ± 0	05:40	15.088	0.145 ± 0	05:22	15.092	0.160 ± 0	03:19
	17.426	0.118 ± 0	05:30	15.157	0.182 ± 0	05:44	17.118	0.160 ± 0	05:28	17.126	0.176 ± 0	03:24
	19.914	0.131 ± 0	05:35	17.176	0.200 ± 0	05:49	19.958	0.180 ± 0	05:33	19.860	0.199 ± 0	03:29
	22.607	0.145 ± 0	05:39	19.905	0.224 ± 0	05:54	22.543	0.198 ± 0	05:38	22.477	0.219 ± 0	03:33
	24.968	0.156 ± 0	05:43	22.611	0.248 ± 0	05:58	24.914	0.214 ± 0	05:42	24.871	0.237 ± 0	03:38
	27.505	0.168 ± 0	05:47	25.045	0.268 ± 0.001	06:03	27.447	0.231 ± 0	05:46	27.455	0.257 ± 0.001	03:43
	29.944	0.180 ± 0	05:51	27.563	0.289 ± 0.001	06:07	29.920	0.247 ± 0	05:50	29.933	0.275 ± 0.001	03:48
	34.686	0.202 ± 0.001	05:57	30.073	0.309 ± 0.001	06:12	34.582	0.275 ± 0.001	05:56	34.703	0.309 ± 0.001	03:53
	39.868	0.225 ± 0.001	06:01	34.758	0.344 ± 0.001	06:16	39.778	0.306 ± 0.001	06:00	39.792	0.343 ± 0.001	03:58
	44.911	0.246 ± 0.001	06:05	40.077	0.382 ± 0.001	06:20	44.929	0.335 ± 0.001	06:04	44.866	0.376 ± 0.001	04:03
	50.008	0.266 ± 0.001	06:09	45.083	0.416 ± 0.001	06:24	49.969	0.362 ± 0.001	06:08	50.085	0.410 ± 0.001	04:08
	73.482	0.354 ± 0.001	06:14	50.137	0.449 ± 0.001	06:28	73.315	0.474 ± 0.001	06:13	72.940	0.541 ± 0.001	04:13
	100.128	0.443 ± 0.001	06:18	73.288	0.583 ± 0.002	06:32	99.842	0.584 ± 0.001	06:17	99.874	0.679 ± 0.001	04:18
	125.112	0.520 ± 0.001	06:22	100.280	0.716 ± 0.002	06:36	124.651	0.674 ± 0.001	06:21	125.250	0.797 ± 0.002	04:22
	149.533	0.591 ± 0.002	06:27	125.405	0.825 ± 0.002	06:40	149.312	0.755 ± 0.001	06:26	150.120	0.903 ± 0.002	04:27
	174.310	0.657 ± 0.002	06:31	149.997	0.919 ± 0.002	06:43	174.266	0.830 ± 0.002	06:29	174.301	0.999 ± 0.002	04:31
	199.323	0.721 ± 0.002	06:34	175.717	1.008 ± 0.003	06:47	199.276	0.899 ± 0.002	06:33	199.247	1.092 ± 0.002	04:36
	296.250	0.942 ± 0.002	06:38	200.167	1.086 ± 0.003	06:51	296.013	1.119 ± 0.002	06:37	296.010	1.407 ± 0.003	04:40
	399.247	1.147 ± 0.003	06:41	296.771	1.344 ± 0.003	06:54	398.602	1.307 ± 0.002	06:40	399.654	1.692 ± 0.003	04:45
	498.467	1.324 ± 0.003	06:45	401.281	1.564 ± 0.003	06:58	498.528	1.458 ± 0.003	06:44	499.966	1.934 ± 0.004	04:49
	598.715	1.488 ± 0.004	06:49	500.388	1.737 ± 0.003	07:02	598.650	1.588 ± 0.003	06:48	598.731	2.147 ± 0.004	04:54

	698.714	1.640 ± 0.004	06:53	601.307	1.888 ± 0.004	07:05	698.422	1.702 ± 0.003	06:52	698.938	2.344 ± 0.005	04:58
	798.573	1.782 ± 0.005	06:57	698.845	2.017 ± 0.004	07:09	798.508	1.803 ± 0.003	06:56	798.783	2.526 ± 0.005	05:02
	898.571	1.917 ± 0.005	07:00	798.741	2.135 ± 0.004	07:13	898.901	1.896 ± 0.004	06:59	899.044	2.696 ± 0.005	05:05
	948.851	1.983 ± 0.005	07:04	898.594	2.244 ± 0.004	07:17	948.887	1.940 ± 0.004	07:03	948.968	2.777 ± 0.006	05:09
	803.322	1.799 ± 0.005	07:08	948.947	2.297 ± 0.004	07:20	803.916	1.821 ± 0.003	07:07	804.093	2.547 ± 0.005	05:13
	703.903	1.661 ± 0.004	07:12	801.747	2.151 ± 0.004	07:24	703.113	1.724 ± 0.003	07:11	704.431	2.370 ± 0.005	05:17
	600.455	1.505 ± 0.004	07:15	701.360	2.036 ± 0.004	07:28	600.000	1.609 ± 0.003	07:14	600.133	2.168 ± 0.004	05:22
	500.267	1.341 ± 0.003	07:19	598.987	1.903 ± 0.004	07:31	500.868	1.481 ± 0.003	07:18	500.400	1.953 ± 0.004	05:25
	400.363	1.163 ± 0.003	07:23	499.437	1.754 ± 0.003	07:35	400.604	1.330 ± 0.002	07:22	400.997	1.715 ± 0.003	05:29
	300.848	0.965 ± 0.002	07:26	399.832	1.579 ± 0.003	07:39	300.867	1.150 ± 0.002	07:25	301.295	1.442 ± 0.003	05:34
	200.707	0.738 ± 0.002	07:30	300.407	1.369 ± 0.003	07:43	201.229	0.924 ± 0.002	07:29	201.211	1.120 ± 0.002	05:38
	175.275	0.672 ± 0.002	07:34	201.440	1.106 ± 0.002	07:47	175.165	0.851 ± 0.002	07:33	175.622	1.023 ± 0.002	05:42
	150.132	0.604 ± 0.002	07:38	174.829	1.021 ± 0.002	07:50	150.078	0.776 ± 0.001	07:37	150.203	0.922 ± 0.002	05:47
	125.332	0.532 ± 0.001	07:42	149.752	0.932 ± 0.002	07:55	125.360	0.694 ± 0.001	07:41	125.355	0.816 ± 0.002	05:51
	100.370	0.455 ± 0.001	07:46	125.174	0.837 ± 0.002	08:00	100.510	0.603 ± 0.001	07:45	100.458	0.700 ± 0.001	05:55
	75.376	0.370 ± 0.001	07:49	100.325	0.730 ± 0.001	08:04	75.632	0.500 ± 0.001	07:48	75.508	0.572 ± 0.001	05:59
	50.414	0.277 ± 0.001	07:54	75.653	0.609 ± 0.001	08:08	50.732	0.381 ± 0.001	07:54	50.594	0.429 ± 0.001	06:04
	45.284	0.255 ± 0.001	07:59	50.796	0.466 ± 0.001	08:12	45.285	0.351 ± 0.001	07:58	45.335	0.394 ± 0.001	06:09
	40.285	0.233 ± 0.001	08:03	45.268	0.429 ± 0.001	08:17	40.224	0.322 ± 0.001	08:02	40.205	0.359 ± 0.001	06:14
	35.339	0.201 ± 0.001	08:07	40.141	0.394 ± 0.001	08:22	35.398	0.292 ± 0.001	08:06	35.327	0.324 ± 0.001	06:19
	30.379	0.187 ± 0	08:11	35.241	0.358 ± 0.001	08:27	30.418	0.261 ± 0	08:10	30.402	0.288 ± 0.001	06:24
	25.388	0.163 ± 0	08:15	30.288	0.319 ± 0.001	08:33	25.444	0.229 ± 0	08:14	25.391	0.251 ± 0.001	06:29
	20.392	0.137 ± 0	08:20	25.336	0.279 ± 0.001	08:37	20.463	0.194 ± 0	08:19	20.422	0.212 ± 0	06:34
	17.893	0.124 ± 0	08:25	20.438	0.237 ± 0	08:42	17.913	0.175 ± 0	08:24	17.875	0.190 ± 0	06:39
	15.424	0.109 ± 0	08:31	17.826	0.213 ± 0	08:47	15.415	0.156 ± 0	08:29	15.341	0.168 ± 0	06:4[illegible]
	13.897	0.100 ± 0	08:36	15.344	0.190 ± 0	08:52	13.922	0.142 ± 0	08:36	13.891	0.154 ± 0	06:4[illegible]
	12.929	0.085 ± 0	08:41	13.863	0.174 ± 0	08:57	12.875	0.125 ± 0	08:40	12.810	0.136 ± 0	06:5[illegible]
	11.482	0.069 ± 0	08:45	12.806	0.154 ± 0	09:03	11.489	0.106 ± 0	08:45	11.455	0.117 ± 0	06:5[illegible]
	10.130	0.068 ± 0	08:50	11.445	0.133 ± 0	09:08	9.871	0.101 ± 0	08:50	9.881	0.109 ± 0	07:0[illegible]
	8.227	0.056 ± 0	08:56	9.857	0.125 ± 0	09:12	8.331	0.087 ± 0	08:56	8.322	0.094 ± 0	07:0[illegible]
	6.052	0.042 ± 0	09:03	8.365	0.108 ± 0	09:17	6.198	0.067 ± 0	09:08	6.154	0.072 ± 0	07:1[illegible]
	4.021	0.028 ± 0	09:19	6.232	0.084 ± 0	09:25	4.033	0.046 ± 0	09:30	4.161	0.049 ± 0	07:4[illegible]
	2.076	0.012 ± 0	10:18	4.035	0.057 ± 0	09:43	2.062	0.024 ± 0	10:26	2.057	0.023 ± 0	08:5[illegible]
	1.030	0.001 ± 0	12:18	2.058	0.029 ± 0	10:34	1.010	0.011 ± 0	12:25	1.039	0.009 ± 0	10:5[illegible]
				1.013	0.013 ± 0	12:11	0.515	0.003 ± 0	15:10			
				0.513	0.004 ± 0	15:12						
298.15 K	0.246	0.001 ± 0	03:30	0.252	0.001 ± 0	02:36	0.240	0.001 ± 0	02:29	0.251	0.001 ± 0	02:3[illegible]
	0.489	0.002 ± 0	03:35	0.501	0.004 ± 0	02:44	0.501	0.003 ± 0	02:39	0.477	0.003 ± 0	02:3[illegible]
	0.975	0.005 ± 0	03:39	0.998	0.008 ± 0	02:52	0.966	0.007 ± 0	02:46	1.001	0.007 ± 0	02:4[illegible]

1.951	0.010 ± 0	03:43	1.910	0.016 ± 0	02:57	1.904	0.013 ± 0	02:51	1.910	0.013 ± 0	02:53
3.910	0.020 ± 0	03:48	4.010	0.034 ± 0	03:03	3.810	0.027 ± 0	02:56	3.805	0.027 ± 0	02:59
5.966	0.030 ± 0	03:53	5.951	0.050 ± 0	03:07	5.919	0.041 ± 0	03:01	5.924	0.043 ± 0	03:04
7.957	0.039 ± 0	03:57	7.939	0.066 ± 0	03:12	7.941	0.055 ± 0	03:06	7.947	0.057 ± 0	03:08
10.168	0.045 ± 0	04:01	10.179	0.080 ± 0	03:16	10.139	0.066 ± 0	03:10	10.137	0.069 ± 0	03:13
10.916	0.049 ± 0	04:06	11.064	0.084 ± 0	03:21	11.078	0.071 ± 0	03:14	11.098	0.074 ± 0	03:17
12.173	0.052 ± 0	04:10	12.140	0.090 ± 0	03:26	12.161	0.076 ± 0	03:18	11.978	0.079 ± 0	03:23
13.130	0.077 ± 0	04:14	13.150	0.114 ± 0	03:30	13.136	0.097 ± 0	03:25	13.168	0.102 ± 0	03:27
15.167	0.085 ± 0	04:19	15.152	0.128 ± 0	03:37	15.149	0.109 ± 0	03:30	15.232	0.115 ± 0	03:32
17.354	0.094 ± 0	04:25	17.165	0.142 ± 0	03:43	17.150	0.122 ± 0	03:35	17.200	0.127 ± 0	03:39
19.941	0.105 ± 0	04:29	19.851	0.160 ± 0	03:48	19.903	0.137 ± 0	03:41	19.864	0.142 ± 0	03:44
22.463	0.114 ± 0	04:34	22.426	0.177 ± 0	03:52	22.419	0.150 ± 0	03:46	22.443	0.157 ± 0	03:49
24.950	0.124 ± 0	04:38	24.889	0.192 ± 0	03:56	24.976	0.164 ± 0	03:50	24.894	0.171 ± 0	03:53
27.442	0.134 ± 0	04:42	27.438	0.208 ± 0	04:00	27.471	0.177 ± 0	03:54	27.406	0.186 ± 0	03:57
30.101	0.144 ± 0	04:47	30.097	0.225 ± 0	04:05	29.927	0.190 ± 0	03:59	29.907	0.199 ± 0	04:02
34.908	0.161 ± 0	04:51	34.745	0.251 ± 0	04:08	34.737	0.213 ± 0	04:03	34.703	0.225 ± 0	04:06
40.008	0.178 ± 0	04:55	39.883	0.280 ± 0.001	04:12	39.928	0.238 ± 0	04:07	39.860	0.252 ± 0	04:09
44.932	0.195 ± 0.001	05:00	44.858	0.307 ± 0.001	04:16	45.024	0.262 ± 0	04:11	44.967	0.279 ± 0.001	04:13
50.108	0.211 ± 0.001	05:04	50.174	0.334 ± 0.001	04:20	49.886	0.284 ± 0.001	04:15	49.899	0.303 ± 0.001	04:17
73.948	0.283 ± 0.001	05:07	73.290	0.441 ± 0.001	04:23	73.253	0.377 ± 0.001	04:18	73.383	0.410 ± 0.001	04:21
99.790	0.353 ± 0.001	05:11	99.516	0.548 ± 0.001	04:27	99.957	0.472 ± 0.001	04:22	99.968	0.519 ± 0.001	04:24
124.575	0.416 ± 0.001	05:15	124.383	0.637 ± 0.001	04:31	125.100	0.551 ± 0.001	04:25	124.730	0.612 ± 0.001	04:28
149.921	0.476 ± 0.001	05:18	149.371	0.719 ± 0.001	04:34	150.219	0.623 ± 0.001	04:29	150.225	0.702 ± 0.001	04:32
174.443	0.531 ± 0.001	05:22	174.324	0.793 ± 0.001	04:38	174.375	0.687 ± 0.001	04:33	174.486	0.781 ± 0.001	04:35
200.509	0.587 ± 0.002	05:26	200.320	0.865 ± 0.002	04:42	199.469	0.749 ± 0.001	04:36	199.226	0.858 ± 0.002	04:39
297.270	0.774 ± 0.002	05:29	296.552	1.089 ± 0.002	04:45	296.136	0.950 ± 0.002	04:40	295.440	1.120 ± 0.002	04:43
399.048	0.949 ± 0.002	05:33	398.794	1.281 ± 0.002	04:49	399.505	1.125 ± 0.002	04:44	399.709	1.363 ± 0.003	04:46
499.602	1.104 ± 0.003	05:36	498.625	1.437 ± 0.003	04:53	499.973	1.267 ± 0.002	04:47	498.778	1.566 ± 0.003	04:50
599.689	1.246 ± 0.003	05:40	599.296	1.573 ± 0.003	04:56	599.419	1.391 ± 0.003	04:51	598.423	1.750 ± 0.003	04:54
698.765	1.377 ± 0.004	05:44	699.650	1.692 ± 0.003	05:00	699.374	1.501 ± 0.003	04:55	699.320	1.920 ± 0.004	04:57
798.811	1.503 ± 0.004	05:47	799.074	1.797 ± 0.003	05:03	799.483	1.600 ± 0.003	04:58	799.446	2.076 ± 0.004	05:01
898.835	1.623 ± 0.004	05:51	898.488	1.892 ± 0.004	05:07	900.465	1.692 ± 0.003	05:02	900.574	2.222 ± 0.004	05:04
948.880	1.681 ± 0.004	05:54	948.597	1.939 ± 0.004	05:10	948.898	1.734 ± 0.003	05:05	948.913	2.291 ± 0.004	05:08
803.436	1.521 ± 0.004	05:58	802.626	1.810 ± 0.003	05:14	802.835	1.613 ± 0.003	05:09	803.871	2.094 ± 0.004	05:12
702.147	1.399 ± 0.004	06:02	703.562	1.709 ± 0.003	05:18	701.961	1.516 ± 0.003	05:13	703.044	1.942 ± 0.004	05:15
600.320	1.265 ± 0.003	06:05	600.504	1.588 ± 0.003	05:21	601.431	1.407 ± 0.003	05:16	601.408	1.773 ± 0.003	05:19
500.450	1.122 ± 0.003	06:09	500.736	1.454 ± 0.003	05:25	500.410	1.283 ± 0.002	05:20	500.525	1.587 ± 0.003	05:23
400.447	0.967 ± 0.003	06:13	401.067	1.298 ± 0.002	05:29	400.908	1.141 ± 0.002	05:24	400.687	1.384 ± 0.003	05:26
300.307	0.795 ± 0.002	06:16	301.015	1.111 ± 0.002	05:33	300.744	0.973 ± 0.002	05:28	300.978	1.153 ± 0.002	05:30
200.505	0.601 ± 0.002	06:20	201.115	0.880 ± 0.002	05:37	201.129	0.768 ± 0.001	05:32	201.085	0.883 ± 0.002	05:34

	175.342	0.546 ± 0.001	06:24	175.493	0.808 ± 0.002	05:41	175.241	0.703 ± 0.001	05:35	175.167	0.802 ± 0.002	05:38
	150.312	0.489 ± 0.001	06:27	150.158	0.732 ± 0.001	05:45	150.183	0.636 ± 0.001	05:39	150.131	0.719 ± 0.001	05:42
	125.397	0.429 ± 0.001	06:31	125.404	0.651 ± 0.001	05:49	125.557	0.565 ± 0.001	05:44	125.525	0.633 ± 0.001	05:47
	100.319	0.364 ± 0.001	06:35	100.579	0.562 ± 0.001	05:53	100.472	0.485 ± 0.001	05:48	100.425	0.538 ± 0.001	05:50
	75.385	0.295 ± 0.001	06:39	75.491	0.460 ± 0.001	05:57	75.546	0.398 ± 0.001	05:52	75.497	0.435 ± 0.001	05:5[illegible]
	50.317	0.220 ± 0.001	06:43	50.603	0.345 ± 0.001	06:01	50.613	0.298 ± 0.001	05:56	50.512	0.321 ± 0.001	05:59
	45.356	0.203 ± 0.001	06:47	45.302	0.317 ± 0.001	06:05	45.330	0.274 ± 0.001	06:00	45.298	0.295 ± 0.001	06:0[illegible]
	40.269	0.185 ± 0	06:51	40.249	0.290 ± 0.001	06:10	40.227	0.250 ± 0	06:04	40.300	0.268 ± 0.001	06:07
	35.395	0.167 ± 0	06:55	35.392	0.261 ± 0	06:13	35.337	0.226 ± 0	06:08	35.304	0.241 ± 0	06:1[illegible]
	30.345	0.148 ± 0	06:59	30.386	0.232 ± 0	06:17	30.392	0.200 ± 0	06:12	30.370	0.213 ± 0	06:1[illegible]
	25.356	0.130 ± 0	07:02	25.389	0.201 ± 0	06:21	25.374	0.174 ± 0	06:16	25.361	0.185 ± 0	06:19
	20.358	0.109 ± 0	07:07	20.415	0.169 ± 0	06:26	20.402	0.146 ± 0	06:20	20.402	0.155 ± 0	06:24
	17.887	0.098 ± 0	07:12	18.210	0.153 ± 0	06:33	17.878	0.132 ± 0	06:24	17.863	0.139 ± 0	06:29
	15.371	0.088 ± 0	07:18	15.385	0.132 ± 0	06:38	15.396	0.116 ± 0	06:31	15.414	0.122 ± 0	06:3[illegible]
	13.933	0.081 ± 0	07:24	13.963	0.122 ± 0	06:44	13.932	0.106 ± 0	06:36	13.900	0.111 ± 0	06:4[illegible]
	12.917	0.066 ± 0	07:30	12.961	0.105 ± 0	06:49	12.854	0.091 ± 0	06:41	12.914	0.096 ± 0	06:48
	11.548	0.048 ± 0	07:34	11.513	0.087 ± 0	06:54	11.496	0.075 ± 0	06:45	11.500	0.079 ± 0	06:5[illegible]
	10.078	0.050 ± 0	07:39	9.871	0.082 ± 0	06:59	9.863	0.071 ± 0	06:50	9.869	0.075 ± 0	06:58
	8.127	0.041 ± 0	07:43	8.299	0.069 ± 0	07:04	8.253	0.060 ± 0	06:56	8.261	0.063 ± 0	07:0[illegible]
	6.024	0.030 ± 0	07:49	6.151	0.051 ± 0	07:14	6.113	0.046 ± 0	07:04	6.132	0.047 ± 0	07:22
	4.018	0.019 ± 0	08:03	4.108	0.033 ± 0	07:32	4.041	0.031 ± 0	07:15	4.079	0.029 ± 0	07:59
	2.077	0.006 ± 0	09:02	2.036	0.012 ± 0	08:53	2.052	0.014 ± 0	07:59	2.069	0.009 ± 0	10:07
							1.042	0.004 ± 0	09:40			
308.15 K	0.246	0.001 ± 0	03:29	0.120	0.001 ± 0	02:45	0.242	0.001 ± 0	03:31	0.242	0.001 ± 0	02:52
	0.490	0.002 ± 0	03:34	0.240	0.001 ± 0	02:50	0.480	0.002 ± 0	03:36	0.480	0.002 ± 0	02:57
	0.985	0.003 ± 0	03:38	0.479	0.003 ± 0	02:54	0.959	0.005 ± 0	03:40	0.960	0.005 ± 0	03:01
	1.964	0.007 ± 0	03:42	0.958	0.006 ± 0	02:59	1.916	0.010 ± 0	03:45	1.916	0.010 ± 0	03:06
	3.909	0.014 ± 0	03:46	1.921	0.012 ± 0	03:03	3.839	0.020 ± 0	03:50	3.841	0.019 ± 0	03:10
	5.970	0.022 ± 0	03:50	3.844	0.023 ± 0	03:08	5.933	0.031 ± 0	03:54	5.931	0.030 ± 0	03:14
	7.972	0.029 ± 0	03:55	5.943	0.036 ± 0	03:12	7.965	0.041 ± 0	03:59	7.958	0.040 ± 0	03:19
	10.128	0.033 ± 0	04:00	7.953	0.048 ± 0	03:16	10.105	0.050 ± 0	04:03	10.154	0.049 ± 0	03:2[illegible]
	10.924	0.036 ± 0	04:04	10.130	0.057 ± 0	03:20	11.065	0.053 ± 0	04:07	11.086	0.052 ± 0	03:28
	12.149	0.038 ± 0	04:08	11.098	0.061 ± 0	03:25	12.096	0.058 ± 0	04:11	12.107	0.056 ± 0	03:3[illegible]
	13.132	0.056 ± 0	04:12	12.040	0.065 ± 0	03:30	13.140	0.075 ± 0	04:14	13.141	0.073 ± 0	03:37
	15.145	0.062 ± 0	04:16	13.226	0.086 ± 0	03:35	15.120	0.083 ± 0	04:18	15.151	0.082 ± 0	03:41
	17.304	0.068 ± 0	04:19	15.283	0.097 ± 0	03:39	17.204	0.093 ± 0	04:22	17.164	0.092 ± 0	03:46
	19.922	0.076 ± 0	04:23	17.352	0.106 ± 0	03:44	19.871	0.104 ± 0	04:26	20.044	0.105 ± 0	03:51
	22.454	0.083 ± 0	04:27	19.914	0.119 ± 0	03:49	22.428	0.115 ± 0	04:29	22.453	0.115 ± 0	03:5[illegible]
	24.941	0.090 ± 0	04:31	22.514	0.132 ± 0	03:53	24.947	0.126 ± 0	04:33	24.911	0.126 ± 0	04:00

27.378	0.097 ± 0	04:34	24.940	0.144 ± 0	03:58	27.459	0.137 ± 0	04:36	27.395	0.137 ± 0	04:0[illegible]
29.908	0.104 ± 0	04:38	27.479	0.157 ± 0	04:03	29.906	0.147 ± 0	04:40	29.922	0.147 ± 0	04:1[illegible]
34.858	0.118 ± 0	04:42	30.065	0.169 ± 0	04:08	34.768	0.167 ± 0	04:43	34.729	0.166 ± 0	04:1[illegible]
39.962	0.132 ± 0	04:45	34.966	0.191 ± 0	04:13	39.874	0.187 ± 0	04:47	39.901	0.187 ± 0	04:2[illegible]
44.888	0.144 ± 0	04:49	40.049	0.213 ± 0	04:19	44.917	0.206 ± 0	04:50	44.873	0.205 ± 0	04:2[illegible]
49.863	0.156 ± 0	04:52	45.419	0.237 ± 0	04:24	49.874	0.224 ± 0	04:54	49.827	0.224 ± 0	04:3[illegible]
73.939	0.212 ± 0	04:56	50.345	0.257 ± 0.001	04:28	73.422	0.302 ± 0.001	04:58	73.218	0.307 ± 0.001	04:3[illegible]
99.886	0.267 ± 0.001	05:00	73.956	0.345 ± 0.001	04:33	99.580	0.380 ± 0.001	05:01	99.544	0.393 ± 0.001	04:4[illegible]
124.667	0.317 ± 0.001	05:03	100.158	0.434 ± 0.001	04:38	124.718	0.448 ± 0.001	05:05	124.619	0.469 ± 0.001	04:4[illegible]
149.635	0.363 ± 0.001	05:07	125.107	0.510 ± 0.001	04:42	149.694	0.511 ± 0.001	05:09	149.450	0.540 ± 0.001	04:4[illegible]
174.706	0.408 ± 0.001	05:11	150.185	0.579 ± 0.001	04:46	174.331	0.568 ± 0.001	05:12	174.474	0.606 ± 0.001	04:5[illegible]
199.873	0.451 ± 0.001	05:14	174.556	0.642 ± 0.001	04:52	199.363	0.622 ± 0.001	05:16	199.704	0.671 ± 0.001	04:5[illegible]
297.855	0.603 ± 0.001	05:18	199.444	0.703 ± 0.001	04:56	296.347	0.803 ± 0.001	05:19	296.317	0.891 ± 0.002	05:0[illegible]
399.380	0.743 ± 0.002	05:21	297.391	0.905 ± 0.002	05:00	399.480	0.962 ± 0.002	05:23	398.874	1.093 ± 0.002	05:0[illegible]
500.046	0.870 ± 0.002	05:25	400.172	1.080 ± 0.002	05:04	499.660	1.095 ± 0.002	05:27	498.812	1.268 ± 0.002	05:1[illegible]
600.196	0.987 ± 0.002	05:29	498.663	1.221 ± 0.002	05:08	599.950	1.212 ± 0.002	05:30	598.830	1.427 ± 0.003	05:1[illegible]
698.903	1.095 ± 0.002	05:32	598.663	1.347 ± 0.003	05:11	698.625	1.315 ± 0.002	05:34	698.856	1.574 ± 0.003	05:1[illegible]
799.408	1.200 ± 0.002	05:36	698.826	1.459 ± 0.003	05:15	799.298	1.411 ± 0.003	05:37	798.601	1.710 ± 0.003	05:2[illegible]
900.205	1.301 ± 0.003	05:39	798.836	1.559 ± 0.003	05:19	898.981	1.501 ± 0.003	05:41	898.751	1.838 ± 0.003	05:2[illegible]
948.483	1.350 ± 0.003	05:43	899.055	1.651 ± 0.003	05:23	949.168	1.545 ± 0.003	05:45	948.481	1.900 ± 0.003	05:2[illegible]
803.697	1.215 ± 0.002	05:47	948.598	1.694 ± 0.003	05:26	803.649	1.426 ± 0.003	05:48	805.014	1.728 ± 0.003	05:3[illegible]
703.461	1.114 ± 0.002	05:50	803.278	1.572 ± 0.003	05:30	702.739	1.332 ± 0.002	05:52	703.196	1.593 ± 0.003	05:3[illegible]
600.426	1.002 ± 0.002	05:54	702.159	1.474 ± 0.003	05:34	601.249	1.227 ± 0.002	05:56	600.210	1.444 ± 0.003	05:3[illegible]
500.327	0.886 ± 0.002	05:58	600.854	1.362 ± 0.003	05:37	500.420	1.110 ± 0.002	05:59	501.126	1.287 ± 0.002	05:4[illegible]
400.555	0.761 ± 0.002	06:01	500.942	1.236 ± 0.002	05:41	400.518	0.979 ± 0.002	06:03	400.592	1.111 ± 0.002	05:4[illegible]
300.748	0.623 ± 0.001	06:05	400.528	1.092 ± 0.002	05:45	300.970	0.827 ± 0.001	06:07	300.607	0.914 ± 0.002	05:5[illegible]
200.597	0.469 ± 0.001	06:09	300.864	0.923 ± 0.002	05:49	201.008	0.642 ± 0.001	06:11	200.893	0.688 ± 0.001	05:5[illegible]
175.606	0.426 ± 0.001	06:13	200.880	0.718 ± 0.001	05:55	175.658	0.587 ± 0.001	06:14	175.478	0.623 ± 0.001	06:0[illegible]
150.376	0.381 ± 0.001	06:16	175.348	0.653 ± 0.001	06:01	150.335	0.528 ± 0.001	06:18	150.135	0.556 ± 0.001	06:0[illegible]
125.330	0.333 ± 0.001	06:20	149.983	0.590 ± 0.001	06:06	125.440	0.466 ± 0.001	06:22	125.512	0.485 ± 0.001	06:1[illegible]
100.351	0.283 ± 0.001	06:24	125.501	0.520 ± 0.001	06:11	100.386	0.398 ± 0.001	06:25	100.517	0.409 ± 0.001	06:1[illegible]
75.403	0.228 ± 0	06:28	100.570	0.443 ± 0.001	06:16	75.404	0.323 ± 0.001	06:29	75.499	0.327 ± 0.001	06:2[illegible]
50.336	0.170 ± 0	06:31	75.458	0.360 ± 0.001	06:21	50.488	0.240 ± 0	06:33	50.456	0.237 ± 0	06:2[illegible]
45.294	0.156 ± 0	06:35	50.503	0.266 ± 0.001	06:25	45.272	0.221 ± 0	06:37	45.362	0.219 ± 0	06:3[illegible]
40.239	0.143 ± 0	06:39	45.295	0.244 ± 0	06:30	40.265	0.202 ± 0	06:41	40.441	0.196 ± 0	06:3[illegible]
35.345	0.129 ± 0	06:43	40.255	0.221 ± 0	06:35	35.320	0.182 ± 0	06:45	35.315	0.176 ± 0	06:4[illegible]
30.291	0.115 ± 0	06:47	35.344	0.200 ± 0	06:40	30.349	0.161 ± 0	06:49	30.672	0.156 ± 0	06:4[illegible]
25.339	0.101 ± 0	06:51	30.575	0.175 ± 0	06:45	25.359	0.140 ± 0	06:53	25.420	0.132 ± 0	06:5[illegible]
20.334	0.086 ± 0	06:54	25.426	0.148 ± 0	06:50	20.370	0.118 ± 0	06:58	20.445	0.112 ± 0	06:5[illegible]
17.854	0.078 ± 0	06:58	20.367	0.124 ± 0	06:55	17.829	0.107 ± 0	07:02	17.918	0.099 ± 0	07:0[illegible]

15.351	0.069 ± 0	07:03	17.886	0.109 ± 0	07:00	15.350	0.094 ± 0	07:07	15.562	0.088 ± 0	07:08
13.858	0.064 ± 0	07:07	15.424	0.105 ± 0	07:06	13.885	0.086 ± 0	07:12	13.928	0.080 ± 0	07:14
12.840	0.053 ± 0	07:12	14.115	0.098 ± 0	07:11	12.801	0.075 ± 0	07:15	12.927	0.068 ± 0	07:18
11.441	0.042 ± 0	07:17	12.965	0.083 ± 0	07:15	11.426	0.062 ± 0	07:21	11.457	0.055 ± 0	07:23
10.084	0.043 ± 0	07:21	11.469	0.069 ± 0	07:19	10.137	0.061 ± 0	07:25	10.129	0.054 ± 0	07:28
8.138	0.036 ± 0	07:26	10.122	0.067 ± 0	07:24	8.250	0.051 ± 0	07:31	8.251	0.044 ± 0	07:34
6.023	0.028 ± 0	07:32	8.239	0.056 ± 0	07:30	6.076	0.039 ± 0	07:41	6.086	0.032 ± 0	07:48
4.025	0.020 ± 0	07:48	6.064	0.043 ± 0	07:36	4.044	0.027 ± 0	08:04	4.053	0.020 ± 0	08:18
2.047	0.010 ± 0	08:46	4.031	0.030 ± 0	07:49	2.052	0.014 ± 0	09:15	2.053	0.006 ± 0	10:12
1.032	0.002 ± 0	11:38	2.021	0.017 ± 0	08:20	1.024	0.006 ± 0	10:41			
			1.038	0.006 ± 0	10:55	0.506	0.002 ± 0	12:21			

Table S7. Water adsorption data on carbon materials at (a) 288.15 K, (b) 298.15 K and (c) 308.15 K

	BPL Carbon			C564			C569			C1005		
	Relative Pressure (P/P_0)	n (mmol/g)	Elapsed Time (h:min)	Relative Pressure (P/P_0)	n (mmol/g)	Elapsed Time (h:min)	Relative Pressure (P/P_0)	n (mmol/g)	Elapsed Time (h:min)	Relative Pressure (P/P_0)	n (mmol/g)	Elapse d Time (h:min)
288.15 K	0.005	0.01 ± 0	03:06	0.001	0.01 ± 0	03:19	0.001	0 ± 0	02:10	0.003	0 ± 0	02:07
	0.011	0.02 ± 0	03:29	0.003	0.01 ± 0	03:31	0.002	0.01 ± 0	02:53	0.005	0 ± 0	02:17
	0.015	0.03 ± 0	03:45	0.005	0.01 ± 0	03:48	0.005	0.02 ± 0	03:51	0.011	0 ± 0	02:34
	0.020	0.03 ± 0	03:58	0.010	0.02 ± 0	04:05	0.010	0.05 ± 0	04:39	0.015	0.01 ± 0	02:46
	0.025	0.04 ± 0	04:11	0.015	0.02 ± 0	04:18	0.015	0.07 ± 0	05:19	0.020	0.01 ± 0	02:58
	0.030	0.05 ± 0	04:24	0.020	0.02 ± 0	04:33	0.019	0.08 ± 0	05:48	0.025	0.01 ± 0	03:08
	0.040	0.06 ± 0	04:42	0.025	0.03 ± 0	04:46	0.025	0.10 ± 0	06:22	0.030	0.01 ± 0	03:20
	0.049	0.07 ± 0	04:57	0.029	0.03 ± 0	04:57	0.029	0.11 ± 0	06:52	0.039	0.01 ± 0	03:32
	0.058	0.08 ± 0	05:11	0.038	0.04 ± 0	05:11	0.039	0.14 ± 0	07:19	0.050	0.01 ± 0	03:45
	0.068	0.09 ± 0	05:25	0.049	0.04 ± 0	05:24	0.049	0.16 ± 0	07:48	0.058	0.02 ± 0	03:55
	0.079	0.11 ± 0	05:35	0.061	0.04 ± 0	05:38	0.061	0.19 ± 0	08:29	0.068	0.02 ± 0	04:05
	0.089	0.12 ± 0	05:45	0.069	0.05 ± 0	05:49	0.068	0.21 ± 0	08:53	0.080	0.02 ± 0	04:16
	0.099	0.14 ± 0	05:55	0.080	0.05 ± 0	06:00	0.077	0.23 ± 0	09:10	0.090	0.02 ± 0	04:25
	0.318	0.90 ± 0	06:12	0.090	0.06 ± 0	06:08	0.086	0.25 ± 0	09:27	0.100	0.02 ± 0	04:34
	0.476	3.37 ± 0.01	06:32	0.099	0.06 ± 0	06:18	0.099	0.27 ± 0	09:59	0.112	0.03 ± 0	04:42
	0.667	11.13 ± 0.02	07:28	0.111	0.07 ± 0	06:28	0.110	0.30 ± 0	10:19	0.125	0.03 ± 0	04:50
	0.861	20.64 ± 0.04	08:46	0.124	0.09 ± 0	06:37	0.122	0.32 ± 0	10:40	0.147	0.04 ± 0	05:01
	0.910	21.62 ± 0.04	09:05	0.146	0.1 ± 0	06:49	0.147	0.37 ± 0	11:00	0.174	0.04 ± 0	05:12
	0.729	20.31 ± 0.04	09:17	0.172	0.15 ± 0	07:01	0.170	0.41 ± 0	11:17	0.199	0.05 ± 0	05:21
	0.525	10.64 ± 0.02	11:40	0.197	0.55 ± 0	07:10	0.194	0.45 ± 0	11:32	0.292	0.07 ± 0	05:34
	0.313	1.17 ± 0	13:05	0.289	6.59 ± 0.01	07:22	0.294	0.62 ± 0	12:02	0.476	0.3 ± 0	05:43
	0.093	0.32 ± 0	14:34	0.476	7.59 ± 0.02	07:33	0.479	1.10 ± 0	12:17	0.668	11.75 ± 0.02	07:03
	0.083	0.29 ± 0	15:04	0.684	7.80 ± 0.02	08:48	0.686	4.69 ± 0.01	13:04	0.856	14.93 ± 0.03	07:32
	0.063	0.26 ± 0	15:57	0.859	7.13 ± 0.01	09:01	0.867	6.32 ± 0.01	13:23	0.909	15.15 ± 0.03	07:41
	0.039	0.21 ± 0	18:02	0.922	6.98 ± 0.01	09:08	0.908	7.04 ± 0.01	13:39	0.714	14.48 ± 0.03	07:48
	0.026	0.18 ± 0	20:25	0.740	3.26 ± 0.01	09:21	0.725	5.35 ± 0.01	13:56	0.524	7.37 ± 0.01	09:26
	0.010	0.15 ± 0	23:33	0.733	0.26 ± 0	09:30	0.524	2.97 ± 0.01	14:44	0.311	0.17 ± 0	11:07
	0.005	0.10 ± 0	43:08	0.525	0.14 ± 0	10:39	0.308	0.87 ± 0	15:28	0.090	0.08 ± 0	13:46
				0.309	0.13 ± 0	11:27	0.100	0.50 ± 0	16:27	0.082	0.08 ± 0	14:31
				0.093	0.11 ± 0	13:10	0.082	0.45 ± 0	17:07	0.057	0.06 ± 0	16:53
				0.081	0.1 ± 0	13:46	0.059	0.39 ± 0	18:01	0.040	0.05 ± 0	19:27
				0.058	0.09 ± 0	14:54	0.042	0.34 ± 0	19:04	0.026	0.04 ± 0	24:50
				0.041	0.07 ± 0	16:31	0.041	0.33 ± 0	19:27	0.010	0.02 ± 0	33:27

				0.025	0.03 ± 0	18:16	0.025	0.28 ± 0	20:53			
				0.010	0.01 ± 0	20:55	0.010	0.21 ± 0	24:16			
				0.005	0.01 ± 0	59:31	0.005	0.17 ± 0	32:46			
298.15 K	0.005	0.02 ± 0	03:38	0.001	0 ± 0	01:52	0.002	0 ± 0	02:12	0.001	0 ± 0	01:56
	0.010	0.03 ± 0	04:11	0.001	0 ± 0	02:00	0.005	0.02 ± 0	02:49	0.002	0 ± 0	02:04
	0.016	0.05 ± 0	04:53	0.003	0 ± 0	02:14	0.010	0.04 ± 0	03:18	0.005	0 ± 0	02:13
	0.020	0.06 ± 0	05:21	0.005	0.01 ± 0	02:28	0.016	0.06 ± 0	03:47	0.010	0 ± 0	02:27
	0.025	0.07 ± 0	05:46	0.010	0.01 ± 0	02:45	0.019	0.07 ± 0	04:05	0.015	0.01 ± 0	02:45
	0.030	0.08 ± 0	06:12	0.015	0.01 ± 0	03:00	0.024	0.09 ± 0	04:22	0.020	0.01 ± 0	03:01
	0.041	0.11 ± 0	06:47	0.020	0.02 ± 0	03:13	0.030	0.10 ± 0	04:42	0.025	0.01 ± 0	03:16
	0.050	0.13 ± 0	07:08	0.025	0.02 ± 0	03:25	0.040	0.13 ± 0	05:02	0.030	0.01 ± 0	03:29
	0.061	0.16 ± 0	07:36	0.030	0.02 ± 0	03:37	0.050	0.16 ± 0	05:22	0.039	0.01 ± 0	03:42
	0.070	0.18 ± 0	07:55	0.039	0.03 ± 0	03:51	0.057	0.17 ± 0	05:35	0.049	0.02 ± 0	03:57
	0.079	0.20 ± 0	08:10	0.053	0.04 ± 0	04:10	0.067	0.20 ± 0	05:50	0.058	0.02 ± 0	04:10
	0.089	0.22 ± 0	08:22	0.059	0.04 ± 0	04:19	0.077	0.22 ± 0	06:05	0.068	0.02 ± 0	04:25
	0.099	0.25 ± 0	08:38	0.070	0.04 ± 0	04:31	0.088	0.24 ± 0	06:16	0.080	0.03 ± 0	04:37
	0.310	1.08 ± 0	08:56	0.080	0.05 ± 0	04:41	0.098	0.26 ± 0	06:46	0.090	0.03 ± 0	04:48
	0.483	3.31 ± 0.01	09:14	0.090	0.05 ± 0	04:50	0.109	0.29 ± 0	07:11	0.100	0.03 ± 0	04:58
	0.668	11.32 ± 0.04	10:18	0.100	0.06 ± 0	04:59	0.121	0.32 ± 0	07:39	0.112	0.04 ± 0	05:07
	0.857	18.84 ± 0.06	11:37	0.112	0.06 ± 0	05:08	0.147	0.37 ± 0	08:20	0.125	0.04 ± 0	05:16
	0.903	19.93 ± 0.07	12:15	0.124	0.07 ± 0	05:16	0.171	0.42 ± 0	08:38	0.148	0.05 ± 0	05:24
	0.735	18.65 ± 0.06	12:22	0.146	0.08 ± 0	05:27	0.196	0.47 ± 0	08:54	0.174	0.06 ± 0	05:35
	0.531	10.93 ± 0.04	12:50	0.172	0.09 ± 0	05:38	0.298	0.66 ± 0	09:32	0.199	0.07 ± 0	05:44
	0.288	2.11 ± 0.01	13:33	0.197	0.11 ± 0	05:47	0.484	1.47 ± 0	10:15	0.287	0.10 ± 0	05:53
	0.090	1.30 ± 0	15:32	0.293	0.16 ± 0	06:02	0.671	4.74 ± 0.01	12:21	0.476	0.44 ± 0	06:06
	0.084	1.28 ± 0	15:52	0.486	0.64 ± 0	06:16	0.858	6.02 ± 0.01	13:35	0.665	11.19 ± 0.02	06:13
	0.059	1.20 ± 0	17:18	0.674	6.51 ± 0.01	09:11	0.903	6.43 ± 0.01	15:35	0.857	15.20 ± 0.03	09:49
	0.041	1.11 ± 0	19:52	0.858	7.68 ± 0.02	10:14	0.680	5.37 ± 0.01	16:09	0.903	15.45 ± 0.03	13:11
	0.024	1.04 ± 0	23:54	0.903	7.91 ± 0.02	11:40	0.530	3.41 ± 0.01	16:41	0.709	14.81 ± 0.03	14:06
	0.010	0.95 ± 0	31:39	0.687	7.01 ± 0.01	12:19	0.299	1.12 ± 0	17:36	0.534	5.84 ± 0.01	14:25
	0.005	0.92 ± 0	36:06	0.533	2.62 ± 0.01	14:12	0.099	0.73 ± 0	18:48	0.263	0.85 ± 0	16:49
				0.327	0.64 ± 0	15:18	0.080	0.66 ± 0	19:39	0.100	0.76 ± 0	18:21
				0.308	0.63 ± 0	15:37	0.062	0.61 ± 0	20:33	0.082	0.74 ± 0	21:00
				0.100	0.49 ± 0	18:30	0.039	0.54 ± 0	21:49	0.062	0.72 ± 0	22:27
				0.084	0.47 ± 0	19:30	0.026	0.49 ± 0	23:25	0.041	0.69 ± 0	24:02
				0.058	0.45 ± 0	21:57	0.026	0.49 ± 0	23:58	0.025	0.67 ± 0	28:06
				0.042	0.43 ± 0	24:16	0.010	0.43 ± 0	26:32	0.010	0.63 ± 0	34:15
				0.026	0.4 ± 0	29:26	0.005	0.38 ± 0	31:39	0.004	0.63 ± 0	51:30
				0.010	0.38 ± 0	36:23						

				0.005	0.37 ± 0	44:11						
308.15 K	0.001	0.002 ± 0	01:58	0.001	0.001 ± 0	03:09	0.001	0.002 ± 0	02:12	0.003	0.001 ± 0	03:02
	0.001	0.003 ± 0	02:05	0.001	0.002 ± 0	03:23	0.001	0.004 ± 0	02:33	0.005	0.002 ± 0	03:08
	0.003	0.007 ± 0	02:14	0.003	0.004 ± 0	03:39	0.003	0.010 ± 0	03:01	0.010	0.006 ± 0	03:14
	0.005	0.014 ± 0	02:27	0.005	0.007 ± 0	03:58	0.005	0.019 ± 0	03:26	0.015	0.009 ± 0	03:18
	0.010	0.026 ± 0	02:43	0.010	0.013 ± 0	04:20	0.010	0.037 ± 0	04:01	0.020	0.011 ± 0	03:22
	0.015	0.036 ± 0	02:57	0.015	0.018 ± 0	04:38	0.015	0.047 ± 0	04:26	0.025	0.014 ± 0	03:26
	0.020	0.046 ± 0	03:10	0.020	0.022 ± 0	04:56	0.019	0.057 ± 0	04:42	0.030	0.016 ± 0	03:30
	0.025	0.055 ± 0	03:23	0.025	0.026 ± 0	05:09	0.024	0.068 ± 0	04:58	0.039	0.021 ± 0	03:35
	0.030	0.065 ± 0	03:33	0.030	0.030 ± 0	05:21	0.029	0.078 ± 0	05:12	0.050	0.026 ± 0	03:40
	0.038	0.079 ± 0	03:47	0.039	0.037 ± 0	05:35	0.040	0.098 ± 0	05:29	0.059	0.031 ± 0	03:45
	0.048	0.098 ± 0	04:00	0.049	0.045 ± 0	05:50	0.049	0.115 ± 0	05:45	0.068	0.035 ± 0	03:49
	0.061	0.119 ± 0	04:14	0.058	0.051 ± 0	06:01	0.059	0.131 ± 0	05:57	0.080	0.041 ± 0	03:53
	0.067	0.130 ± 0	04:22	0.068	0.058 ± 0	06:11	0.067	0.145 ± 0	06:11	0.090	0.046 ± 0	03:57
	0.079	0.152 ± 0	04:33	0.080	0.066 ± 0	06:21	0.078	0.162 ± 0	06:24	0.100	0.051 ± 0	04:01
	0.090	0.170 ± 0	04:42	0.090	0.072 ± 0	06:30	0.089	0.178 ± 0	06:34	0.112	0.057 ± 0	04:05
	0.099	0.188 ± 0	04:51	0.100	0.079 ± 0	06:37	0.099	0.193 ± 0	06:47	0.125	0.064 ± 0	04:09
	0.111	0.210 ± 0	05:00	0.112	0.087 ± 0	06:44	0.111	0.210 ± 0	06:59	0.148	0.076 ± 0	04:15
	0.124	0.236 ± 0.001	05:09	0.125	0.095 ± 0	06:51	0.123	0.227 ± 0	07:08	0.177	0.089 ± 0	04:21
	0.144	0.278 ± 0.001	05:18	0.148	0.110 ± 0	07:00	0.144	0.254 ± 0	07:19	0.202	0.103 ± 0	04:27
	0.171	0.336 ± 0.001	05:28	0.177	0.126 ± 0	07:09	0.171	0.288 ± 0.001	07:32	0.300	0.187 ± 0	04:36
	0.197	0.403 ± 0.001	05:37	0.202	0.142 ± 0	07:17	0.199	0.321 ± 0.001	07:43	0.102	0.068 ± 0	04:56
	0.287	0.843 ± 0.002	05:47	0.303	0.239 ± 0	07:26	0.303	0.471 ± 0.001	07:58	0.080	0.051 ± 0	05:22
	0.101	0.255 ± 0.001	06:07	0.104	0.115 ± 0	07:39	0.104	0.249 ± 0	08:16	0.062	0.035 ± 0	06:03
	0.084	0.224 ± 0	06:17	0.078	0.101 ± 0	07:50	0.079	0.215 ± 0	08:29	0.039	0.003 ± 0	08:53
	0.062	0.185 ± 0	06:33	0.061	0.090 ± 0	08:01	0.061	0.191 ± 0	08:45			
	0.041	0.150 ± 0	07:04	0.041	0.080 ± 0	08:26	0.040	0.157 ± 0	09:12			
	0.026	0.119 ± 0	08:03	0.025	0.068 ± 0	09:20	0.026	0.131 ± 0	09:48			
	0.010	0.068 ± 0	13:27	0.010	0.050 ± 0	12:32	0.011	0.090 ± 0	11:53			
	0.005	0.047 ± 0	17:28	0.005	0.037 ± 0	17:16	0.005	0.069 ± 0	14:31			

Table S8. Acetone adsorption data on carbon materials at (a) 288.15 K, (b) 298.15 K and (c) 308.15 K

	BPL Carbon			C564			C569			C1005		
	Relative Pressure (P/P_0)	n (mmol/g)	Elapsed Time (h:min)	Relative Pressure (P/P_0)	n (mmol/g)	Elapsed Time (h:min)	Relative Pressure (P/P_0)	n (mmol/g)	Elapsed Time (h:min)	Relative Pressure (P/P_0)	n (mmol/g)	Elapsed Time (h:min)
288.15 K	0.001	0.15 ± 0	03:11	0.001	0.2 ± 0	06:08	0.001	0.39 ± 0	07:12	0.001	0.15 ± 0	06:30
	0.001	0.34 ± 0	03:34	0.001	0.58 ± 0	07:50	0.001	0.76 ± 0	08:30	0.001	0.33 ± 0	06:58
	0.002	0.75 ± 0	04:16	0.003	1.17 ± 0	08:53	0.003	1.15 ± 0	09:33	0.002	0.80 ± 0	07:41
	0.005	1.47 ± 0	04:51	0.005	1.60 ± 0	09:34	0.005	1.39 ± 0	10:40	0.005	1.71 ± 0	08:21
	0.010	2.27 ± 0.01	05:16	0.012	1.86 ± 0	10:21	0.010	1.56 ± 0	11:49	0.011	2.81 ± 0.01	08:53
	0.017	3.04 ± 0.01	05:59	0.015	1.92 ± 0	10:41	0.015	1.67 ± 0	12:44	0.015	3.08 ± 0.01	09:21
	0.020	3.27 ± 0.01	06:23	0.020	1.99 ± 0	11:03	0.020	1.73 ± 0	13:05	0.020	3.30 ± 0.01	09:42
	0.024	3.55 ± 0.01	06:40	0.024	2.02 ± 0	11:13	0.025	1.79 ± 0	13:31	0.024	3.43 ± 0.01	09:59
	0.029	3.79 ± 0.01	06:52	0.030	2.06 ± 0	11:23	0.030	1.83 ± 0	13:51	0.030	3.56 ± 0.01	10:09
	0.038	4.12 ± 0.01	07:02	0.040	2.12 ± 0	11:32	0.040	1.88 ± 0	14:09	0.039	3.70 ± 0.01	10:17
	0.049	4.41 ± 0.01	07:11	0.048	2.15 ± 0	11:38	0.053	1.94 ± 0	14:29	0.051	3.81 ± 0.01	10:24
	0.061	4.66 ± 0.01	07:18	0.058	2.19 ± 0	11:44	0.060	1.97 ± 0	14:38	0.059	3.88 ± 0.01	10:28
	0.071	4.89 ± 0.01	07:24	0.068	2.24 ± 0.01	11:49	0.069	2.03 ± 0	14:47	0.069	3.97 ± 0.01	10:32
	0.082	5.05 ± 0.01	07:27	0.082	2.28 ± 0.01	11:53	0.081	2.06 ± 0	14:56	0.081	4.03 ± 0.01	10:36
	0.092	5.17 ± 0.01	07:31	0.092	2.03 ± 0.01	11:59	0.091	2.09 ± 0	15:02	0.091	4.07 ± 0.01	10:39
	0.103	5.27 ± 0.01	07:34	0.101	2.33 ± 0.01	12:07	0.101	2.12 ± 0	15:09	0.102	4.12 ± 0.01	10:43
	0.115	5.38 ± 0.01	07:38	0.113	2.35 ± 0.01	12:11	0.112	2.15 ± 0	15:16	0.113	4.16 ± 0.01	10:51
	0.127	5.48 ± 0.01	07:41	0.125	2.38 ± 0.01	12:14	0.125	2.18 ± 0	15:23	0.126	4.19 ± 0.01	10:57
	0.150	5.62 ± 0.01	07:44	0.149	2.42 ± 0.01	12:18	0.149	2.23 ± 0	15:29	0.149	4.25 ± 0.01	11:00
	0.177	5.76 ± 0.01	07:48	0.176	2.47 ± 0.01	12:22	0.175	2.28 ± 0	15:36	0.175	4.31 ± 0.01	11:04
	0.203	5.87 ± 0.01	07:51	0.201	2.51 ± 0.01	12:25	0.200	2.33 ± 0	15:42	0.201	4.36 ± 0.01	11:09
	0.294	6.15 ± 0.01	07:56	0.293	2.63 ± 0.01	12:33	0.290	2.48 ± 0.01	15:56	0.291	4.52 ± 0.01	11:17
	0.490	6.79 ± 0.02	08:17	0.482	2.93 ± 0.01	12:51	0.497	2.91 ± 0.01	16:31	0.497	5.02 ± 0.01	11:52
	0.687	8.03 ± 0.02	09:08	0.690	3.75 ± 0.01	13:38	0.697	3.75 ± 0.01	17:25	0.666	5.93 ± 0.01	12:45
	0.868	10.49 ± 0.02	10:30	0.882	5.41 ± 0.01	13:59	0.889	5.37 ± 0.01	17:48	0.887	8.43 ± 0.02	13:13
	0.971	11.71 ± 0.03	10:34	0.936	7.99 ± 0.02	14:11	0.918	6.31 ± 0.01	17:53	0.929	11.36 ± 0.03	13:28
	0.932	11.07 ± 0.02	10:37	0.921	7.81 ± 0.02	14:16	0.640	4.20 ± 0.01	18:02	0.617	6.83 ± 0.02	13:47
	0.695	10.29 ± 0.02	10:48	0.694	4.63 ± 0.01	14:25	0.488	3.90 ± 0.01	18:18	0.509	6.52 ± 0.01	14:05
	0.503	9.66 ± 0.02	11:11	0.468	4.13 ± 0.01	14:43	0.312	3.58 ± 0.01	18:43	0.305	5.96 ± 0.01	14:57
	0.298	8.74 ± 0.02	11:43	0.308	3.82 ± 0.01	15:11	0.103	3.08 ± 0.01	20:11	0.105	5.24 ± 0.01	17:09
	0.102	7.68 ± 0.02	12:10	0.101	3.36 ± 0.01	16:14	0.082	2.98 ± 0.01	20:53	0.086	5.12 ± 0.01	17:40
	0.085	7.41 ± 0.02	12:28	0.084	3.31 ± 0.01	16:19	0.062	2.83 ± 0.01	22:16	0.061	4.87 ± 0.01	19:14

	0.059	6.69 ± 0.01	14:06	0.060	3.02 ± 0.01	19:14	0.038	2.67 ± 0.01	23:47	0.040	4.63 ± 0.01	20:13
	0.037	6.07 ± 0.01	15:03	0.038	2.85 ± 0.01	21:28				0.026	4.37 ± 0.01	21:27
	0.026	5.62 ± 0.01	15:49	0.025	2.71 ± 0.01	23:23				0.010	3.77 ± 0.01	23:23
	0.010	4.54 ± 0.01	17:39							0.005	3.29 ± 0.01	24:41
	0.005	3.84 ± 0.01	19:37									
298.15 K	0.001	0.41 ± 0	07:42	0.001	0.34 ± 0	08:21	0.001	0.33 ± 0	07:38	0.001	0.05 ± 0	05:11
	0.001	0.82 ± 0	08:35	0.001	0.65 ± 0	09:08	0.001	0.65 ± 0	08:39	0.001	0.13 ± 0	06:53
	0.002	1.19 ± 0	08:59	0.003	1.28 ± 0	10:13	0.003	1.03 ± 0	09:23	0.003	0.27 ± 0	07:37
	0.005	1.76 ± 0	09:24	0.005	1.52 ± 0	10:36	0.005	1.31 ± 0	10:22	0.005	0.27 ± 0	07:58
	0.010	2.41 ± 0.01	09:47	0.011	1.80 ± 0	11:17	0.011	1.52 ± 0	11:15	0.014	1.16 ± 0	69:18
	0.016	2.95 ± 0.01	10:06	0.016	1.90 ± 0	11:42	0.016	1.62 ± 0	11:45	0.019	2.18 ± 0	70:12
	0.020	3.21 ± 0.01	10:21	0.020	1.95 ± 0	11:55	0.019	1.67 ± 0	12:05	0.024	2.55 ± 0.01	70:56
	0.024	3.43 ± 0.01	10:33	0.024	2.00 ± 0	12:06	0.025	1.73 ± 0	12:24	0.029	2.76 ± 0.01	71:31
	0.029	3.66 ± 0.01	10:41	0.030	2.04 ± 0	12:13	0.029	1.76 ± 0	12:36	0.040	3.03 ± 0.01	71:50
	0.042	4.07 ± 0.01	10:48	0.040	2.10 ± 0	12:20	0.040	1.83 ± 0	12:51	0.048	3.18 ± 0.01	72:05
	0.048	4.28 ± 0.01	10:54	0.052	2.16 ± 0	12:27	0.052	1.91 ± 0	13:02	0.061	3.31 ± 0.01	72:15
	0.059	4.51 ± 0.01	10:58	0.060	2.19 ± 0	12:31	0.059	1.95 ± 0	13:11	0.068	3.37 ± 0.01	72:25
	0.071	4.72 ± 0.01	11:02	0.070	2.22 ± 0.01	12:35	0.070	1.99 ± 0	13:20	0.079	3.44 ± 0.01	72:31
	0.080	4.84 ± 0.01	11:05	0.080	2.25 ± 0.01	12:39	0.080	2.03 ± 0	13:28	0.091	3.50 ± 0.01	72:37
	0.090	4.97 ± 0.01	11:07	0.090	2.28 ± 0.01	12:43	0.090	2.06 ± 0	13:34	0.101	3.55 ± 0.01	72:41
	0.101	5.08 ± 0.01	11:10	0.101	2.31 ± 0.01	12:46	0.100	2.10 ± 0	13:42	0.113	3.60 ± 0.01	72:50
	0.113	5.20 ± 0.01	11:12	0.113	2.34 ± 0.01	12:50	0.112	2.14 ± 0	13:49	0.126	3.64 ± 0.01	72:54
	0.126	5.31 ± 0.01	11:14	0.125	2.37 ± 0.01	12:53	0.125	2.17 ± 0	13:56	0.149	3.71 ± 0.01	72:59
	0.148	5.46 ± 0.01	11:17	0.149	2.41 ± 0.01	12:58	0.148	2.23 ± 0	14:05	0.175	3.78 ± 0.01	73:04
	0.176	5.61 ± 0.01	11:21	0.175	2.47 ± 0.01	13:03	0.174	2.30 ± 0	14:16	0.200	3.85 ± 0.01	73:09
	0.201	5.73 ± 0.01	11:26	0.201	2.52 ± 0.01	13:10	0.199	2.37 ± 0	14:26	0.288	4.09 ± 0.01	73:26
	0.298	6.11 ± 0.01	11:37	0.291	2.72 ± 0.01	13:25	0.298	2.73 ± 0.01	15:04	0.492	5.52 ± 0.01	74:44
	0.487	7.42 ± 0.02	12:33	0.494	3.84 ± 0.01	14:27	0.485	4.33 ± 0.01	16:33	0.683	8.49 ± 0.02	76:19
	0.690	10.46 ± 0.02	14:06	0.681	6.53 ± 0.01	15:58	0.690	7.72 ± 0.01	18:25	0.506	7.82 ± 0.01	76:39
	0.499	9.83 ± 0.02	14:28	0.516	5.99 ± 0.01	16:15	0.516	7.03 ± 0.01	18:48	0.314	6.86 ± 0.01	77:52
	0.302	8.78 ± 0.02	15:23	0.312	5.19 ± 0.01	17:09	0.307	5.87 ± 0.01	20:21	0.105	5.73 ± 0.01	81:06
	0.096	7.48 ± 0.02	16:25	0.104	4.32 ± 0.01	19:55	0.100	4.54 ± 0.01	25:21	0.080	5.51 ± 0.01	82:12
	0.084	7.32 ± 0.02	16:31	0.080	4.22 ± 0.01	20:22	0.080	4.36 ± 0.01	26:41	0.062	5.32 ± 0.01	83:09
	0.062	6.97 ± 0.02	16:40	0.062	4.07 ± 0.01	21:21				0.042	5.09 ± 0.01	83:41
	0.041	6.47 ± 0.01	17:00	0.042	3.89 ± 0.01	22:30				0.025	4.64 ± 0.01	86:21
	0.025	5.52 ± 0.01	19:44	0.025	3.61 ± 0.01	25:43				0.010	4.00 ± 0.01	88:34
	0.010	4.48 ± 0.01	21:18							0.005	3.42 ± 0.01	90:45
	0.005	3.76 ± 0.01	23:00									

308.15 K	0.001	0.48 ± 0	06:41	0.001	0.53 ± 0	07:56	0.001	0.13 ± 0	03:09	0.005	0.26 ± 0	26:53
	0.001	0.73 ± 0	07:01	0.001	0.81 ± 0	08:28	0.001	0.16 ± 0	03:19	0.010	0.60 ± 0	27:28
	0.003	1.10 ± 0	07:27	0.003	1.22 ± 0	09:07	0.002	0.20 ± 0	03:38	0.014	0.91 ± 0	28:24
	0.005	1.59 ± 0	07:52	0.005	1.46 ± 0	09:35	0.005	0.27 ± 0	03:59	0.019	1.12 ± 0	29:03
	0.011	2.17 ± 0.01	08:16	0.011	1.69 ± 0	10:06	0.011	0.36 ± 0	04:37	0.024	1.24 ± 0	29:41
	0.016	2.55 ± 0.01	08:40	0.016	1.78 ± 0	10:32	0.014	0.39 ± 0	05:00	0.029	1.40 ± 0	30:27
	0.020	2.82 ± 0.01	09:03	0.020	1.84 ± 0	10:49	0.019	0.43 ± 0	05:28	0.039	1.67 ± 0	31:27
	0.025	3.00 ± 0.01	09:17	0.025	1.88 ± 0	11:00	0.024	0.47 ± 0	05:57	0.051	1.94 ± 0.01	32:52
	0.030	3.26 ± 0.01	09:36	0.029	1.94 ± 0	11:09	0.029	0.52 ± 0	06:22	0.059	2.11 ± 0.01	33:48
	0.039	3.53 ± 0.01	09:49	0.039	2.00 ± 0	11:17	0.038	0.58 ± 0	06:57	0.068	2.27 ± 0.01	34:33
	0.049	3.77 ± 0.01	10:01	0.052	2.06 ± 0	11:26	0.052	0.67 ± 0	07:41	0.078	2.42 ± 0.01	35:13
	0.059	3.99 ± 0.01	10:12	0.060	2.09 ± 0	11:32	0.058	0.71 ± 0	08:05	0.089	2.55 ± 0.01	35:47
	0.067	4.11 ± 0.01	10:18	0.070	2.12 ± 0	11:36	0.069	0.77 ± 0	08:34	0.099	2.66 ± 0.01	36:17
	0.079	4.28 ± 0.01	10:24	0.079	2.24 ± 0	22:15	0.079	0.83 ± 0	09:00	0.111	2.78 ± 0.01	36:45
	0.090	4.41 ± 0.01	10:30	0.090	2.27 ± 0	22:18	0.089	0.88 ± 0	09:21	0.124	2.88 ± 0.01	37:08
	0.100	4.52 ± 0.01	10:36	0.101	2.29 ± 0	22:21	0.100	0.93 ± 0	09:38	0.147	3.02 ± 0.01	37:30
	0.112	4.64 ± 0.01	10:41	0.113	2.31 ± 0	22:23	0.112	0.98 ± 0	09:55	0.173	3.16 ± 0.01	37:48
	0.125	4.75 ± 0.01	10:46	0.125	2.33 ± 0.01	22:26	0.124	1.03 ± 0	10:11	0.200	3.27 ± 0.01	38:01
	0.148	4.92 ± 0.01	10:52	0.149	2.38 ± 0.01	22:29	0.147	1.13 ± 0	10:34	0.294	3.58 ± 0.01	38:14
	0.175	5.08 ± 0.01	10:57	0.174	2.43 ± 0.01	22:35	0.174	1.22 ± 0	10:51	0.733	4.61 ± 0.01	93:15
	0.201	5.23 ± 0.01	11:04	0.200	2.49 ± 0.01	22:41	0.199	1.29 ± 0	11:03	0.931	4.90 ± 0.01	93:22
	0.298	5.90 ± 0.01	11:35	0.287	2.82 ± 0.01	23:05	0.293	1.51 ± 0	11:18	0.957	4.96 ± 0.01	93:24
	0.500	7.86 ± 0.02	22:47	0.587	3.97 ± 0.01	46:04	0.487	1.86 ± 0	11:29	0.879	4.97 ± 0.01	93:25
	0.832	10.03 ± 0.02	46:04	0.748	5.12 ± 0.01	47:59	0.687	2.13 ± 0.01	11:38	0.702	4.94 ± 0.01	93:28
	0.890	11.44 ± 0.03	47:57	0.888	5.60 ± 0.01	48:19	0.934	3.01 ± 0.01	21:55	0.503	4.85 ± 0.01	93:31
	0.936	11.64 ± 0.03	48:06	0.955	5.94 ± 0.01	48:32	1.006	3.15 ± 0.01	22:06	0.309	4.70 ± 0.01	93:37
	0.843	11.67 ± 0.03	48:11	0.879	5.92 ± 0.01	48:34	0.901	3.14 ± 0.01	22:08	0.099	4.23 ± 0.01	94:12
	0.708	11.48 ± 0.03	48:16	0.706	5.81 ± 0.01	48:37	0.701	3.13 ± 0.01	22:09	0.083	4.14 ± 0.01	94:26
	0.513	10.75 ± 0.02	48:36	0.478	5.42 ± 0.01	48:55	0.503	3.09 ± 0.01	22:12	0.061	3.99 ± 0.01	94:47
	0.292	9.53 ± 0.02	49:41	0.302	4.95 ± 0.01	49:35	0.305	3.02 ± 0.01	22:18	0.041	3.79 ± 0.01	95:15
	0.102	8.17 ± 0.02	51:06	0.104	4.20 ± 0.01	51:46	0.105	2.81 ± 0.01	22:39	0.026	3.55 ± 0.01	95:38
	0.082	7.82 ± 0.02	51:41	0.084	4.08 ± 0.01	52:25	0.082	2.75 ± 0.01	22:53	0.010	3.01 ± 0.01	96:11
	0.057	7.42 ± 0.02	51:59	0.062	3.86 ± 0.01	54:01	0.061	2.67 ± 0.01	23:13			
	0.043	7.09 ± 0.02	52:12	0.038	3.70 ± 0.01	54:54	0.042	2.58 ± 0.01	23:35			
	0.026	6.26 ± 0.01	53:56	0.024	3.49 ± 0.01	56:52	0.026	2.47 ± 0.01	24:07			
	0.010	5.23 ± 0.01	55:09	0.010	3.25 ± 0.01	58:02	0.010	2.28 ± 0.01	25:28			
	0.005	4.55 ± 0.01	56:08	0.005	3.04 ± 0.01	59:05						

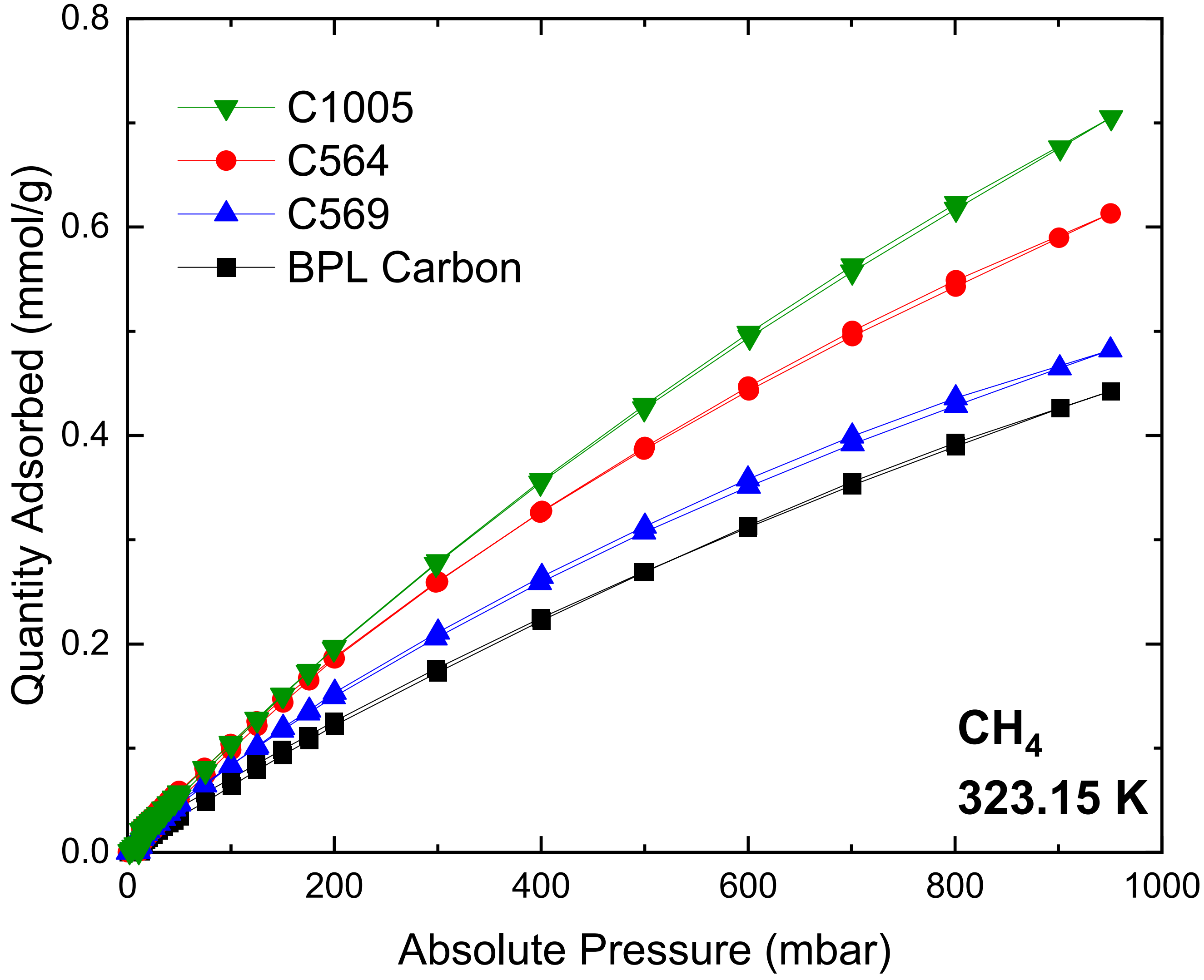

Figure S9. CH_4 isotherms on BPL Carbon, C564, C569, and C1005 at 323.15 K. Adsorption and desorption data overlap.

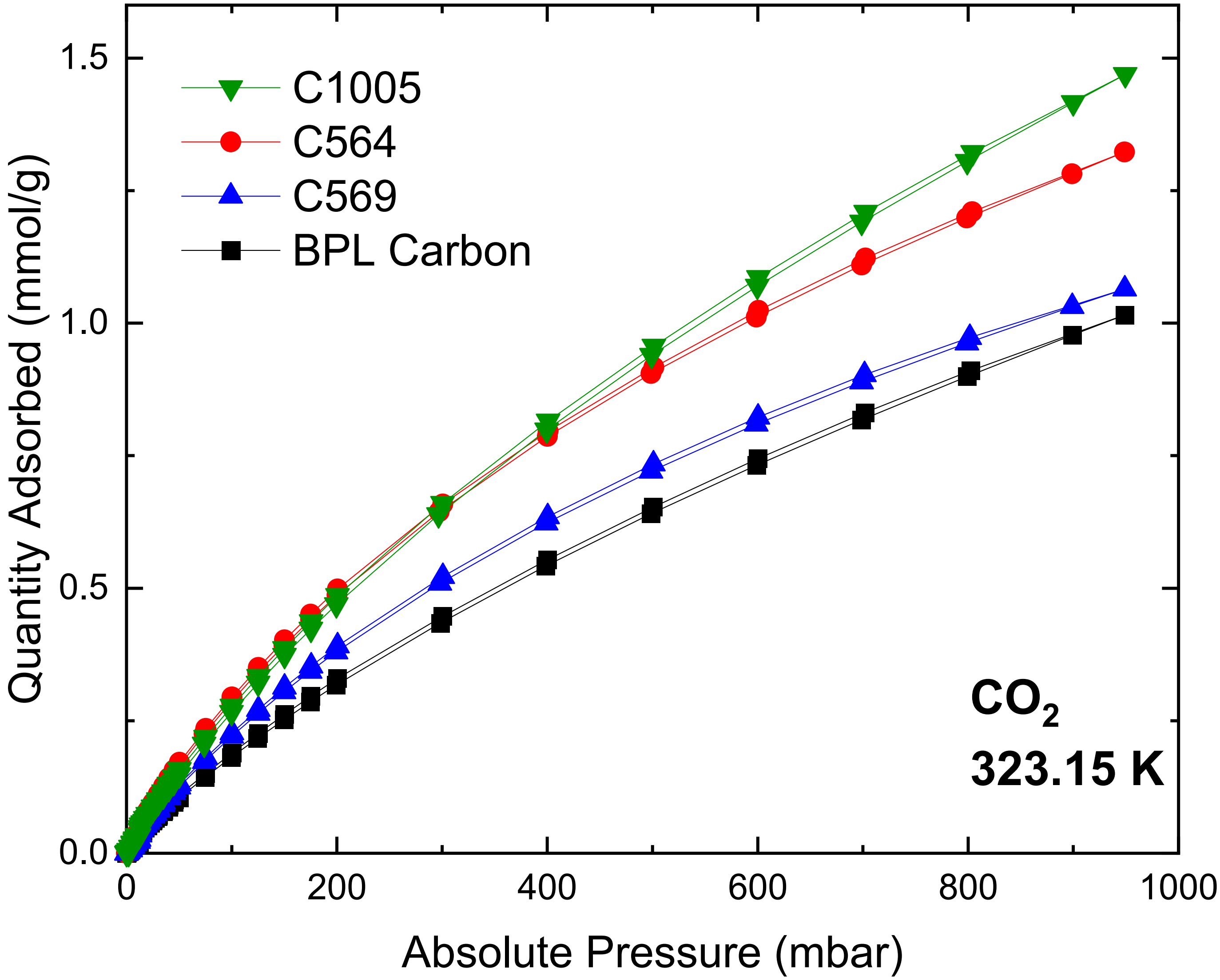

Figure S10. CO_2 isotherms on BPL Carbon, C564, C569, and C1005 at 323.15 K. Adsorption and desorption data overlap.

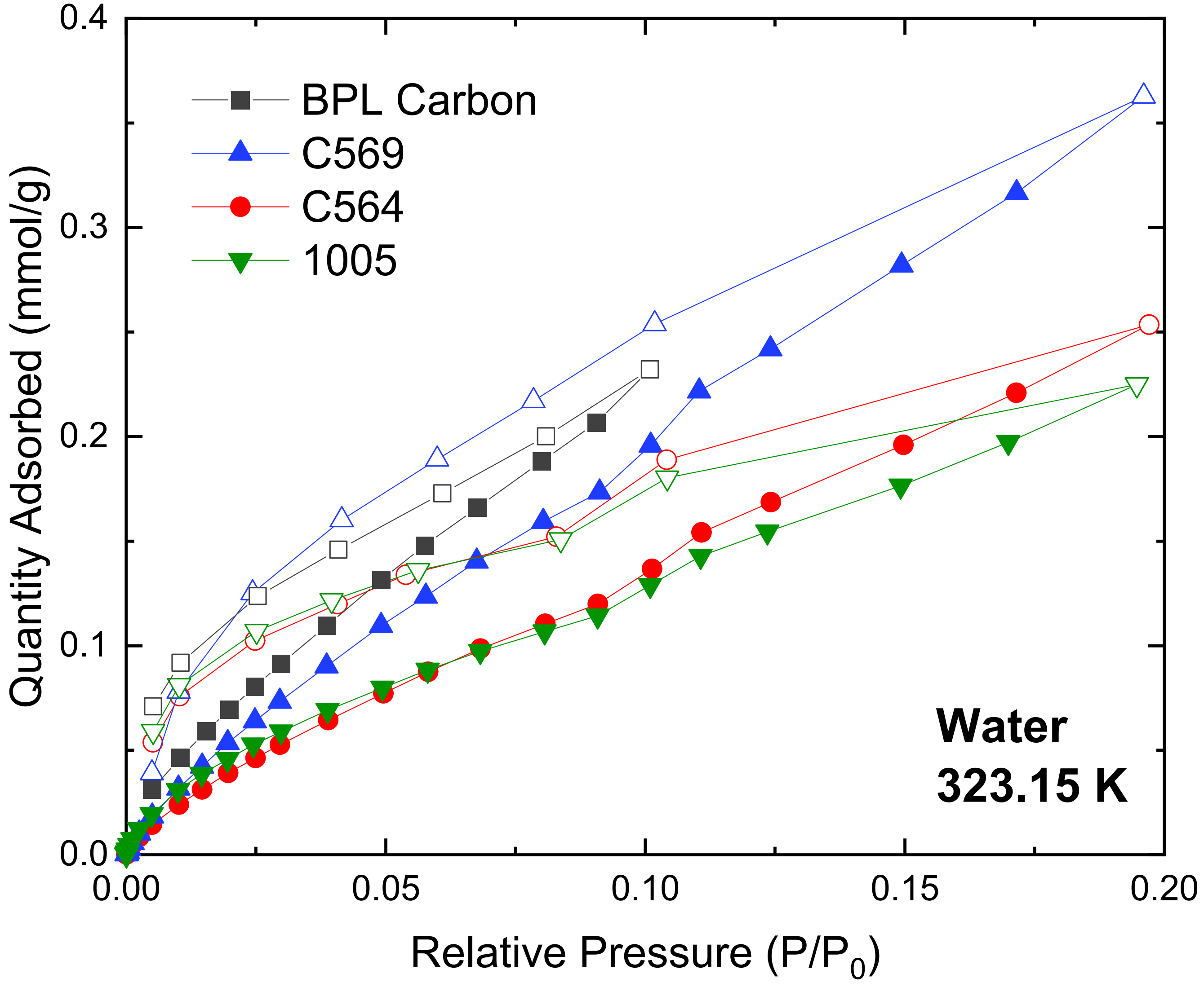

Figure S11. Water isotherms on BPL Carbon, C564, C569, and C1005 at 323.15 K. Closed symbols represent adsorption data, open symbols represent desorption data.

Table S9. CO_2 adsorption data on carbon materials at 323.15 K

BPL Carbon			C564			C569			C1005		
Absolute Pressure (mbar)	n (mmol/g)	Elapsed Time (h:min)	Absolute Pressure (mbar)	n (mmol/g)	Elapsed Time (h:min)	Absolute Pressure (mbar)	n (mmol/g)	Elapsed Time (h:min)	Absolute Pressure (mbar)	n (mmol/g)	Elapsed Time (h:min)
0.247	6.42±0E-05	03:52	0.122	2.2±0E-04	03:44	0.246	1.4±0E-04	03:51	0.247	2.2±0E-04	02:24
0.494	0.001 ± 0	03:56	0.245	0.001 ± 0	03:49	0.489	0.001 ± 0	03:55	0.490	0.001 ± 0	02:29
0.985	0.002 ± 0	04:01	0.488	0.001 ± 0	03:54	0.978	0.002 ± 0	03:59	0.980	0.002 ± 0	02:33
1.969	0.004 ± 0	04:05	0.974	0.003 ± 0	03:58	1.954	0.005 ± 0	04:03	1.954	0.005 ± 0	02:37
3.932	0.008 ± 0	04:09	1.950	0.007 ± 0	04:02	3.914	0.010 ± 0	04:08	3.914	0.011 ± 0	02:41
5.967	0.012 ± 0	04:13	3.896	0.013 ± 0	04:06	5.959	0.015 ± 0	04:12	5.967	0.018 ± 0	02:45
7.961	0.016 ± 0	04:17	5.959	0.021 ± 0	04:10	7.958	0.020 ± 0	04:16	7.959	0.024 ± 0	02:50
10.177	0.017 ± 0	04:21	7.963	0.028 ± 0	04:14	10.158	0.023 ± 0	04:20	10.130	0.028 ± 0	02:55
10.930	0.019 ± 0	04:25	10.175	0.033 ± 0	04:18	10.924	0.025 ± 0	04:24	10.926	0.031 ± 0	02:59
12.155	0.020 ± 0	04:32	10.924	0.035 ± 0	04:23	12.032	0.026 ± 0	04:30	12.192	0.033 ± 0	03:03
13.293	0.037 ± 0	04:36	12.165	0.040 ± 0	04:28	13.151	0.043 ± 0	04:34	13.158	0.049 ± 0	03:08
15.212	0.043 ± 0	04:42	13.291	0.057 ± 0	04:33	15.344	0.048 ± 0	04:39	15.344	0.054 ± 0	03:12
17.786	0.048 ± 0	04:48	15.212	0.063 ± 0	04:38	17.377	0.052 ± 0	04:46	17.381	0.060 ± 0	03:17
19.985	0.052 ± 0	04:55	17.432	0.070 ± 0	04:44	20.033	0.059 ± 0	04:53	19.955	0.068 ± 0	03:21
22.514	0.057 ± 0	05:02	19.922	0.077 ± 0	04:51	22.423	0.064 ± 0	05:00	22.468	0.075 ± 0	03:26
24.927	0.061 ± 0	05:09	22.659	0.085 ± 0	04:58	25.198	0.070 ± 0	05:07	24.925	0.082 ± 0	03:31
27.582	0.066 ± 0	05:13	24.914	0.092 ± 0	05:05	27.414	0.075 ± 0	05:11	27.455	0.089 ± 0	03:34
29.915	0.069 ± 0	05:18	27.671	0.101 ± 0	05:10	30.064	0.081 ± 0	05:16	30.057	0.096 ± 0	03:39
35.144	0.078 ± 0	05:23	29.906	0.108 ± 0	05:15	34.833	0.092 ± 0	05:22	34.789	0.108 ± 0	03:44
39.936	0.087 ± 0	05:30	35.128	0.123 ± 0	05:20	40.259	0.104 ± 0	05:27	40.092	0.122 ± 0	03:49
45.219	0.096 ± 0	05:35	39.876	0.136 ± 0	05:25	44.881	0.114 ± 0	05:34	44.912	0.134 ± 0	03:54
50.113	0.104 ± 0	05:39	45.183	0.151 ± 0	05:32	49.876	0.125 ± 0	05:38	50.157	0.148 ± 0	03:58
74.507	0.143 ± 0	05:43	49.882	0.164 ± 0	05:37	74.099	0.173 ± 0	05:42	73.833	0.206 ± 0	04:03
99.658	0.181 ± 0	05:47	73.810	0.225 ± 0	05:41	100.195	0.221 ± 0	05:46	99.901	0.266 ± 0.001	04:08
124.511	0.217 ± 0	05:51	99.920	0.287 ± 0.001	05:44	125.188	0.264 ± 0.001	05:49	125.214	0.321 ± 0.001	04:11
149.733	0.252 ± 0.001	05:54	124.803	0.341 ± 0.001	05:48	149.829	0.305 ± 0.001	05:53	150.342	0.374 ± 0.001	04:15
174.979	0.286 ± 0.001	05:58	149.742	0.392 ± 0.001	05:52	174.743	0.343 ± 0.001	05:56	175.251	0.423 ± 0.001	04:19
199.325	0.318 ± 0.001	06:02	174.992	0.441 ± 0.001	05:55	199.343	0.380 ± 0.001	06:00	199.417	0.469 ± 0.001	04:22
298.125	0.434 ± 0.001	06:05	200.164	0.487 ± 0.001	05:59	298.106	0.509 ± 0.001	06:04	296.695	0.639 ± 0.001	04:26
398.578	0.542 ± 0.001	06:09	297.386	0.644 ± 0.001	06:03	398.957	0.623 ± 0.001	06:08	399.445	0.798 ± 0.002	04:30
498.563	0.641 ± 0.001	06:13	400.243	0.786 ± 0.001	06:06	498.618	0.721 ± 0.001	06:11	499.192	0.939 ± 0.002	04:33
598.845	0.732 ± 0.001	06:16	498.740	0.905 ± 0.002	06:10	598.739	0.809 ± 0.002	06:15	599.612	1.070 ± 0.002	04:37
698.713	0.818 ± 0.002	06:20	598.621	1.012 ± 0.002	06:14	698.844	0.889 ± 0.002	06:18	698.746	1.191 ± 0.002	04:40
798.883	0.899 ± 0.002	06:24	698.733	1.109 ± 0.002	06:17	798.977	0.963 ± 0.002	06:22	799.135	1.305 ± 0.002	04:44

898.886	0.977 ± 0.002	06:27	798.674	1.198 ± 0.002	06:21	898.965	1.031 ± 0.002	06:26	899.839	1.416 ± 0.003	04:47
948.895	1.015 ± 0.002	06:31	898.680	1.282 ± 0.002	06:25	948.847	1.064 ± 0.002	06:29	949.280	1.469 ± 0.003	04:51
802.269	0.911 ± 0.002	06:34	948.680	1.323 ± 0.002	06:28	801.689	0.973 ± 0.002	06:33	804.310	1.322 ± 0.003	04:54
701.765	0.831 ± 0.002	06:38	803.865	1.210 ± 0.002	06:32	701.482	0.903 ± 0.002	06:37	702.767	1.209 ± 0.002	04:58
600.073	0.745 ± 0.002	06:42	702.557	1.123 ± 0.002	06:36	600.352	0.822 ± 0.002	06:40	600.694	1.086 ± 0.002	05:02
500.691	0.653 ± 0.001	06:45	600.772	1.024 ± 0.002	06:39	500.799	0.734 ± 0.001	06:44	501.264	0.957 ± 0.002	05:06
400.231	0.554 ± 0.001	06:49	501.448	0.917 ± 0.002	06:43	400.418	0.635 ± 0.001	06:48	400.792	0.815 ± 0.002	05:09
300.551	0.447 ± 0.001	06:53	400.866	0.796 ± 0.001	06:47	300.808	0.522 ± 0.001	06:52	300.448	0.660 ± 0.001	05:13
200.466	0.329 ± 0.001	06:58	300.733	0.659 ± 0.001	06:50	200.614	0.391 ± 0.001	06:56	200.712	0.486 ± 0.001	05:17
175.186	0.296 ± 0.001	07:01	200.694	0.498 ± 0.001	06:54	175.598	0.353 ± 0.001	07:00	175.538	0.437 ± 0.001	05:22
150.160	0.262 ± 0.001	07:05	175.215	0.451 ± 0.001	06:59	150.254	0.313 ± 0.001	07:04	150.246	0.386 ± 0.001	05:25
125.464	0.226 ± 0	07:10	150.236	0.403 ± 0.001	07:03	125.466	0.271 ± 0.001	07:09	125.536	0.334 ± 0.001	05:29
100.347	0.189 ± 0	07:14	125.305	0.351 ± 0.001	07:07	100.373	0.227 ± 0	07:13	100.351	0.277 ± 0.001	05:34
75.338	0.150 ± 0	07:19	100.353	0.295 ± 0.001	07:11	75.367	0.180 ± 0	07:17	75.329	0.219 ± 0	05:38
50.284	0.111 ± 0	07:23	75.434	0.236 ± 0	07:16	50.336	0.131 ± 0	07:22	50.321	0.158 ± 0	05:42
45.383	0.101 ± 0	07:28	50.378	0.171 ± 0	07:20	45.382	0.120 ± 0	07:26	45.355	0.144 ± 0	05:47
40.275	0.092 ± 0	07:32	45.351	0.157 ± 0	07:24	40.271	0.108 ± 0	07:30	40.249	0.130 ± 0	05:50
35.337	0.082 ± 0	07:36	40.289	0.142 ± 0	07:29	35.357	0.097 ± 0	07:35	35.380	0.116 ± 0	05:54
30.360	0.072 ± 0	07:42	35.347	0.127 ± 0	07:34	30.371	0.085 ± 0	07:40	30.315	0.101 ± 0	05:59
25.376	0.062 ± 0	07:47	30.365	0.111 ± 0	07:37	25.347	0.073 ± 0	07:44	25.312	0.087 ± 0	06:04
20.380	0.052 ± 0	07:52	25.356	0.096 ± 0	07:43	20.384	0.061 ± 0	07:51	20.365	0.073 ± 0	06:09
17.885	0.047 ± 0	07:57	20.394	0.080 ± 0	07:49	17.901	0.054 ± 0	07:56	17.914	0.066 ± 0	06:14
15.429	0.042 ± 0	08:02	17.891	0.072 ± 0	07:54	15.418	0.048 ± 0	08:00	15.402	0.057 ± 0	06:19
13.910	0.039 ± 0	08:06	15.414	0.063 ± 0	07:59	13.927	0.044 ± 0	08:04	13.899	0.053 ± 0	06:24
12.962	0.030 ± 0	08:09	13.926	0.058 ± 0	08:03	12.940	0.034 ± 0	08:09	12.850	0.044 ± 0	06:29
11.567	0.018 ± 0	08:14	12.896	0.048 ± 0	08:07	11.596	0.022 ± 0	08:13	11.457	0.033 ± 0	06:33
11.047	0.023 ± 0	08:18	11.500	0.037 ± 0	08:11	10.988	0.026 ± 0	08:17	10.083	0.034 ± 0	06:38
10.065	0.020 ± 0	08:23	10.104	0.037 ± 0	08:15	10.079	0.024 ± 0	08:21	8.150	0.027 ± 0	06:44
8.046	0.016 ± 0	08:27	8.166	0.030 ± 0	08:20	8.073	0.018 ± 0	08:26	6.042	0.019 ± 0	06:55
6.015	0.011 ± 0	08:33	6.036	0.022 ± 0	08:27	6.063	0.013 ± 0	08:32	4.048	0.011 ± 0	07:27
4.039	0.006 ± 0	08:49	4.018	0.014 ± 0	08:42	4.086	0.006 ± 0	08:47	2.076	5.36±0E-05	09:37
			2.054	0.005 ± 0	09:29						
			1.025	1.9±0E-04	10:26						

Table S10. CH_4 adsorption data on carbon materials at 323.15 K

BPL Carbon			C564			C569			C1005		
Absolute Pressure (mbar)	n (mmol/g)	Elapsed Time (h:min)	Absolute Pressure (mbar)	n (mmol/g)	Elapsed Time (h:min)	Absolute Pressure (mbar)	n (mmol/g)	Elapsed Time (h:min)	Absolute Pressure (mbar)	n (mmol/g)	Elapsed Time (h:min)
1.001	1.44±0E-04	03:35	0.496	5.31E-05	03:32	1.001	3.3±0E-04	03:38	1.993	0.001 ± 0	02:35
2.003	0.001 ± 0	03:39	0.994	0.001 ± 0	03:36	2.003	0.001 ± 0	03:42	3.998	0.003 ± 0	02:40
3.993	0.002 ± 0	03:43	1.995	0.002 ± 0	03:40	3.998	0.003 ± 0	03:46	6.010	0.005 ± 0	02:44
5.992	0.003 ± 0	03:48	3.993	0.004 ± 0	03:45	5.971	0.004 ± 0	03:50	8.027	0.007 ± 0	02:48
7.972	0.004 ± 0	03:52	5.986	0.006 ± 0	03:49	7.999	0.006 ± 0	03:55	10.176	0.007 ± 0	02:52
10.189	0.003 ± 0	03:56	7.969	0.008 ± 0	03:53	10.182	0.005 ± 0	03:59	10.957	0.008 ± 0	02:56
10.977	0.003 ± 0	04:01	10.196	0.008 ± 0	03:57	10.999	0.006 ± 0	04:03	12.168	0.008 ± 0	03:00
12.191	0.002 ± 0	04:05	10.991	0.009 ± 0	04:02	12.176	0.005 ± 0	04:07	13.195	0.021 ± 0	03:05
13.189	0.018 ± 0	04:09	12.176	0.009 ± 0	04:06	13.198	0.021 ± 0	04:11	15.253	0.023 ± 0	03:09
15.204	0.019 ± 0	04:13	13.207	0.024 ± 0	04:10	15.232	0.023 ± 0	04:15	17.394	0.025 ± 0	03:14
17.461	0.024 ± 0	04:18	15.224	0.026 ± 0	04:14	17.452	0.024 ± 0	04:22	19.963	0.028 ± 0	03:18
20.027	0.026 ± 0	04:24	17.445	0.028 ± 0	04:20	19.967	0.026 ± 0	04:27	22.460	0.030 ± 0	03:22
22.436	0.028 ± 0	04:29	20.001	0.031 ± 0	04:26	22.460	0.028 ± 0	04:33	24.944	0.032 ± 0	03:27
24.915	0.029 ± 0	04:34	22.512	0.033 ± 0	04:31	24.963	0.029 ± 0	04:39	27.433	0.034 ± 0	03:31
27.452	0.030 ± 0	04:41	24.945	0.034 ± 0	04:37	27.440	0.032 ± 0	04:44	29.921	0.037 ± 0	03:34
29.918	0.032 ± 0	04:46	27.474	0.037 ± 0	04:43	29.943	0.033 ± 0	04:49	34.939	0.042 ± 0	03:39
34.998	0.034 ± 0	04:51	29.930	0.040 ± 0	04:48	34.881	0.037 ± 0	04:55	39.933	0.047 ± 0	03:43
39.850	0.037 ± 0	04:56	34.951	0.045 ± 0	04:53	39.906	0.041 ± 0	05:00	44.934	0.052 ± 0	03:47
44.889	0.040 ± 0	05:01	39.857	0.050 ± 0	04:57	44.940	0.045 ± 0	05:05	49.900	0.057 ± 0	03:51
49.876	0.043 ± 0	05:07	44.920	0.055 ± 0	05:02	49.824	0.048 ± 0	05:10	74.393	0.081 ± 0	03:55
74.525	0.057 ± 0	05:11	49.938	0.059 ± 0	05:09	74.548	0.066 ± 0	05:14	99.887	0.105 ± 0	04:00
99.720	0.071 ± 0	05:16	74.510	0.081 ± 0	05:13	99.715	0.083 ± 0	05:18	124.643	0.128 ± 0	04:04
124.486	0.084 ± 0	05:20	99.785	0.104 ± 0	05:17	124.677	0.101 ± 0	05:22	149.570	0.151 ± 0	04:08
149.520	0.098 ± 0	05:24	124.872	0.126 ± 0	05:21	149.466	0.117 ± 0	05:26	174.429	0.173 ± 0	04:11
174.358	0.112 ± 0	05:27	149.846	0.147 ± 0	05:25	174.942	0.134 ± 0	05:29	199.531	0.195 ± 0	04:15
199.588	0.125 ± 0	05:31	174.841	0.167 ± 0	05:28	199.429	0.149 ± 0	05:33	298.212	0.277 ± 0.001	04:18
298.332	0.176 ± 0	05:35	199.286	0.187 ± 0	05:32	297.959	0.205 ± 0	05:37	399.219	0.355 ± 0.001	04:22
399.392	0.225 ± 0	05:38	298.131	0.259 ± 0.001	05:36	398.717	0.259 ± 0.001	05:40	499.552	0.426 ± 0.001	04:25
498.653	0.269 ± 0.001	05:42	398.984	0.326 ± 0.001	05:39	499.522	0.307 ± 0.001	05:44	601.383	0.494 ± 0.001	04:29
599.374	0.311 ± 0.001	05:45	499.424	0.386 ± 0.001	05:43	600.679	0.351 ± 0.001	05:47	700.532	0.557 ± 0.001	04:33
700.613	0.352 ± 0.001	05:49	600.769	0.443 ± 0.001	05:46	700.992	0.392 ± 0.001	05:51	800.869	0.617 ± 0.001	04:37
800.881	0.390 ± 0.001	05:52	700.945	0.495 ± 0.001	05:50	801.015	0.429 ± 0.001	05:55	901.749	0.676 ± 0.001	04:40
901.676	0.426 ± 0.001	05:56	800.667	0.543 ± 0.001	05:53	901.218	0.465 ± 0.001	05:58	950.928	0.705 ± 0.001	04:44

950.309	0.443 ± 0.001	06:00	900.698	0.590 ± 0.001	05:57	950.340	0.482 ± 0.001	06:02	801.233	0.623 ± 0.001	04:47
800.413	0.393 ± 0.001	06:03	950.569	0.613 ± 0.001	06:01	800.779	0.436 ± 0.001	06:05	701.015	0.563 ± 0.001	04:51
700.749	0.356 ± 0.001	06:07	801.001	0.549 ± 0.001	06:04	700.627	0.399 ± 0.001	06:09	600.593	0.498 ± 0.001	04:55
599.794	0.313 ± 0.001	06:11	700.936	0.500 ± 0.001	06:08	599.905	0.358 ± 0.001	06:13	500.086	0.429 ± 0.001	04:59
499.974	0.269 ± 0.001	06:14	599.983	0.447 ± 0.001	06:12	500.239	0.313 ± 0.001	06:16	400.121	0.357 ± 0.001	05:02
400.060	0.222 ± 0	06:18	500.160	0.389 ± 0.001	06:15	400.678	0.265 ± 0.001	06:20	300.338	0.279 ± 0.001	05:06
300.100	0.173 ± 0	06:22	400.753	0.328 ± 0.001	06:19	300.283	0.211 ± 0	06:24	200.400	0.197 ± 0	05:10
200.255	0.122 ± 0	06:26	300.334	0.260 ± 0.001	06:23	200.332	0.153 ± 0	06:28	175.465	0.174 ± 0	05:13
175.660	0.107 ± 0	06:30	200.424	0.186 ± 0	06:27	175.706	0.136 ± 0	06:32	150.612	0.151 ± 0	05:17
150.464	0.094 ± 0	06:34	175.725	0.165 ± 0	06:31	150.519	0.120 ± 0	06:36	125.372	0.127 ± 0	05:21
125.434	0.079 ± 0	06:38	150.657	0.144 ± 0	06:35	125.568	0.102 ± 0	06:40	100.348	0.103 ± 0	05:24
100.479	0.064 ± 0	06:42	125.573	0.121 ± 0	06:39	100.433	0.084 ± 0	06:45	75.355	0.078 ± 0	05:29
75.410	0.048 ± 0	06:46	100.431	0.098 ± 0	06:43	75.309	0.065 ± 0	06:50	50.352	0.053 ± 0	05:33
50.344	0.034 ± 0	06:51	75.410	0.074 ± 0	06:48	50.271	0.046 ± 0	06:55	45.466	0.048 ± 0	05:37
45.447	0.031 ± 0	06:57	50.391	0.051 ± 0	06:53	45.401	0.041 ± 0	07:01	40.407	0.043 ± 0	05:41
40.416	0.028 ± 0	07:03	45.507	0.045 ± 0	06:59	40.410	0.037 ± 0	07:07	35.418	0.036 ± 0	05:46
35.408	0.025 ± 0	07:09	40.456	0.040 ± 0	07:04	35.382	0.032 ± 0	07:13	30.431	0.030 ± 0	05:50
30.391	0.021 ± 0	07:14	35.452	0.033 ± 0	07:11	30.373	0.028 ± 0	07:19	25.453	0.025 ± 0	05:54
25.381	0.017 ± 0	07:20	30.454	0.028 ± 0	07:16	25.412	0.024 ± 0	07:25	20.492	0.020 ± 0	05:58
20.425	0.014 ± 0	07:27	25.447	0.022 ± 0	07:23	20.421	0.020 ± 0	07:32	17.938	0.017 ± 0	06:03
17.912	0.012 ± 0	07:34	20.445	0.016 ± 0	07:30	17.958	0.018 ± 0	07:39	15.478	0.014 ± 0	06:07
15.460	0.009 ± 0	07:41	17.982	0.014 ± 0	07:36	15.456	0.015 ± 0	07:46	13.961	0.012 ± 0	06:12
13.940	0.008 ± 0	07:48	15.470	0.010 ± 0	07:43	13.971	0.013 ± 0	07:52	12.884	0.006 ± 0	06:16
12.925	0.001 ± 0	07:53	13.943	0.009 ± 0	07:50	12.933	0.006 ± 0	07:55			
			12.926	0.001 ± 0	07:54						

Table S11. Water adsorption data on carbon materials at 323.15 K

BPL Carbon			C564			C569			C1005		
Absolute Pressure (mbar)	n (mmol/g)	Elapsed Time (h:min)	Absolute Pressure (mbar)	n (mmol/g)	Elapsed Time (h:min)	Absolute Pressure (mbar)	n (mmol/g)	Elapsed Time (h:min)	Absolute Pressure (mbar)	n (mmol/g)	Elapsed Time (h:min)
0.005	0.031 ± 0	03:20	6.02E-05	1.74±0E-04	02:57	6.94E-05	3.18±0E-04	02:38	8.02E-05	2.96±0E-04	03:03
0.010	0.046 ± 0	03:46	8.03E-05	1.77±0E-04	03:02	8.94E-05	3.41±0E-08	02:41	1.6E-04	0.001 ± 0	03:15
0.015	0.059 ± 0	04:09	1.50E-04	0.001 ± 0	03:10	1.69E-04	0.001 ± 0	02:47	3.16E-04	0.002 ± 0	03:31
0.020	0.069 ± 0	04:26	3.59E-04	0.002 ± 0	03:24	0.000	0.001 ± 0	02:55	0.001	0.005 ± 0	03:53
0.025	0.080 ± 0	04:41	0.001	0.003 ± 0	03:36	0.001	0.003 ± 0	03:07	0.001	0.007 ± 0	04:19
0.030	0.091 ± 0	04:54	0.001	0.005 ± 0	03:56	0.001	0.006 ± 0	03:21	0.003	0.012 ± 0	04:51
0.039	0.110 ± 0	05:10	0.003	0.009 ± 0	04:14	0.003	0.010 ± 0	03:35	0.005	0.019 ± 0	05:27
0.049	0.131 ± 0	05:26	0.005	0.014 ± 0	04:38	0.005	0.018 ± 0	03:50	0.010	0.031 ± 0	06:12
0.057	0.148 ± 0	05:37	0.010	0.024 ± 0	05:08	0.010	0.032 ± 0	04:07	0.015	0.039 ± 0	06:40
0.068	0.166 ± 0	05:45	0.015	0.031 ± 0	05:29	0.015	0.042 ± 0	04:20	0.020	0.046 ± 0	07:01
0.080	0.188 ± 0	05:56	0.020	0.039 ± 0	05:50	0.020	0.054 ± 0	04:34	0.024	0.053 ± 0	07:24
0.091	0.207 ± 0	06:04	0.025	0.046 ± 0	06:06	0.025	0.064 ± 0	04:46	0.030	0.059 ± 0	07:40
0.101	0.232 ± 0	06:10	0.030	0.053 ± 0	06:20	0.030	0.073 ± 0	04:57	0.039	0.069 ± 0	08:01
0.081	0.200 ± 0	06:14	0.039	0.064 ± 0	06:36	0.039	0.090 ± 0	05:10	0.049	0.080 ± 0	08:18
0.061	0.173 ± 0	06:19	0.050	0.077 ± 0	06:49	0.049	0.110 ± 0	05:23	0.058	0.088 ± 0	08:31
0.041	0.146 ± 0	06:27	0.058	0.087 ± 0	07:01	0.058	0.124 ± 0	05:31	0.068	0.097 ± 0	08:42
0.025	0.124 ± 0	06:43	0.068	0.099 ± 0	07:11	0.068	0.140 ± 0	05:41	0.081	0.107 ± 0	08:55
0.010	0.092 ± 0	08:01	0.081	0.110 ± 0	07:22	0.080	0.159 ± 0	05:53	0.091	0.114 ± 0	09:04
0.005	0.071 ± 0	10:41	0.091	0.120 ± 0	07:30	0.091	0.173 ± 0	06:00	0.101	0.129 ± 0	09:12
			0.101	0.137 ± 0	07:35	0.101	0.196 ± 0	06:06	0.111	0.143 ± 0	09:19
			0.111	0.154 ± 0	07:41	0.110	0.222 ± 0	06:14	0.124	0.154 ± 0	09:27
			0.124	0.169 ± 0	07:48	0.124	0.242 ± 0.001	06:21	0.149	0.176 ± 0	09:37
			0.150	0.196 ± 0	07:57	0.149	0.282 ± 0.001	06:31	0.170	0.197 ± 0	09:47
			0.172	0.221 ± 0	08:04	0.172	0.317 ± 0.001	06:39	0.195	0.225 ± 0	10:00
			0.197	0.254 ± 0	08:13	0.196	0.363 ± 0.001	06:50	0.104	0.180 ± 0	10:08
			0.104	0.189 ± 0	08:19	0.102	0.254 ± 0.001	06:58	0.084	0.151 ± 0	10:17
			0.083	0.152 ± 0	08:27	0.078	0.217 ± 0	07:06	0.056	0.136 ± 0	10:34
			0.054	0.134 ± 0	08:40	0.060	0.189 ± 0	07:18	0.040	0.122 ± 0	10:54
			0.041	0.120 ± 0	08:54	0.042	0.160 ± 0	07:39	0.025	0.107 ± 0	11:41
			0.025	0.102 ± 0	09:33	0.024	0.125 ± 0	08:30	0.010	0.081 ± 0	14:18
			0.010	0.076 ± 0	11:55	0.010	0.078 ± 0	11:18	0.005	0.059 ± 0	19:36
			0.005	0.054 ± 0	16:44	0.005	0.039 ± 0	17:31			

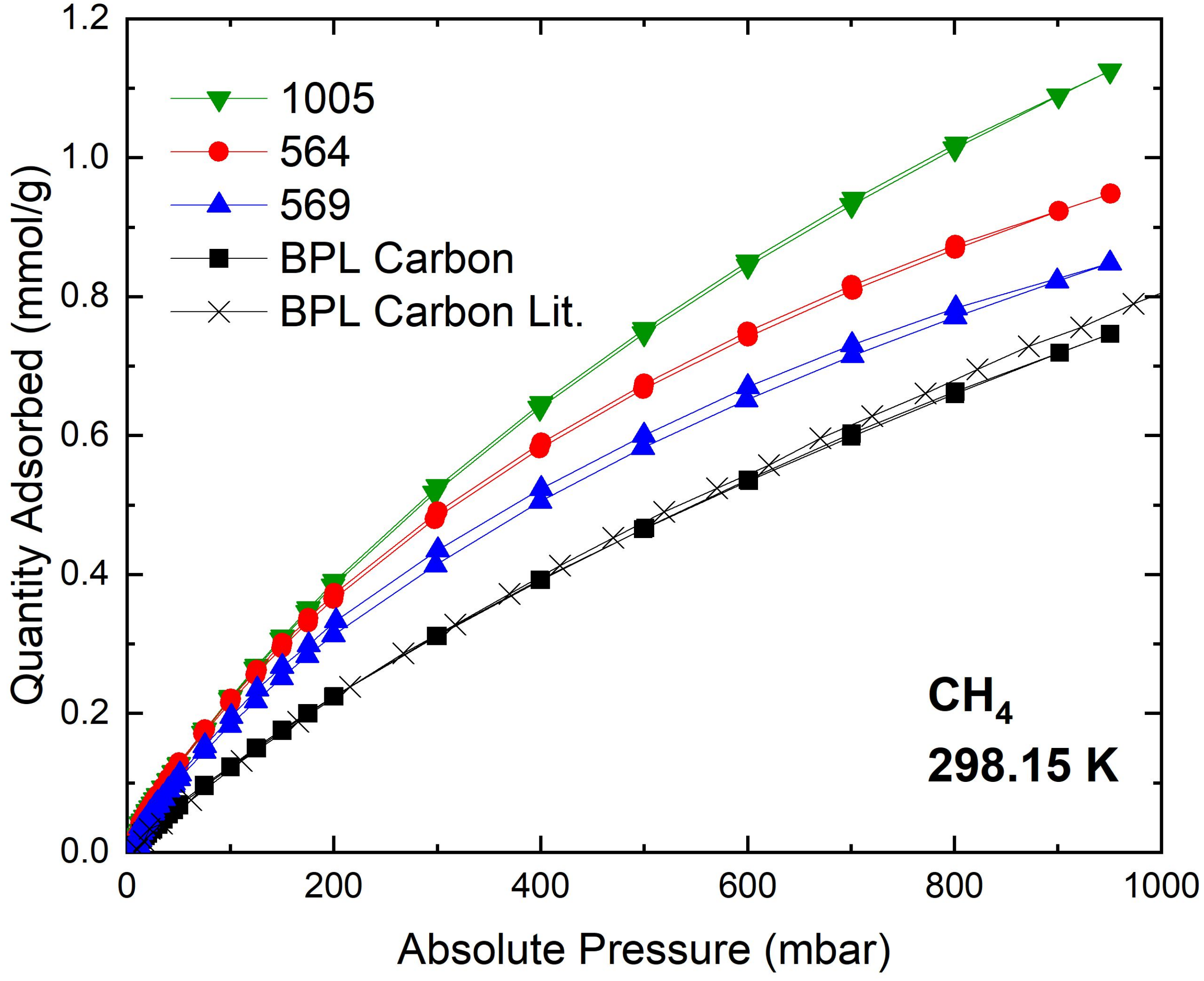

Figure S12. CH_4 isotherms on BPL Carbon, C564, C569, C1005, and BPL Carbon data from literature at 298.15 K. Adsorption and desorption data overlap.

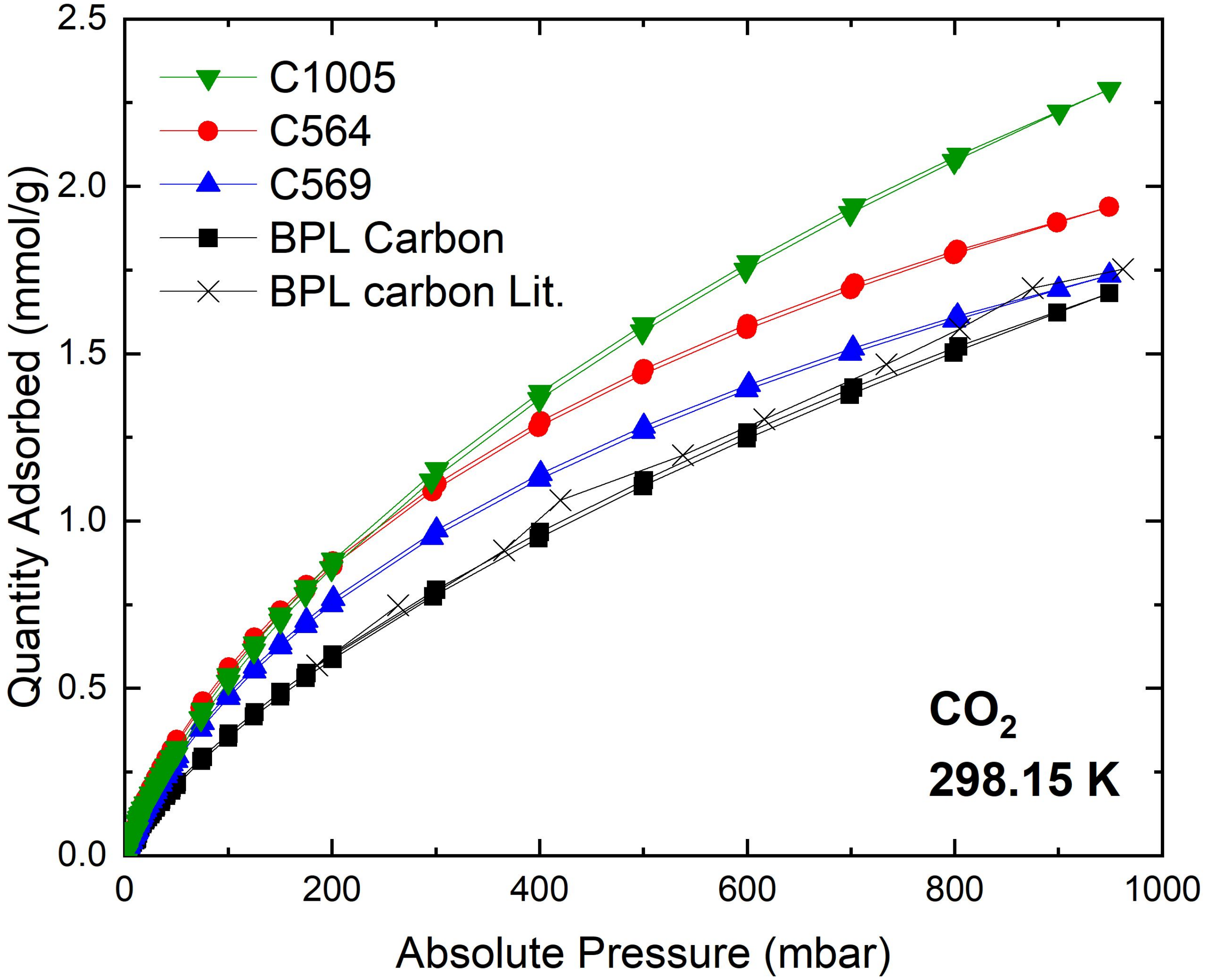

Figure S13. CO_2 isotherms on BPL Carbon, C564, C569, C1005, and BPL Carbon data from literature at 298.15 K. Adsorption and desorption data overlap.

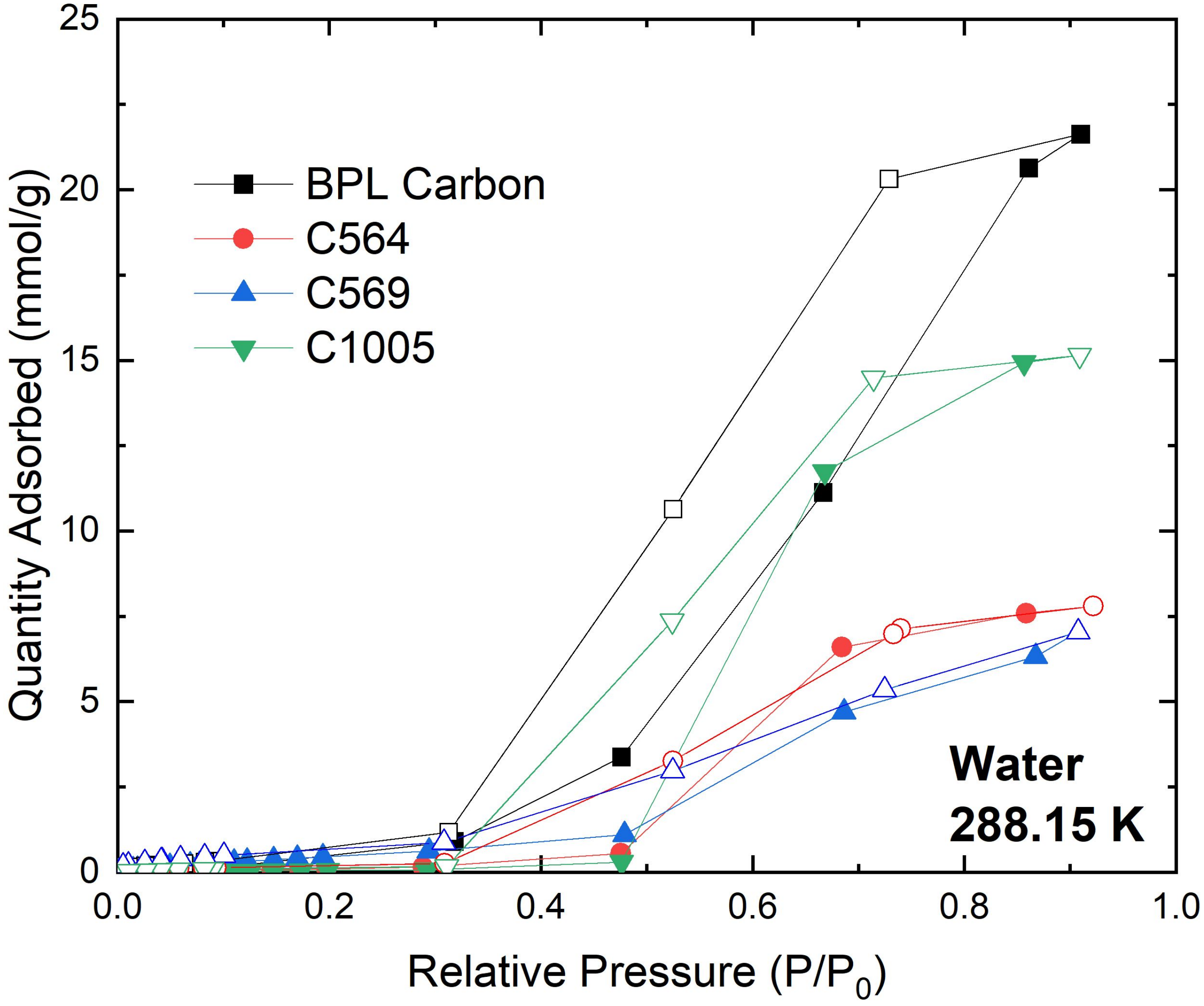

Figure S14. Water isotherms on BPL Carbon, C564, C569, and C1005 at 288.15 K. Closed symbols represent adsorption data, open symbols represent desorption data.

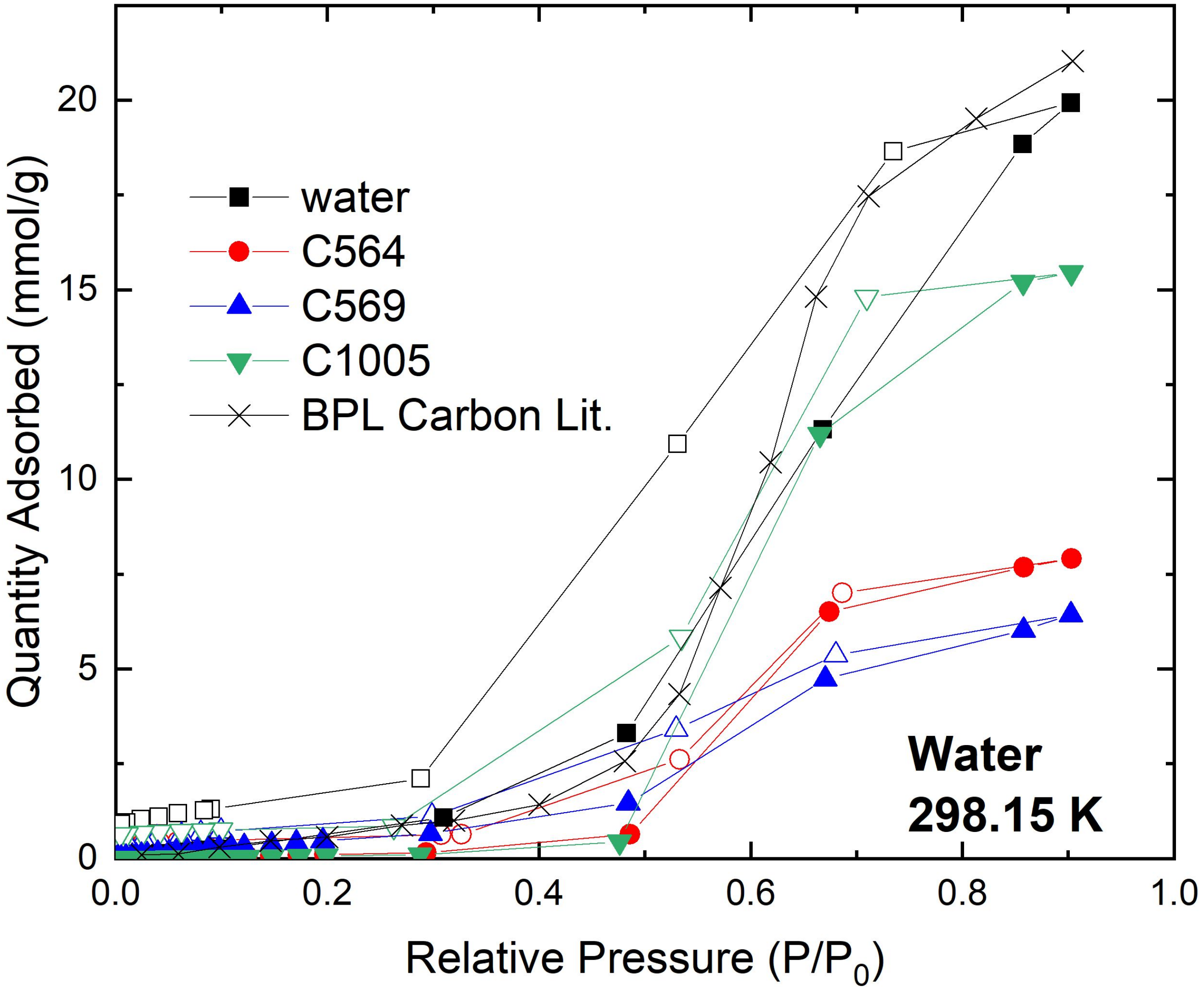

Figure S15. Water isotherms on BPL Carbon, C564, C569, C1005, and BPL Carbon data from literature at 298.15 K. Closed symbols represent adsorption data, open symbols represent desorption data.

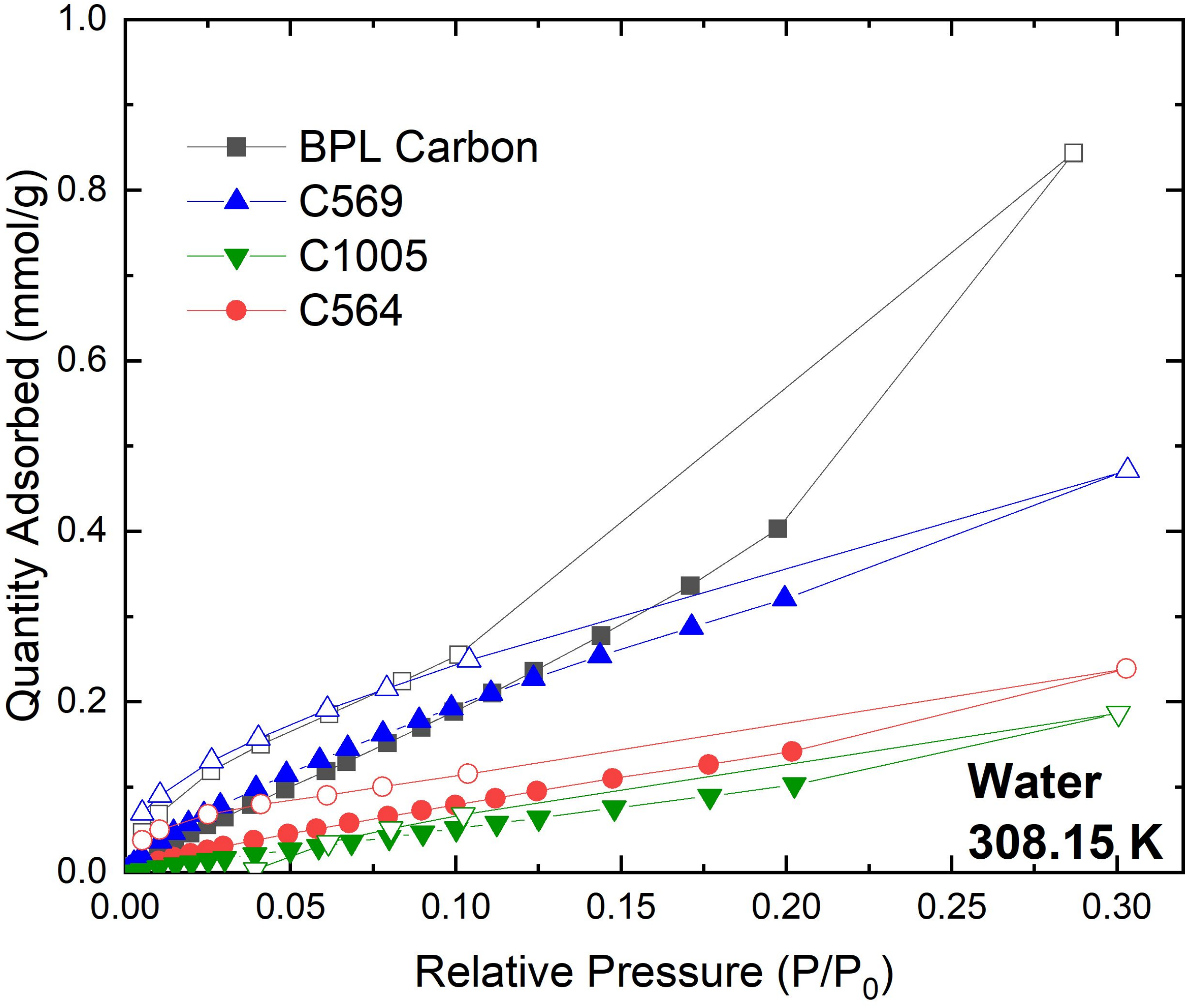

Figure S16. Water isotherms on BPL Carbon, C564, C569, and C1005 at 308.15 K. Closed symbols represent adsorption data, open symbols represent desorption data.

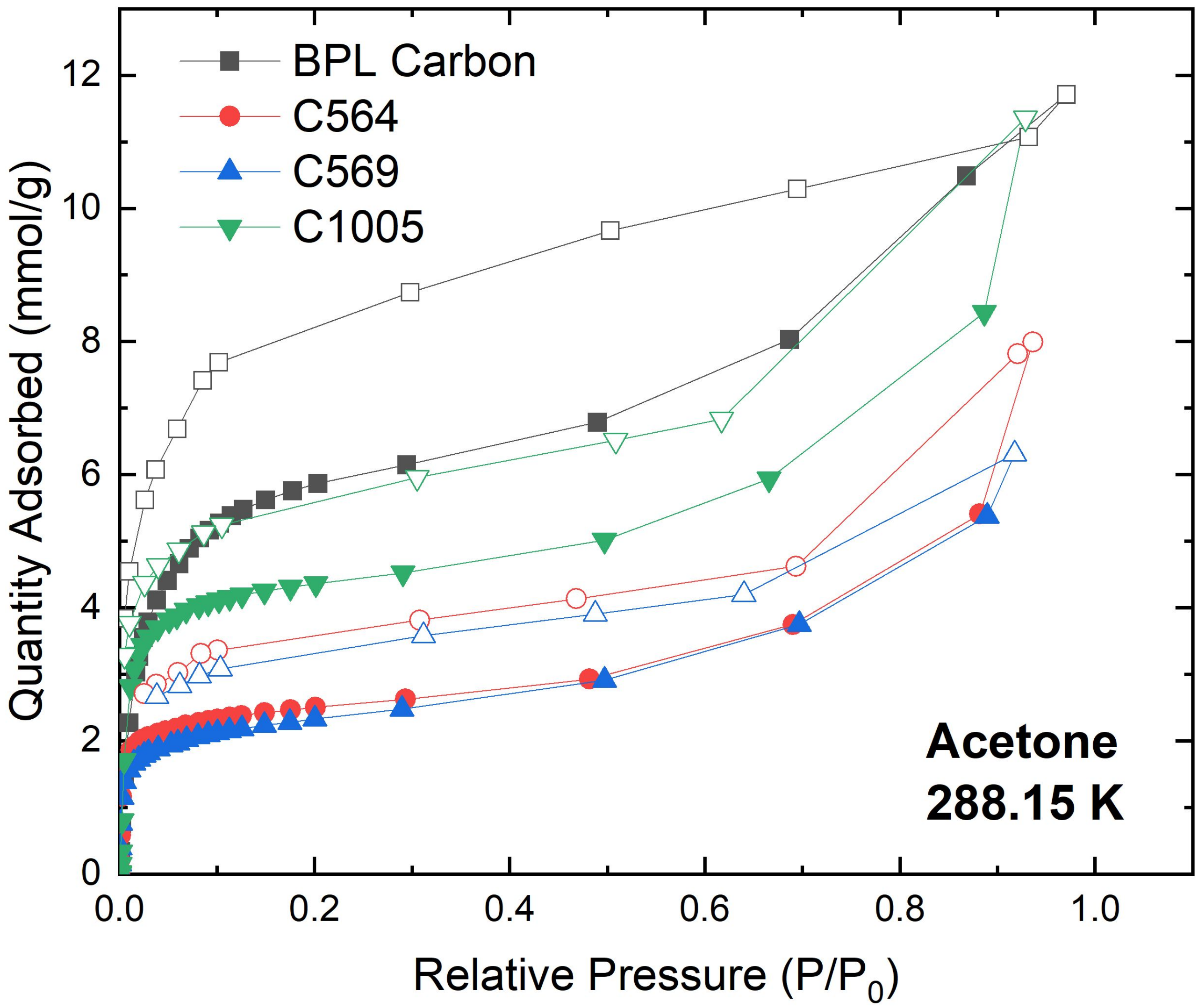

Figure S17. Acetone isotherms on BPL Carbon, C564, C569, and C1005 at 288.15 K. Closed symbols represent adsorption data, open symbols represent desorption data.

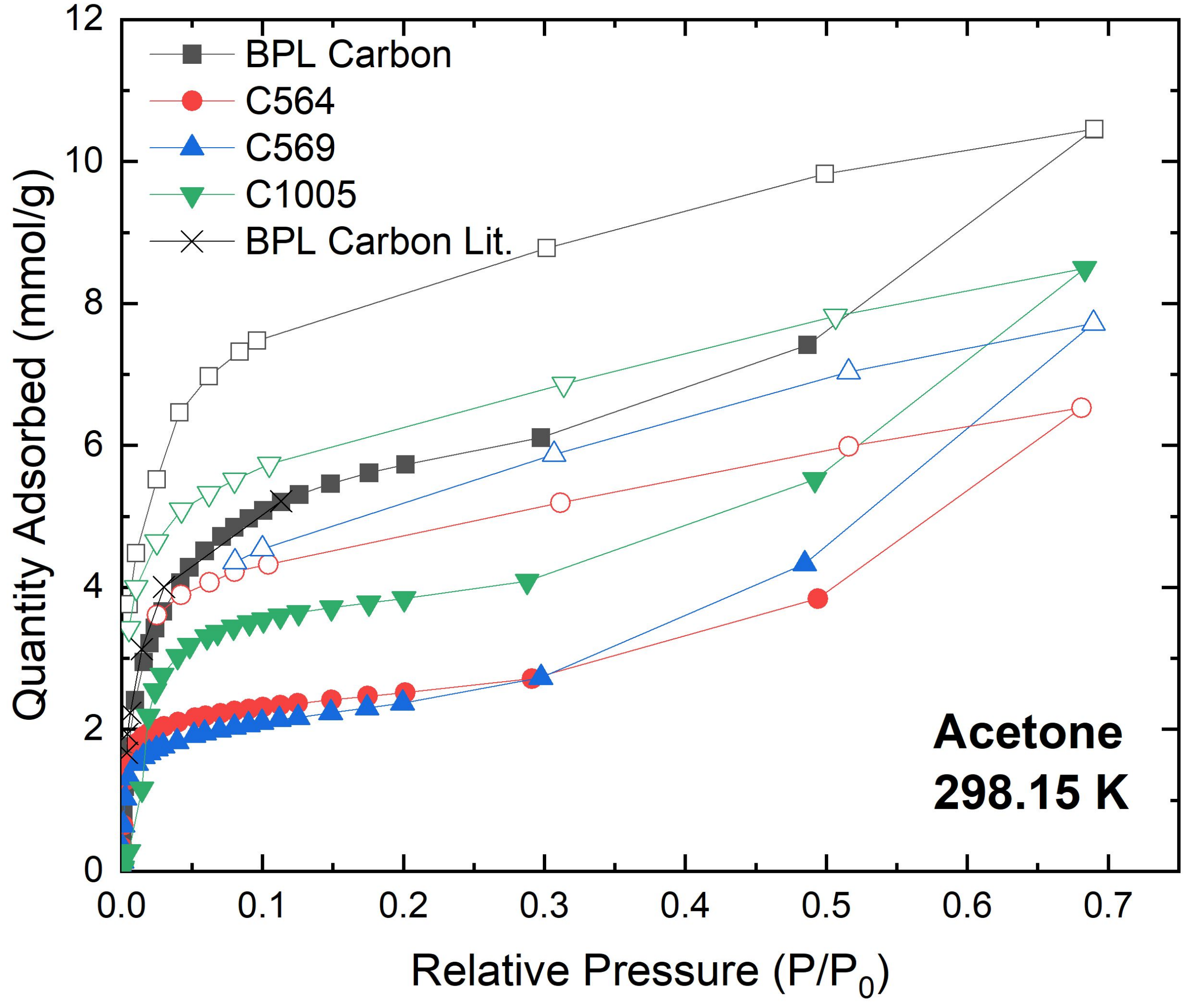

Figure S18. Acetone isotherms on BPL Carbon, C564, C569, C1005, and BPL Carbon data from literature at 298.15 K. Closed symbols represent adsorption data, open symbols represent desorption data.

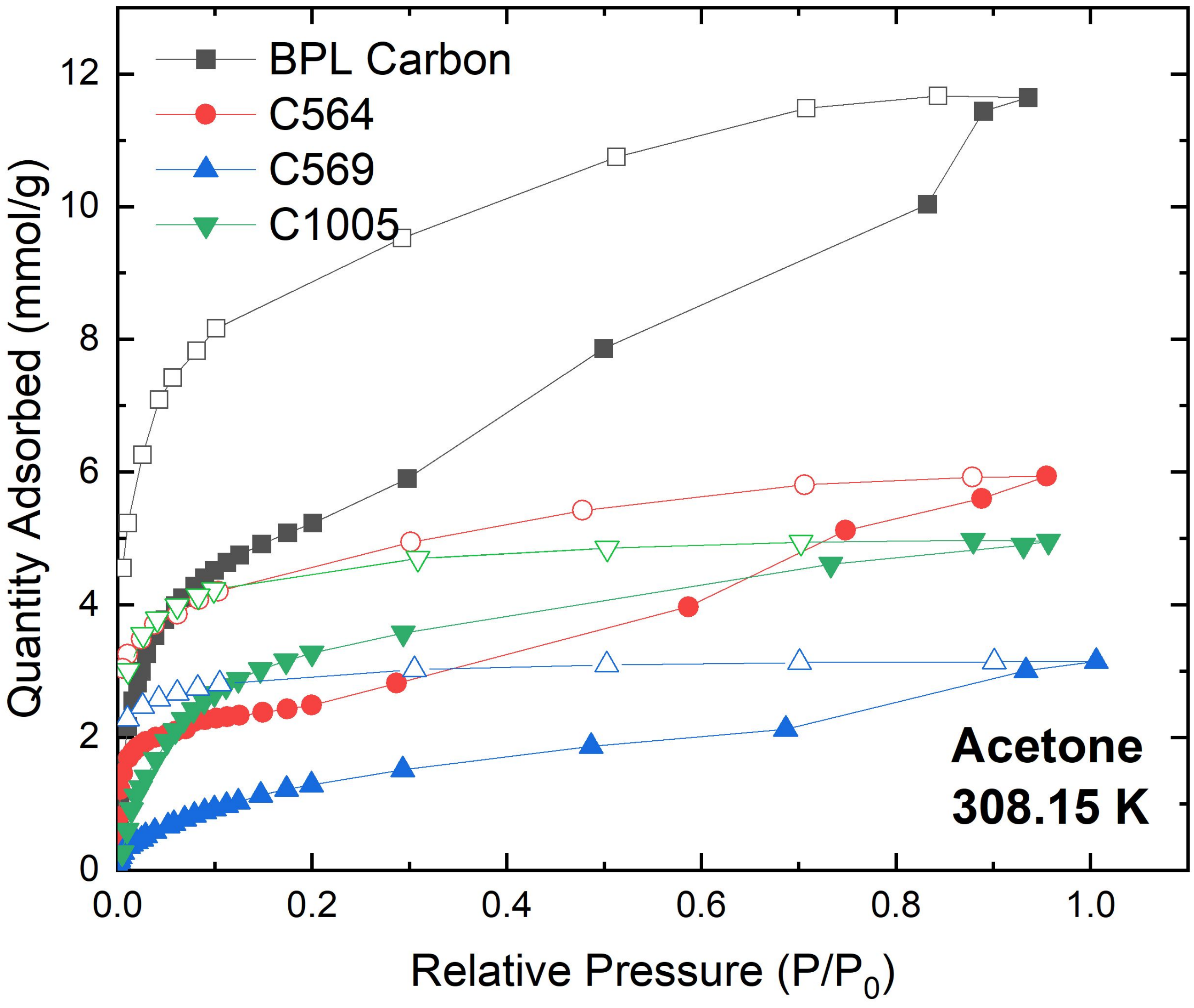

Figure S19. Acetone isotherms on BPL Carbon, C564, C569, and C1005 at 308.15 K. Closed symbols represent adsorption data, open symbols represent desorption data.
